# Effects of affective priming through music on the use of emotion words

**DOI:** 10.1371/journal.pone.0214482

**Published:** 2019-04-16

**Authors:** Rosabel Yu Ling Tay, Bee Chin Ng

**Affiliations:** Linguistics and Multilingual Studies, School of Humanities, Nanyang Technological University, Singapore, Singapore; Anadolu University, TURKEY

## Abstract

Understanding how music can evoke emotions and in turn affect language use has significant implications not only in clinical settings but also in the emotional development of children. The relationship between music and emotion is an intricate one that has been closely studied. However, how the use of emotion words can be influenced by auditory priming is a question which is still not known. The main interest in this study was to examine how manipulation of mode and tempo in music affects the emotions induced and the subsequent effects on the use of emotion words. Fifty university students in Singapore were asked to select emotion words after exposure to various music excerpts. The results showed that major modes and faster tempos elicited greater responses for positive words and high arousal words respectively, while minor modes elicited more high arousal words and original tempos resulted in more positive words being selected. In the Major-Fast, Major-Slow and Minor-Slow conditions, positive correlations were found between the number of high arousal words and their rated intensities. Upon further analysis, categorization of emotion words differed from the circumplex model. Taken together, the findings highlight the prominence of affective auditory priming and allow us to better understand our emotive responses to music.

## Introduction

“What happens when the music stops? Where does it go? What's left? What sticks with people in the audience at the end of a performance? Is it a melody or a rhythm or a mood or an attitude? And how might that change their lives?”

(Michael Tilson Thomas 2012)

The questions above from Michael Tilson Thomas, director of the San Francisco Symphony, underscore the profound influence of music on us, on the way we feel and we express that through words as well as behaviour. Current research seems to validate the view that music and language share many parallels with each other, including acoustic properties and the ability to evoke emotions in people. The ability of music to evoke emotions in listeners is one of the main motivations why many people continue to produce and listen to music. In a similar vein, words with certain meanings and connotations are able to affect our emotions as well. However, despite the large number of studies on the relationship between music, emotion and language, not many studies have investigated the use of music as an affective prime and its priming effects on language use. The studies on the affective priming effects on language have focused largely on visual stimuli and language tasks that were based on semantics or syntax [1–3], and most of these studies were conducted with monolingual populations. As a departure from other studies in the field, this research investigates the differences in the selection of emotion words using auditory (musical) primes and extends our understanding of the effect of music on emotion beyond just an evaluation of a response.

### Defining ‘emotions’

**Fig 1:**
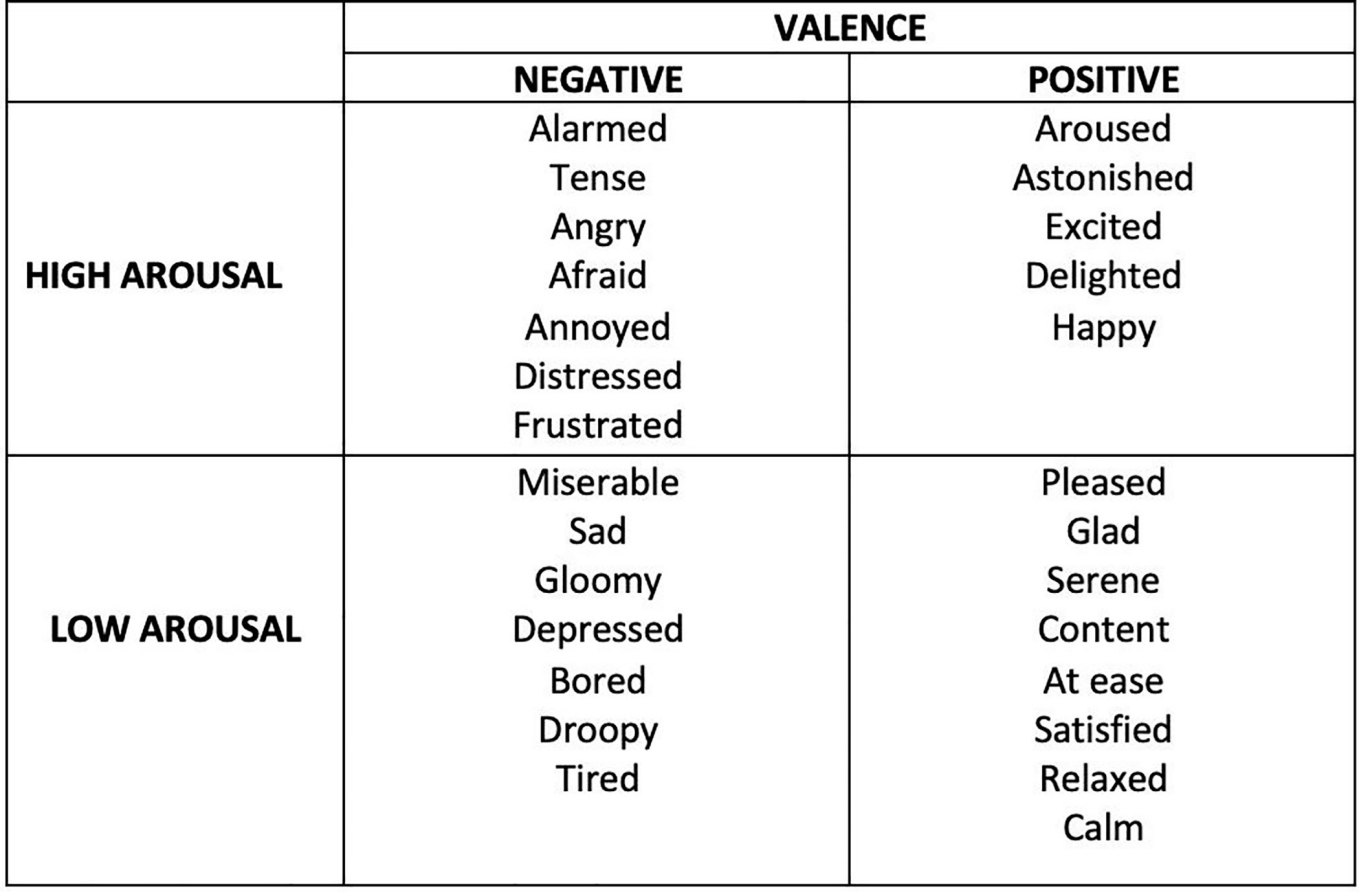
The four categories in the circumplex model and complete list of words.

The term ‘emotion’ is defined in a multitude of ways and scholars are still debating what constitutes an ‘emotion’. Despite these differences in definitions, the consensus across all theoretical approaches is that emotions are experienced and expressed in a variety of ways. The present study will adopt one of the dimensional models known as the circumplex model of affect [4]. This model lends itself to quantification and measurement and, therefore, it is often used for testing affective states [5]. The circumplex model places emotional experiences on two axes: one of valence (pleasant-unpleasant continuum) and the other of arousal (the physiological and psychological state of reacting to stimuli, or alertness continuum). Russell [6] classified 28 affective states into a spatial model (see complete list in Fig 1). The results showed that the placement of emotion words were largely consistent with the findings.

### How emotions influence language

The relationship between language and emotion and how emotions influence different aspects (prosodic, semantic and syntactic) of language has also been well-studied. Bänziger and Scherer [7] found that the levels of arousal underlying the portrayed emotions affect pitch contours of produced speech. For example, expressions produced with joy and anger had greater and faster F0 (fundamental frequency) changes compared to those produced with sadness or fear. Other studies have shown that the differences in prosody between infant- and adult-directed speech were a direct result of the differences in emotional expression [8]. The emotional expression helps to build a stronger emotional bond between caregiver and child [9].

There is also evidence showing that the appearance of an emotion word in a sentence can significantly impact structural processing of the sentence [10]. Jimenez-Ortega et al. [11] concluded that emotions induced by short emotionally valenced paragraphs are able to affect the processing of subsequent emotionally neutral sentence containing semantic or syntactic errors. In lexical decision tasks, participants responded faster to words with higher emotional valence as compared to neutral words or negatively valenced words [12]. In short, the effect of emotion is pervasive in experimental tasks.

### How music influences emotion

One of the more salient motivations to why people listen to music is music’s ability to induce a variety of emotions in listeners. The underlying mechanisms of how music is able to elicit emotions in people have been the focus of recent research, and the findings indicate that both psychoacoustic parameters and cultural factors are integrated the composition of the music and play a role in evoking emotions [13]. Several studies have shown that psychoacoustic parameters such as **tempo**, **mode** and **intensity**, amongst other parameters, are able to influence emotions [14–17]. Musical pieces are rated as ‘happier’ if they are in the major mode and have faster tempos and higher intensity. The opposite is true when pieces are in the minor mode and have slower tempos and lower intensities.

Several studies have found that certain cues such as mode and tempo have a stronger impact in evoking emotions in listeners [18,19]. Other studies indicate that tempo is more powerful in determining emotions for listeners in comparison to other features [20,21]. Tempo is found to be associated with the arousal dimension while mode is associated with the valence dimension [17,22,23]. This is because faster tempos may be associated with expressions of excitement and happiness, and express heightened arousal, while slower tempos may be associated with lower arousal levels [24]. However, fast and slow tempos may express either positive or negative valence. The major modes, in contrast, are associated with more pleasant emotions, while minor modes are associated more with unpleasant feelings. The present paper will focus on how mode and tempo interact with each other to influence emotions and in turn influence language use. In the next two sections, we discuss the parameters critical to reading research on music and emotion

### Experienced versus expressed emotions

Differences have been observed between emotions that are expressed by a musical piece and the emotions felt by the listener [25]. It is, therefore, important to make a distinction between the emotions that are experienced by a listener and the emotions that are expressed by the features of a musical piece. In Gabrielsson’s [26] schema there can be either a positive, negative or no relationship between the emotions perceived and the emotions expressed. A positive relationship means that the emotions felt are the same as the emotions expressed (e.g. hearing a sad piece of music and feeling sad). In contrast, a negative relationship would mean hearing a sad music piece but feeling happiness. It is also possible to find no relationship, where no emotions are felt [27]. They demonstrated how the participants’ ratings of emotion did not always coincide with perceived emotion of the music stimuli. Therefore, distinguishing between the emotions felt by the listener and emotions expressed by the music is paramount so that a clear separation of both types of responses is achieved.

### Cross cultural differences in emotion and music perception

As not all cultures may experience emotions in the same way, there may be cultural differences in the way music is perceived. For example, the feature of mode is a culturally-specific feature of music because it has only been found in Western tonal music. Mode is something that must be learnt through experience because children do not use the major/minor mode as a cue to distinguish emotions in music until they are about six to eight years of age [28].

It is well documented that certain basic emotions such as happiness, sadness and fear are recognized across many different cultures [29]. In contrast, a study on mood judgment differences between American and Chinese listeners found significantly higher ingroup agreement [30], indicating that there are culture specific differences in musical perception and emotion. Argstatter [31] and Wong, Roy and Margulis [32] reported similar findings with participants from Germany, Norway, South Korea, Indonesia and India. Lee and Hu [33] reported differences in emotion perception between American, Chinese and Korean participants, where participants from different cultures were more likely to select different mood clusters for the music they listened.

Intercultural studies of emotion have suggested that such cultural differences are due to the individualistic-collectivistic dimension. This extends to musical experiences as well where emotional properties of music may be linked to the specific emotional needs of the culture [34] and emotions that are more valued in a culture will be represented more frequently [35]. This was confirmed by Juslin et al. [36] in a study where participants from individualistic and collectivistic cultures completed a survey measuring musical emotions. In this study, emotions such as nostalgia and happiness were reported more frequently by participants from collectivistic cultures while sadness and melancholy were more likely to be found in individualistic cultures.

### The affective priming effect

We know affective priming through visual stimuli such as faces [37] and words [38] can affect language. The results from Carminati and Knoeferle’s [37] study demonstrated how priming with emotional expressions influences sentence processing of languages. In addition to visual stimuli, auditory stimuli such as music can also act as priming stimuli in an affective priming task.

According to Steinbeis and Koelsch’s [3] study, both musically trained and untrained participants evaluated emotion words more quickly when the preceding musical stimulus was of a similar affect to the word as compared to when a musical stimulus was of a different affect. For example, if the preceding musical stimulus was of a pleasant affect, such as having chords that sounded harmonious or in the major mode, participants were faster in evaluating their responses if the target word was also similar in affect, such as the word ‘love’, as compared to ‘hate’. This result is supported by other studies [2,39]. Likewise, March [40] found that music as affective prime also affected responses in semantic decision tasks where affectively congruent (music-word) pairs would result in faster reaction times as compared to when incongruent pairs were presented. These studies using auditory music primes focused more on semantic evaluation tasks and word evaluation tasks which tracked participants’ reaction times to the targets after exposure to the prime. They measure latent response, but how this shapes and influences behaviour when presented with a neutral target is not known. In addition, most of the studies mentioned above used individual notes and chords instead of melodies as their prime, and individual words as the target stimulus instead of passages. In this study, the purpose is to design a task that simulates what participants experience in a natural environment more closely.

## Methodology

The present study investigates whether affective priming effects would occur when music excerpts were used as an auditory prime and whether responses would be affected in a word selection task. This research is approved by the Ethic Review Committee of the Linguistics and Multilingual Studies, Nanyang Technological University. In contrast to previous studies, the present study uses music excerpts of 20 seconds, target stimuli of emotionally ambiguous pictures and selecting three emotion words from a list of 28 emotion words (S1 File). Therefore, instead of tracking reaction times to congruency, the present study aims to find out if music influences one’s feelings and if it also conditions one’s response to an object or situation. For example, if there are significant affective priming effects of music on language, participants will choose more positive valence and high arousal words when music pieces with faster tempos in the major key are played, compared to trials with music excerpts that are opposite in arousal and valence which are slow pieces in the minor mode.

Also, the majority of studies mentioned above were conducted in countries where Western tonal music is the norm or comparisons were made across groups that were monolingual. This study will provide insights to how emotions primed through music may influence word choice in Singapore’s context where the participants are either bilingual or multilingual, are exposed to different types of music and are collective in personality traits.

The study is a 2x3 within-subject design, with two independent variables–that is, the tempo of the music and the mode of the music pieces. There are two levels of manipulation for Mode (Major and Minor) and three levels of manipulation for Tempo (Fast, Original and Slow). The dependent variables are the total number of times the participants chose either positive or negative words and high arousal or low arousal words. The study only focused on analysing the use of positive and high arousal words and excluded the analysis of the negative and low arousal words because they are inversely related.

### Participants

The participants recruited for this experiment were 49 undergraduate and recent graduate students from universities in Singapore (24 males (*M*_*age*_ = 24.0 years, *SD* = 2.16) and 25 females (*M*_*age*_ = 23.5 years, *SD* = 1.87). Participation was voluntary and informed written consent was obtained from all individuals before the actual experimental session (S2 File). All participants completed a language background questionnaire to ascertain their bilingual or multilingual status. Only participants without formal music training were recruited for this study. Formal music training in this study is defined as having more than two years of experience playing an instrument and being enrolled in music lessons. In order to control for familiarity of music, participants were asked about the type of music they listened to and a majority of the participants listed pop music as the genre that they listen to most frequently. Only 26.5% of participants listed classical music as a genre they listen to, but even then, they were not familiar with the music excerpts used in this study. This means none of the participants would have strong emotional associations to the music excerpts played (S1 Dataset). All participants were bilinguals and knew at least two languages (S1 Dataset).

## Stimuli and measures

### Music stimuli

**Fig 2:**
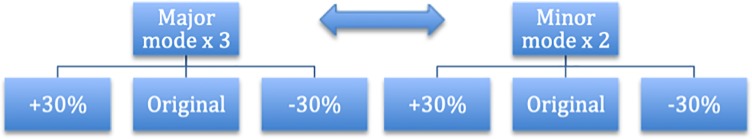
Diagram showing how music pieces will be transposed and how tempos will be manipulated.

Auditory priming was performed using five music pieces with three pieces in major mode and two in minor mode (S3 File). The music excerpts used were all piano pieces without lyrics. Each original music excerpt taken from the music piece was 20 seconds in length. Each piece was transposed to fit the other conditions where the major mode pieces were transposed to the minor mode, while the minor mode pieces were transposed to major mode. This resulted in ten music excerpts, five in major and five in minor mode (S4 File). The tempo of the music pieces was manipulated using the software Audacity Version 2.1.3 [41], producing a fast tempo track, which was 30% faster than the original piece, and a slow tempo track that was 30% slower than the original (Fig 2). The original excerpts were used as baseline trials to assess the effects of increased and decreased tempo on the emotion words selected by participants. Altogether there were 30 music excerpts in the experimental session.

**Fig 3:**
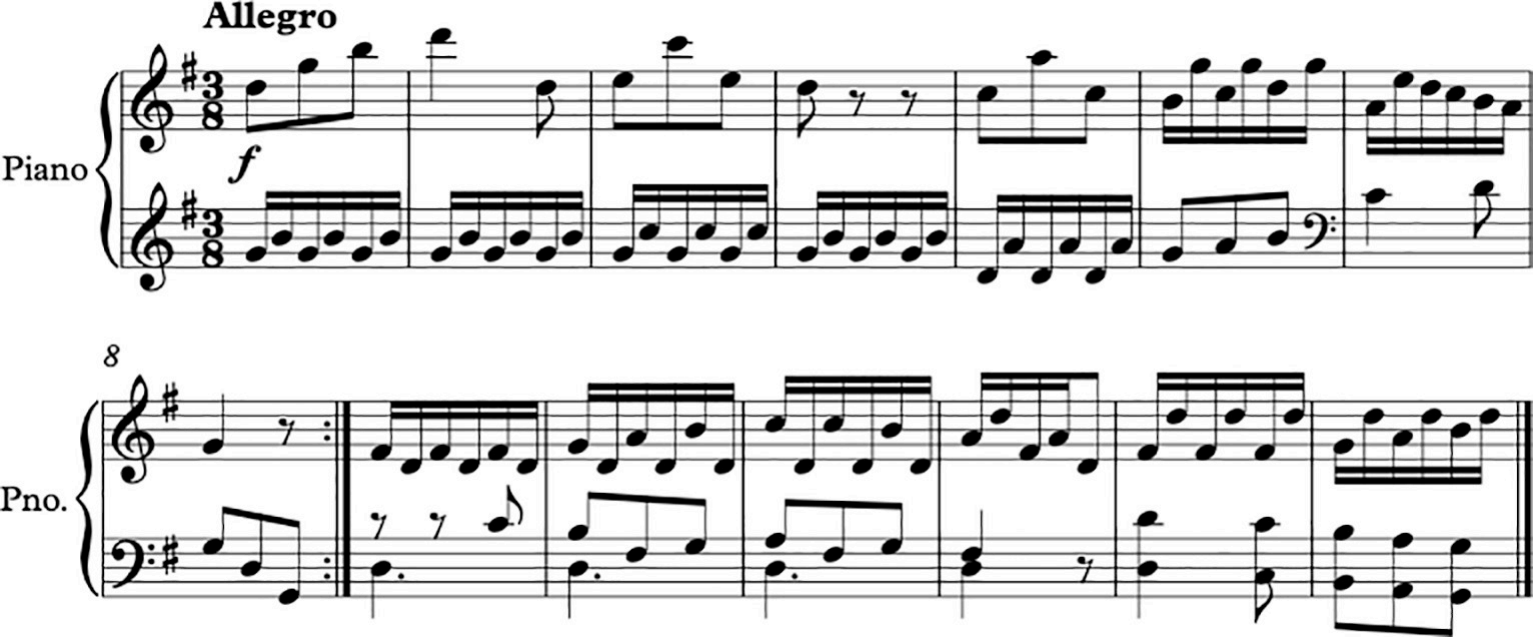
Excerpt of Haydn Hob 8. 4^th^ Movement- Original (Major key).

**Fig 4:**
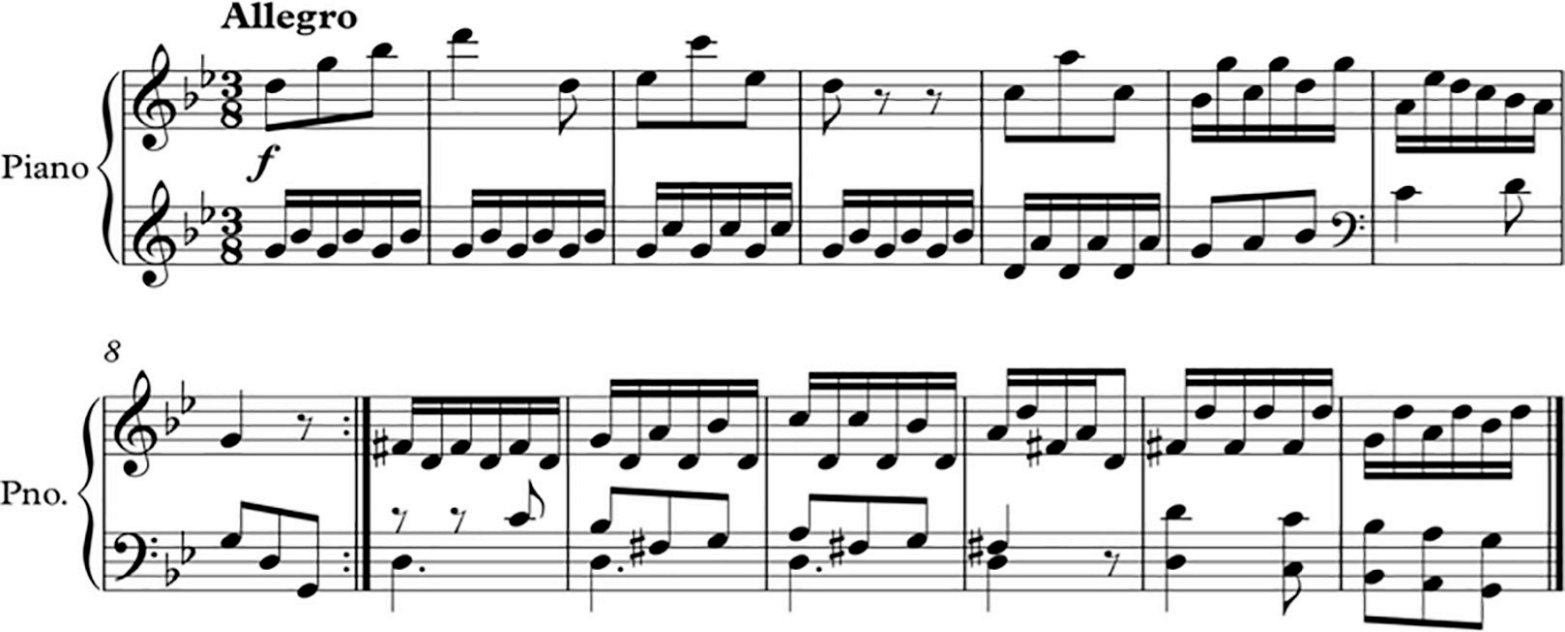
Excerpt of Haydn Hob 8. 4^th^ Movement—Altered (Minor key).

The score of one example of the music stimuli being transposed from major mode to minor mode is shown below (Figs 3 and 4).

There were 6 conditions: 1. Major mode–Fast tempo, 2. Major mode–Original tempo, 3. Major mode–Slow Tempo, 4. Minor mode–Fast Tempo, 5. Minor mode–Original tempo, 6. Minor–Slow tempo. In terms of valence and arousal, the major mode–fast tempo condition would be perceived as having the highest valence and highest arousal, while the minor mode–slow tempo condition would be the lowest valence and lowest arousal condition.

### Emotion word selection task

A list of 28 emotion words was compiled, and 30 pictures were selected from the Adobe Stock Library, where licenses were obtained for use of all the pictures and links to the pictures are provided (S5 File). The pictures were simple black and white line drawings. (Note: other normed pictures typically used in emotion studies such as IAPS (International Affective Pictures System) or NAPS (Nencki Affective Picture System) were not suitable as they were mainly utilised to elicit emotion state and the current study require stimuli that are neutral.) A group of 10 participants, five females and five males aged between 21 and 25 years (*M*_*age*_ = 23.0 years, *SD* = 1.58) who did not participate in the actual experimental session, rated the pictures on a scale of 1 = *Emotionally positive* to 7 = *Emotionally negative* (S6 File). The mean rating of the 30 pictures was 3.73 (*SD* = 0.34) (S2 Dataset). Participants rated most pictures as being emotionally neutral.

A pilot test was carried out with six participants who were not involved in the actual experiment. In the actual experimental session, there was a time limit of two minutes to choose three words and this timing was chosen after a pilot test was done. These words are placed on the circumplex model of affect [6]. Participants were also given the option to write any other words that they felt during the experimental session (S3 Dataset). After the data was collected, 13 individuals (*M*_*age*_ = 23.4 years, *SD* = 1.66) who did not take part in the pilot and the experiment rated the words on their valence and arousal (S4 Dataset). In addition, participants were also required to rate the intensity of the words that they had chosen on a scale of 1 = *Not intense at all* to 7 = *Extremely intense*.

### Demographic survey and music background

A basic demographic survey and a music background questionnaire were included in the survey and included questions such as the amount of musical training and participants’ music preferences (S1 File). Participants were asked to rate their mood before and at the end of the experiment and if they were able to recognise any of the music pieces.

### Procedure

The study adopted a within-subject design, and each participant was exposed to all six conditions. Each music condition consisted of five music excerpts. The experiments were conducted in quiet environments free of external distractions and loud noises, such as in an empty classroom or in a quiet corner in the library. The experiment was designed and presented on Qualtrics, an online survey platform. Participants wore Sennheiser PX 200-II over-ear headphones when listening to the music excerpts.

Before the experimental session, participants were required to rate how they felt and were provided with instructions on the procedure.

Two practice trials were conducted so that the participants could familiarise themselves with the words. The actual experimental session consisted of five blocks of six trials. To control for order effects such as carryover and practice effects, the five question blocks and trials in each block were randomised and presented in pseudo-random order. Participants were presented with 30 trials in total.

**Fig 5:**
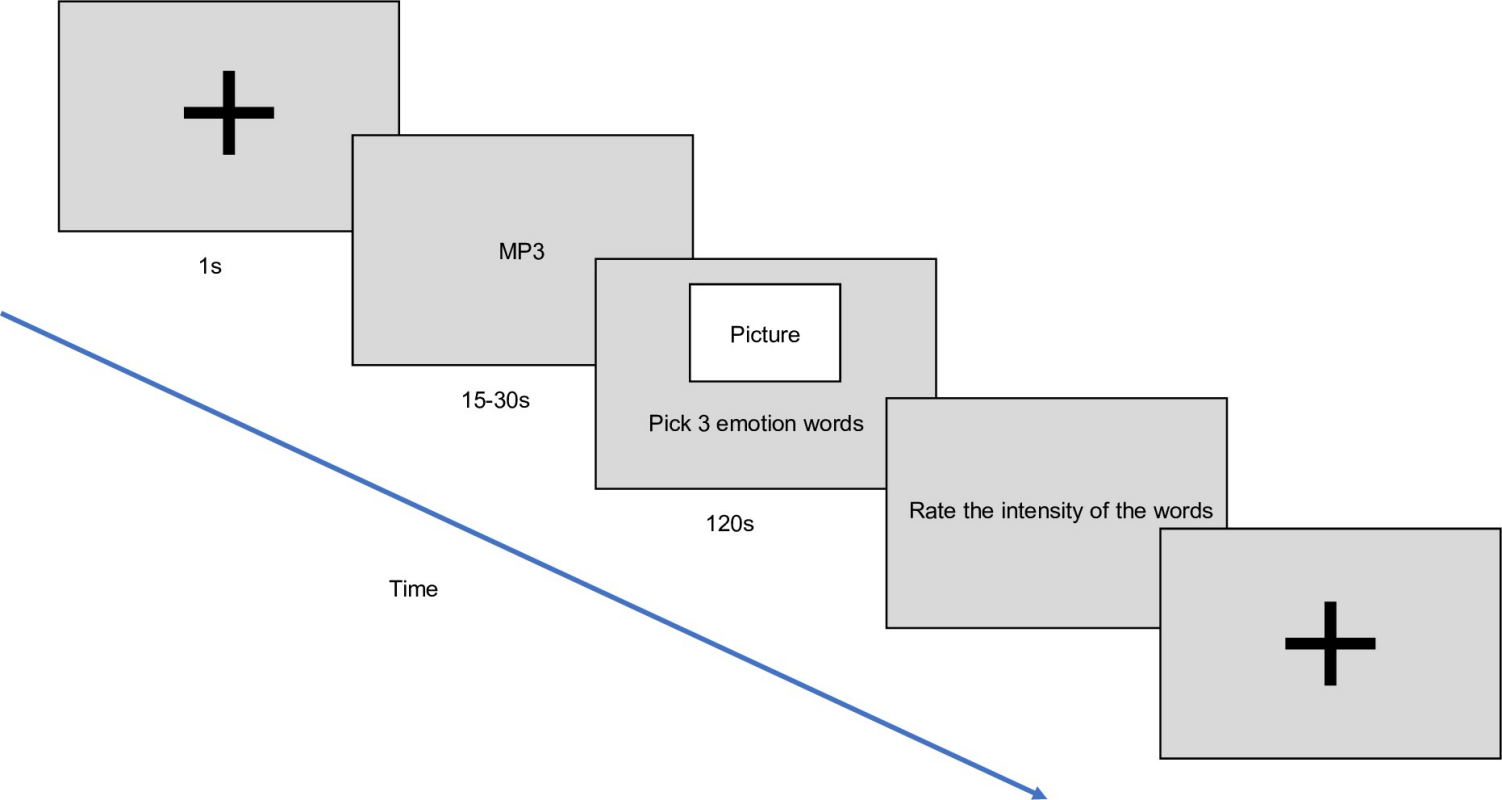
Visual illustration of the progression of each trial.

Fig 5 shows a schematic of the procedure. Before each trial, a fixation cross, accompanied by a beep, was presented for one second to fixate the participant’s gaze on the centre of the screen. Then, a music excerpt was played. After the music, a picture was shown on the screen together with the list of 28 emotion words. Participants were given two minutes to choose three words that best describe the scene. Participants were instructed to complete the task based on the emotions they experienced and not the emotions expressed in the music. Participants were also instructed to choose the most appropriate words as quickly as possible because there was a two-minute time limit. This was followed by another screen showing the three words they had chosen and the participants were asked to provide their perception of the intensity of the chosen words. No time limit was set for rating the intensities of the emotion words. Immediately after, the fixation cross, accompanied by a beep, was presented for one second to fixate their gaze on the centre of the screen, and the next trial would be presented. At the end of the experiment, the demographics survey and music listening questionnaire were presented to the participants (S1 File). Since participants were not required to use the full two minutes, and many participants took a shorter time to select their choices, each experimental session lasted approximately 30 to 45 minutes on average.

## Results

A repeated two-way analysis of variance (ANOVA) was conducted in SPSS with the number of times a positive or high arousal word was chosen as the dependent variable, while Tempo (fast, original, slow) and Mode (major and minor) were the two within-subject factors. To determine whether a relationship existed between the emotional state of participants before the experiment and the number of times positive or high arousal words selected, a Spearman rank-order correlation was carried out. The results of this test suggest that there were no significant correlations as shown in Tables 1 and 2.

**Table 1:** Spearman’s correlation coefficient of emotional state and positive words selected.

	Emotional State	
No. of positive words chosen in:	Correlation Coefficient	Sig (1-tailed)
Major Fast	-0.38	0.40
Major Original	0.18	0.11
Major Slow	-0.12	0.20
Minor Fast	-0.15	0.15
Minor Original	0.13	0.19
Minor Slow	-0.02	0.46

No. of positive words chosen in = Number of positive words chosen in the various music conditions, Emotional State = The emotional state of participants at the present time of the experiment

**Table 2:** Spearman’s correlation coefficients of emotional state and high arousal words selected.

	Emotional State	
No. of high arousal words chosen in:	Correlation Coefficient	Sig (1-tailed)
Major Fast	0.004	0.49
Major Original	-0.19	0.10
Major Slow	0.016	0.46
Minor Fast	0.14	0.16
Minor Original	-0.15	0.16
Minor Slow	0.022	0.44

No. of high arousal words chosen in = Number of high arousal words chosen in the various music conditions Emotional State = The emotional state of participants at the present time of the experiment

Since there are no significant correlations, this means that participants were not heavily influenced by their baseline moods before the start of the experiment and they chose and rated words similarly regardless of mood.

### Overall results

The results from the study found a significant main effect of both mode and tempo with regards to the number of times positive words (mode: (*F*(1,48) = 133.97, p<0.001, tempo: (*F*(2,96) = 3.229, p = 0.044, p < 0.05)) and high arousal words (mode: *F*(1,48) = 18.61, p <0.001, tempo: *F*(2,96) = 54.14, p <0.001)) were selected. The table below shows the means and standard deviations of the number of times a word was chosen and the music conditions.

The study also found that there were no interaction effects between mode and tempo (Mode x Tempo) in the number of positive words selected, but an interaction effect existed when Mode x Tempo was tested with the number of high arousal words selected (*F*(2,96) = 12.78, p <0.001). There was a significant effect of mode on the rated intensities on high arousal words (*F*(2,96) = 4.260, p < 0.05) and a positive correlation was also found between the number of high arousal words selected and the rated intensity of the word in the Major-Fast (Pearson’s *r*(47) = 0.440, n = 49, p = 0.001), Major-Slow (Pearson’s *r*(47) = 0.359, n = 49, p = 0.006 Pearson’s *r*(47) = 0.359, n = 49, p = 0.006) and Minor-Slow (Pearson’s *r*(47) = 0.414, n = 49, p = 0.002) condition.

In addition, the study found a difference between the categorization of word in the present study compared to the circumplex model, where positively valenced words and high level arousal words included *Happy*, *Delighted* and *Excited*, while *Tense* was the most commonly chosen word throughout, with the highest rated intensity.

### The effect of music on the number of times positive words were selected

#### Effect of mode

**Fig 6:**
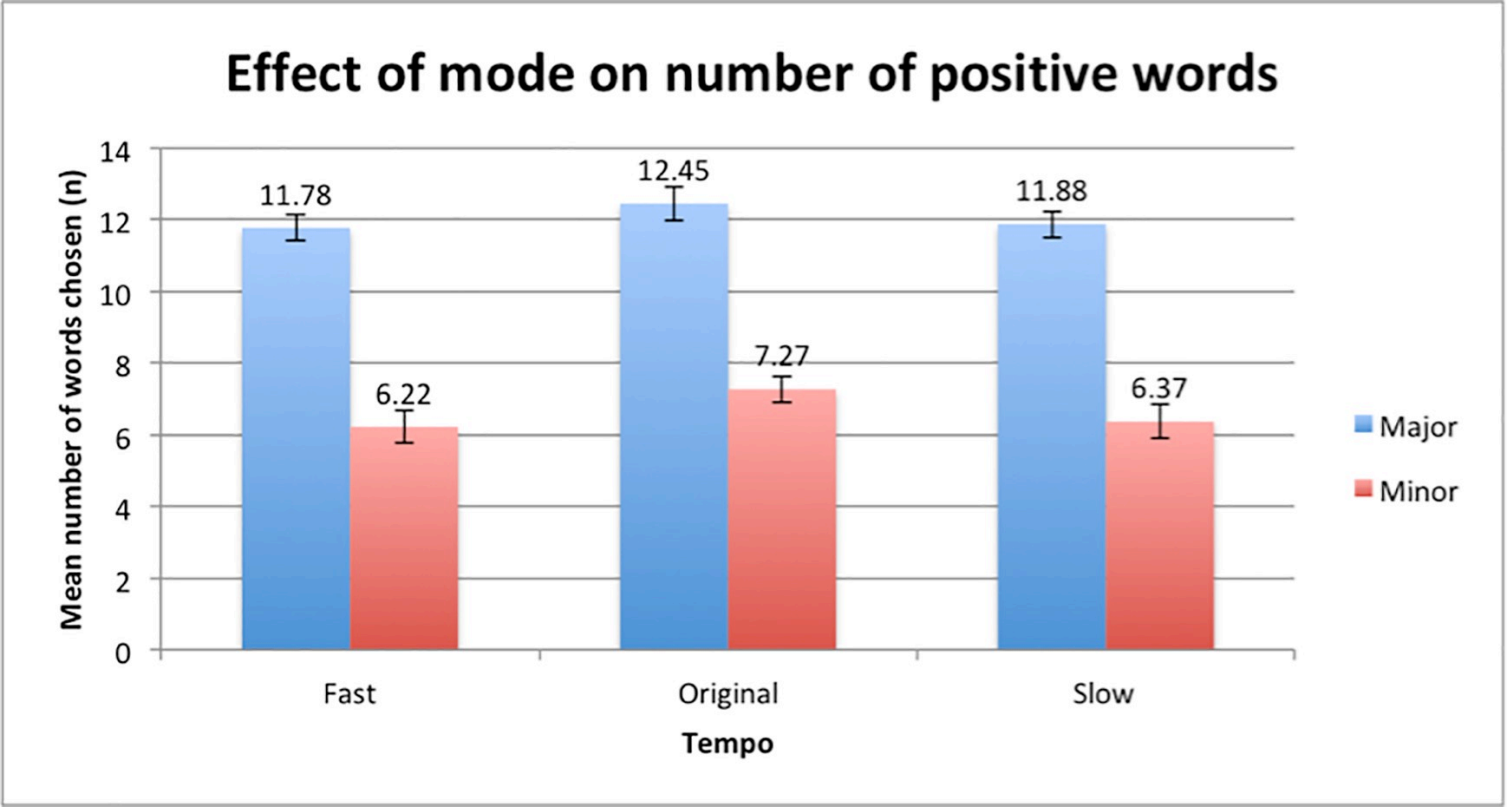
Differences in means of the selection of positive words between major and minor mode. Error bars indicate the standard error of the mean.

The number of positive words selected showed that there was a significant main effect of mode, *F*(1,48) = 133.97, p<0.001. The participants selected a higher number of positive words in the Major-Fast condition (*M* = 11.78, *SD* = 2.46), Major-Original condition (*M* = 12.45, *SD* = 2.62) and Major-Slow condition (*M* = 11.88, *SD* = 2.53) compared to the Minor-Fast condition (*M* = 6.22, *SD* = 3.28), Minor-Original condition (*M* = 7.27, *SD* = 3.26) and Minor-Slow condition (*M* = 6.37, *SD* = 3.37) respectively (Fig 6). The findings suggest that the major mode conditions elicited more positive attitudes and emotions compared to the minor mode conditions.

**Fig 7:**
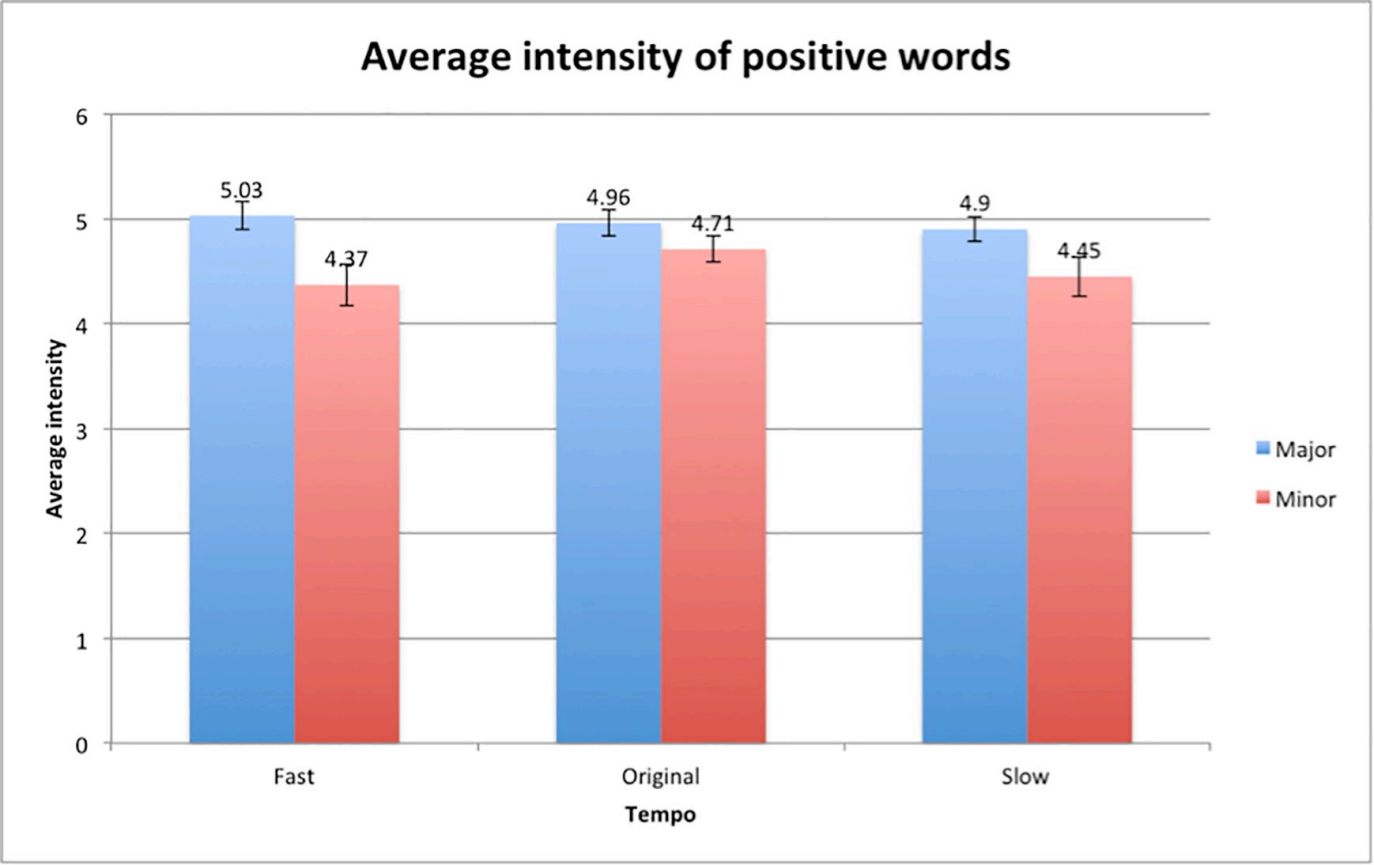
Average intensity rating of positive words in the major mode and minor mode conditions. Error bars indicate the standard error of the mean.

Further analysis of the intensity of the positive words selected revealed that there was a main effect of mode on the average intensities, *F*(1,48) = 23.291, p < 0.001, and the average intensities of the positive words selected was rated higher in the major mode (*M* = 4.96, *SD* = 0.06) compared to the minor mode conditions (*M* = 4.51, *SD* = 0.18). The average intensities of positive words (Fig 7) in the Major-Fast condition (*M* = 5.03, *SD* = 0.92) was higher compared to the Minor-Fast condition (*M* = 4.37, *SD* = 1.38), the Major-Original condition (*M* = 4.96, *SD* = 0.88) was higher compared to the Minor-Original condition (*M* = 4.71, *SD* = 0.88) and the Major-Slow condition (*M* = 4.90, *SD* = 0.80) elicited higher ratings of intensity compared to the Minor-Slow condition (*M* = 4.45, *SD* = 1.32). The finding suggests that valence and intensity are related, where positive words selected in the major mode conditions are more likely to be rated as more intense compared to the minor mode conditions.

#### Effect of tempo

**Fig 8:**
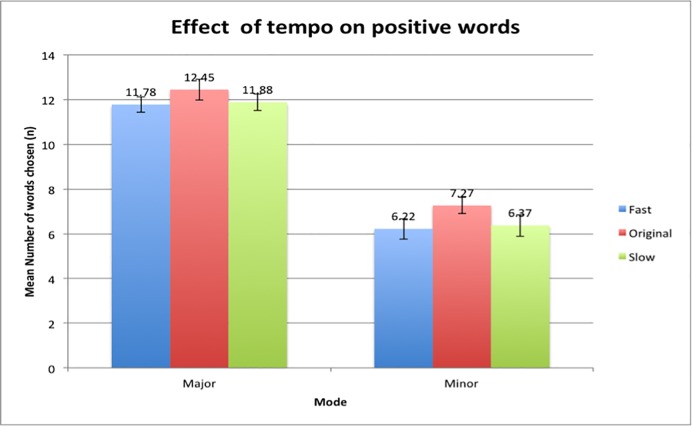
Mean number of positive words selected with respect to the various tempo conditions in both major and minor mode. Error bars indicate the standard error of the mean.

There was a main effect of tempo on the number of positive words selected where there was a significant difference between the responses of participants in the fast tempo, original tempo and slow tempo conditions, *F*(2,96) = 3.229, p = 0.044, p < 0.05. The data collected showed that the number of positive words (Fig 8) was highest in the original tempo conditions in both major and minor mode (*M* = 9.86, *SD* = 3.66), followed by the slow tempo condition (*M* = 9.12, *SD* = 3.90) and lastly the fast tempo condition (*M* = 9.00, *SD* = 3.93). The Major/Minor-Original pair elicited the highest means, followed by the Major/Minor-Slow and lastly the Major/Minor-Fast conditions. The participants were more likely to pick more positive words when music pieces were played in their original tempos compared to when the music was manipulated to be faster or slower. There were no main effects between the intensity of the positive words and the different tempo conditions (*F*(2,96) = 1.093, p = 0.34), indicating that participants across all tempo groups rated the intensities of positive words similarly.

#### Interaction effect between mode and tempo

There was no significant interaction effect between mode and tempo in the number of positive words selected, *F*(2,96) = 0.225, p = 0.80, p>0.05, and also no interaction effect for the intensity ratings of positive words, *F*(2,96) = 0.032, p = 0.21, p>0.05. This suggests that the effect of mode does not depend on tempo and that participants chose a similar number of positive words and rated those words similarly regardless when variables of mode and tempo were taken together.

This finding suggests that mode and tempo do play a role in affecting the valence dimension, where pieces in the major mode lead participants to choose more positive words compared to music pieces in the minor mode. Participants also rated positive words in the major condition as more intense than the positive words in the minor mode condition. However, contrary to expectation, more positive words were chosen when music pieces were in their original tempos compared to fast and slow tempos.

### The effect of music on the number of times high arousal words were selected

To investigate the effect of mode and tempo on the number of high arousal words selected, another repeated-measures ANOVA was conducted. This test revealed that there was both a significant main effect of mode, *F*(1,48) = 18.61, p <0.001 and tempo, *F*(2,96) = 54.14, p <0.001.

#### Effect of mode

**Fig 9:**
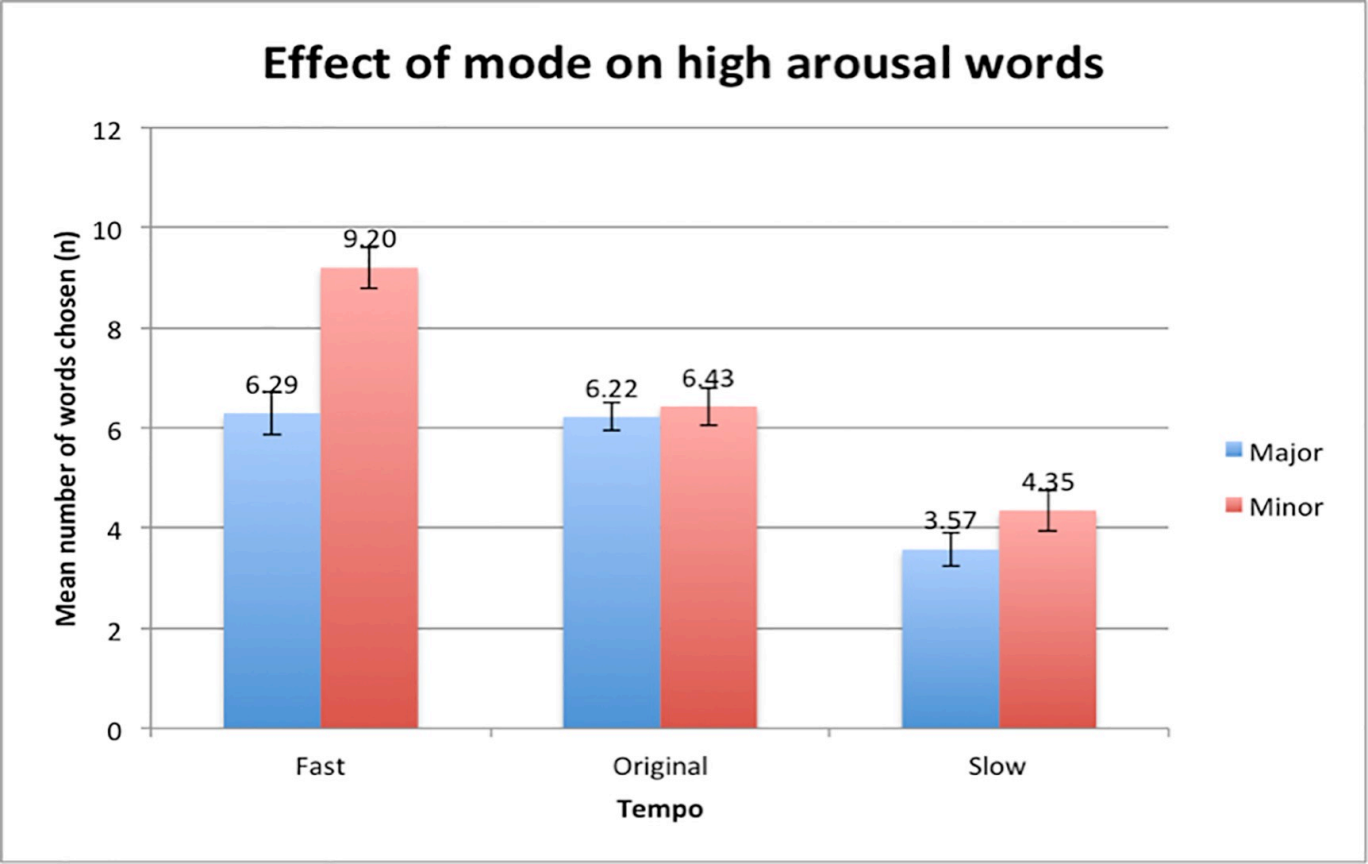
Differences in means of the selection of high arousal words between major and minor mode. Error bars indicate the standard error of the mean.

The mean differences of high arousal words selected between the major and minor mode was significant and higher in the minor mode condition (*M* = 6.66, *SD* = 2.43) compared to the major mode condition (*M* = 5.36, *SD* = 1.55). The results (Fig 9) also demonstrate that the Minor-Fast condition (*M* = 9.20, *SD* = 2.91) elicited more high arousal words as compared to the Major-Fast (*M* = 6.29, *SD* = 2.97) condition. This was consistent with the other pairs, with the Minor-Original (*M* = 6.43, *SD* = 2.64) and Minor-Slow (*M* = 4.35, *SD* = 2.84) conditions triggering a higher number of high arousal words compared to the Major-Original (*M* = 6.22, *SD* = 1.98) and the Major-Slow (*M* = 3.57, *SD* = 2.26) condition. This suggests that participants were more likely to feel stronger emotions and pick more words that were placed on the high arousal spectrum compared to music pieces in the major mode.

#### Effect of tempo

**Fig 10:**
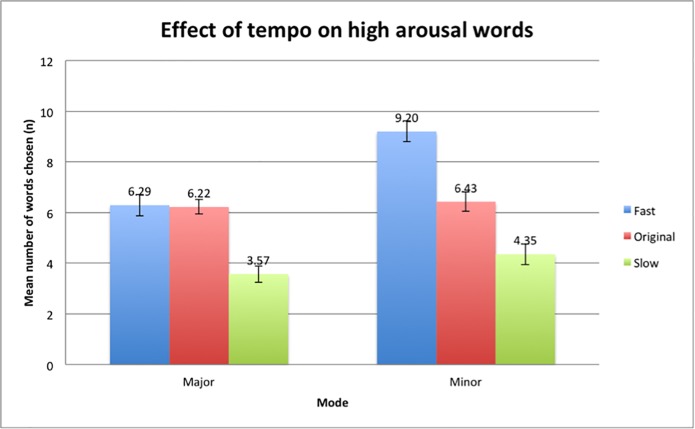
Differences in mean number of times high arousal words were selected, between the tempo conditions (Fast, Original, Slow). Error bars indicate the standard error of the mean.

In addition, the main effect of tempo was found to be significant where the mean number of high arousal words selected was higher in the fast tempo condition (*M* = 7.75, *SD* = 2.06) followed by the original tempo condition (*M* = 6.33, *SD* = 0.15) and then the slow tempo condition (*M* = 3.96, *SD* = 0.55) regardless of the mode of music (Fig 10). It is evident from the results that tempo plays an important role in the selection of high arousal words, where participants chose more high arousal words in fast tempo conditions compared to the other two conditions, suggesting that faster tempos of music can lead to heightened states of physiological activity.

#### Interaction effect between mode and tempo

**Fig 11:**
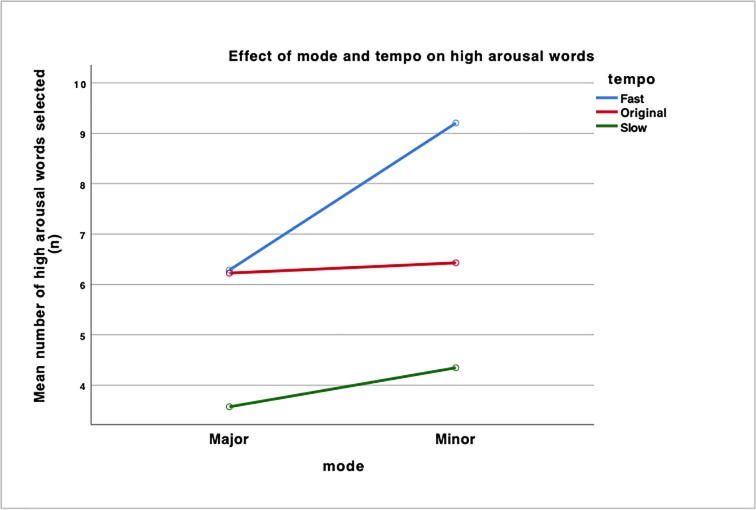
Interaction effect between the fast tempo and original tempo conditions on the mean number of high arousal words selected.

A Post-hoc Bonferroni correction analysis was done after conducting the repeated-measures ANOVA and found that an interaction effect exists, *F*(2,96) = 12.78, p <0.001) (Fig 11). This means that the variables of mode and tempo affect each other, where the difference between the fast tempo conditions and original tempo conditions was greater for the minor mode condition as compared to the major mode condition. More specifically, there was a significant difference in means in the Minor-Fast condition compared to the Major-Fast condition. The data implies that there may be an additive effect of mode and tempo where a music piece that is in both the minor mode and has a fast tempo will increase participants’ state of emotional arousal and this results in the selection of more high arousal words compared to the Major-Fast condition.

### The effect of music on the rated intensities of high arousal words

**Fig 12:**
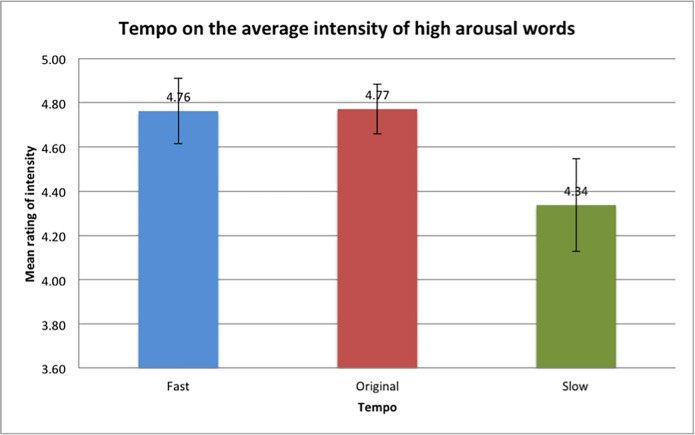
Average rated intensities of tempo on the high arousal words. Error bars indicate the standard error of the means.

In the circumplex model of affect, words of affect are placed in a circular dimension to categorise the emotions. However, even though participants may select a high arousal word with positive or negative valence, differences in arousal levels may exist within participants. Therefore, a two-way repeated measures ANOVA with the intensity ratings of high arousal words as the dependent variable and tempo and mode as the independent variables was conducted. No significant main effect of mode was found, *F*(1,48) = 2.206, p = 0.144. However, there was a significant main effect of tempo, *F*(2,96) = 4.260, p < 0.05, although there were no significant interaction effects found. The average intensity rating was also found to be highest in the original tempo conditions, followed by the fast tempo conditions and lastly the slow tempo conditions of both the major and minor mode (Fig 12). These findings imply that participants place more focus on the tempo of the music when rating intensity of the words. However, the means between the original and fast tempos differ very slightly, so it could be said that pieces in original and fast tempos elicit similar levels of emotional arousal but elicit higher levels of emotional arousal compared to slow tempos.

#### Relationships between the number and rated intensities of high arousal words

**Fig 13:**
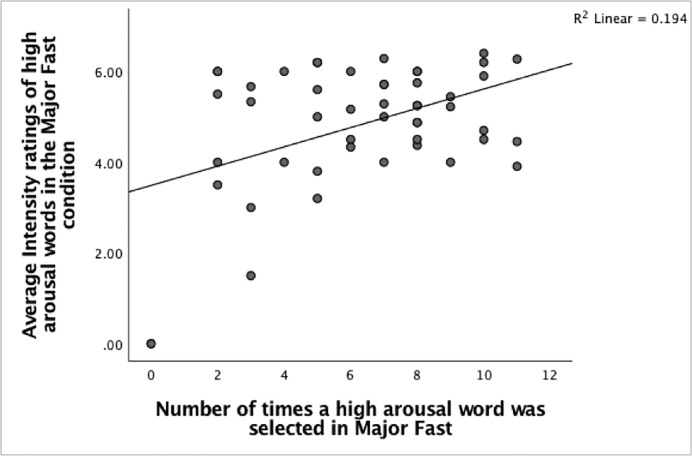
The relationship between the number of high arousal words selected and the rated intensities in the Major-Fast condition.

**Fig 14:**
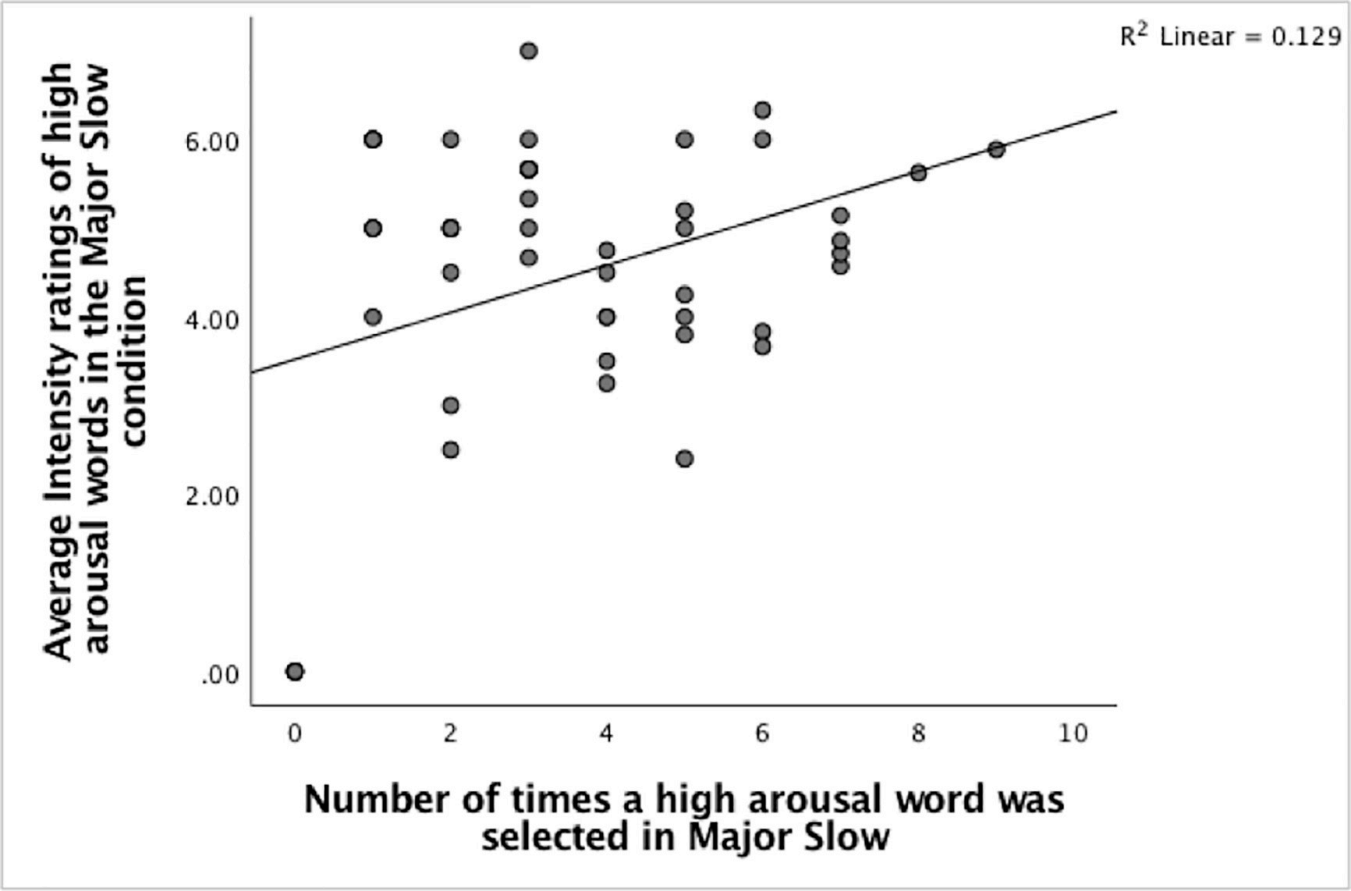
The relationship between the number of high arousal words selected and the rated intensities in the Major-Slow condition.

**Fig 15:**
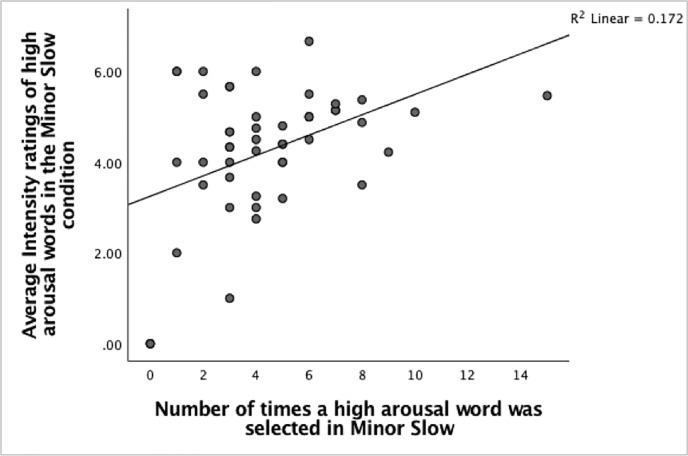
The relationship between the number of high arousal words selected and the rated intensities in the Minor-Slow condition.

Next, several Pearson product-moment correlation coefficients were carried out to examine if any relationships exist between the numbers of times high arousal words were selected and the average intensity of words selected in the different music conditions. The findings show significant moderate positive correlations exist between the number of times a high arousal word was chosen and the rated intensities in the Major-Fast condition, Pearson’s *r*(47) = 0.440, n = 49, p = 0.001, the Major-Slow condition, Pearson’s *r*(47) = 0.359, n = 49, p = 0.006 and the Minor-Slow condition, Pearson’s *r*(47) = 0.414, n = 49, p = 0.002. (Figs 13–15). The positive correlations suggest that when the number of times a high arousal word is chosen increases, the rated intensity also increases.

#### Examining the individual words chosen

Lastly, the average rated intensities of each high arousal word and the number of times the word was chosen was tabulated (Table 3) and results showed that there were differences between how the circumplex model of affect categorises words as compared to the present study. It can be seen that the highest rated and most selected positively valenced words include *Happy*, *Delighted* and *Excited* while for negatively valenced words, *Tense* was the most commonly chosen word throughout all the conditions with the highest rated intensity (S3 Dataset). Interestingly, Anger was an emotion that was chosen the least number of times throughout the conditions. This result reveals differences in how participants in the present study categorised words in the arousal dimension as compared to the circumplex model.

**Table 3:** Means and Standard deviations of words chosen in various music conditions.

	No. of times			
	positive words selected		high arousal words selected	
	M	SD	M	SD
Major Fast	11.78	2.46	6.29	2.97
Major Original	12.45	2.62	6.22	1.98
Major Slow	11.88	2.53	3.57	2.26
Minor Fast	6.22	3.28	9.20	2.91
Minor Original	7.27	3.26	6.43	2.64
Minor Slow	6.37	3.37	4.35	2.84

No. of times = Number of times a word was chosen, M = Mean, SD = Standard deviation.

Also, music excerpts in the minor mode were associated with more negative high arousal words compared to major mode pieces (Tables 4 and 5).

**Table 4:** The average intensity rating of each high arousal word (first column) and the number of times the word was chosen (second column) in the various music conditions.

	Music Condition											
Words	MajF		MajO		MajS		MinF		MinO		MinS	
***Positive***												
Aroused	4.67	***15***	4.22	***18***	4.22	***8***	4.58	***19***	4.59	***22***	4.86	***7***
Astonished	4.00	***7***	5.00	***5***	4.00	***4***	4.52	***22***	4.67	***5***	3.86	***7***
Excited	5.29	***65***	5.02	***62***	4.97	***29***	5.05	***56***	4.69	***48***	4.64	***14***
Delighted	5.42	***45***	5.02	***49***	4.92	***36***	4.76	***17***	4.80	***30***	4.45	***19***
Happy	5.28	***65***	4.92	***89***	4.92	***47***	4.75	***23***	4.63	***32***	4.70	***22***
***Negative***												
Alarmed	4.50	***12***	4.43	***7***	5.17	***6***	4.39	***38***	4.47	***17***	4.43	***7***
Tense	5.21	***39***	4.78	***21***	4.72	***18***	4.90	***95***	4.64	***56***	4.88	***34***
Afraid	4.00	***5***	5.75	***4***	4.67	***9***	4.60	***25***	3.93	***15***	4.80	***15***
Angry	0.00	***0***	3.33	***3***	7.00	***1***	4.80	***5***	4.00	***3***	3.50	***4***
Annoyed	4.67	***12***	4.11	***9***	4.86	***7***	4.68	***30***	4.79	***14***	4.47	***19***
Distressed	5.26	***19***	4.91	***11***	4.17	***6***	4.74	***56***	4.53	***31***	4.48	***21***
Frustrated	4.17	***12***	4.25	***8***	5.67	***3***	4.36	***38***	4.38	***20***	4.79	***28***

Words = The list of positive and negative words chosen, Music condition = the various music conditions that were included in the study including Major-Fast(MajF), Major-Original(MajO), Major-Slow(MajS), Minor-Fast(MinF), Minor-Original(MinO), Minor-Slow(MinS)

**Table 5:** The percentage of high arousal words with negative valence chosen in the minor mode.

Condition	Percentage of high arousal negatively valenced words
**Minor-Fast**	68.0%
**Minor-Original**	54.0%
**Minor-Slow**	64.0%

Condition = Music condition presented in the experiment in Minor mode and three levels of tempo

## Discussion

Overall, the results support the hypothesis that music pieces in the major mode are more likely to elicit positive words and that music pieces in a fast tempo induced a greater selection of high arousal words. However, fast tempo conditions did not elicit a higher number of positive words and, against expectations, more high-arousal words were selected when pieces were played in the minor mode as compared to the major mode pieces.

The present study shows that music pieces in the major mode are associated with more positive emotions while pieces in the minor mode are associated with more negative emotions and this is consistent with other studies [42,43]. According to Parncutt [44], this may be due to the theory of consonance and dissonance. This theory may indirectly explain the differences in the major keys and minor keys because consonant intervals are usually more harmonious, are seen as more pleasant and are characteristic of major chords. On the other hand, dissonant intervals usually cause feelings of tension and evoke unpleasant emotions, which are characteristic of minor chords. It has been suggested that humans prefer consonant chords and even infants as young as four months favour consonance over dissonance [45]. In an electroencephalography (EEG) study, consonant chords activated regions that regulate positive emotions, while dissonant chords activated regions of the right frontal lobe, which regulates negative emotions [46]. Hence, participants may have opted to select more positive words because major music is more pleasant to the ear.

Similar to other studies, tempo seems to play a significant role in affecting arousal [20]. Since tempo is related to the duration of time between beats, a faster tempo may be associated with faster motions and higher energies. A study which measured participants’ skin conductance and cardiovascular responses when listening to music, as well as their experienced arousal found that tempo may be related to physiological arousal [47]. The results from the study showed that participants rated faster tempos high on arousal and had higher scores on the skin conductance measure as compared to the slow tempos. Furthermore, altered tempos were also found to affect brain waves related to arousal, with beta wave amplitudes increasing with increased tempo [48]. Hence, it is conceivable that an increase in tempo is strongly related to an increase in physiological arousal. Consequently, this makes it more likely for participants to pick a greater number of high arousal words.

One other interesting finding from this study is that music pieces played at their original tempos elicited higher numbers of positive words compared to fast tempos and slow tempos even though faster tempos supposedly induce higher positive emotional valence [49]. One probable reason is because participants may have felt more uncomfortable listening to the tempo manipulated excerpts. Hence, the faster and slower tempos were seen as more negative compared to excerpts in their original state. This finding converges with work by Kim, Strohbach and Weddell [50] which found that liking ratings for songs decreased when tempo was increased or reduced.

Another possibility could be due to the similarities between speech and music. Evidence suggests that there is an overlap in the structural processing between the two domains [51,52]. Therefore, there may be a relationship between speech rates and music in affecting emotional responses. Bowling et al. [53] found that emotional expressions of vocal intonations in speech show parallels with trends in music, while other studies show that similar to tempo variations in music, differences in speech rates can affect responses to emotional categories. Fast speech rates were labelled negatively with emotions such as anger or fright while slow speech rates were labelled with sadness [54]. Furthermore, evidence also suggests that faster or slower speech rates affect personality perception and lead individuals to perceive the voice as being less benevolent, while normal speech rates were rated to be the most benevolent [55]. This pattern reflects the negative perception of faster or slower speech rates as compared to original, non-manipulated speech rates. Hence, the relationship between speech and music may be able to explain why unaltered tempos of music may sound more natural and may give rise to more positive words being selected.

The present study also found that music excerpts in the minor mode were associated with more negative high arousal words compared to major mode pieces. This finding is in line with studies that have found that minor modes have more negative connotations [56] and elicit higher arousal as compared to major modes [57]. Research in psychology has also found differences in responses to positive and negative stimuli. This could be attributed to the negativity bias, which is the bias of placing more emphasis on negative events [58], words and memories [59]. This emphasis has been found in several studies in which negative events tend to have a stronger physiological and emotional responses than positive or neutral events [60]. This is further substantiated in impression studies of personality in which negative attributes have more impact than positive traits [61,62]. Likewise, individuals are more likely to attend to negative emotions such as sadness and anger more intensely and for a longer period of time [63]. This may explain why more high arousal negative words were chosen in the minor mode conditions as compared to the major mode conditions. More specifically, there was a greater difference in the Minor-Fast condition compared to other conditions and one potential account could be due to additive effects of mode and tempo where the combination of minor mode and fast tempos resulted in an increase in the number of times a high arousal word of negative valence was chosen. These findings are consistent with the study by Ramos, Bueno and Bigand [64] who found that mode and tempo contribute independently towards emotional judgments.

As expected, original and fast tempos elicited higher average intensities of high arousal words compared to slow tempos. This further highlights the prominence of tempo as a factor affecting the arousal dimension. Additionally, positive correlations between the number of times a high arousal word was selected and rated intensity were found in the Major-Fast, Major-Slow and Minor-Slow conditions. Positive relationships would be expected because as the frequency of high arousal words chosen increases, it suggests that participants would feel heightened levels of arousal and thus respond with higher ratings of intensity for those words. These differences between the frequencies of high arousal words chosen could be explained by personality differences where particular traits such as extraversion and neuroticism can cause individuals to respond differently to emotional arousal. Studies have shown that being high in extraversion and neuroticism can lead to greater activations of regions of the right insula lobe and the right prefrontal cortex [65] and lead to stronger reactions of emotional arousal. Similarly, individuals high in agreeableness were found to experience stronger emotional reactions to affective stimuli [66]. This suggests that participants who possess these traits may be more inclined to choose more high arousal words and rate these words with greater intensities.

In addition to this, two out of the three conditions that had significant correlations were in the Major mode, which implies the importance of mode in determining this correlation. These findings are similar to research that shows that music pieces in the major mode evoke more intense positive feelings [57,67]. At the same time, our findings show that significant correlations were also found in the Minor-Slow condition. A more possible account could then be that high arousal words are felt more intensely at the extreme ends of the mode and tempo continuum of Major-Fast and Minor-Slow. The findings from Ladinig and Schellenberg’s [68] study revealed that consistent emotional cues of happiness (major mode, fast tempo) or sadness (minor mode, slow tempo) elicited more intense emotional responses compared to excerpts with conflicting cues such as with pieces in major mode, slow tempo. This is in line with the current study where the correlation for the Major-Slow condition was the weakest. This could be due to the cognitive attention mechanism, because evidence has shown that as attentional focus to an affective stimulus increases, the response received will be intensified [69]. Yet, attention is a limited capacity resource [70] and it is difficult to fully attend to all positive and negative aspects of an event. This results in less intense responses to ambiguous cues as compared to congruent affective cues. It is also important to note that the number of high arousal words selected in the minor condition was lower than those chosen in the major mode and were mostly negatively valanced. Hence, this could be a possible reason as to why positive relationships are found only in certain music conditions.

However, there is a distinct contrast between the circumplex model of affect and the results of this study. In the circumplex model (Fig 1) *Aroused* and *Astonished* have the highest ratings for intensity. In this study, *Happy*, *Delighted* and *Excited* had the highest ratings of intensity and *Anger* was the least chosen word throughout all music conditions. This inconsistency may be due to familiarity of emotions and cultural factors. Research has found that prototypical categories of positive emotions include joy and happiness [71] and in the English language, common words that imply ‘good feelings’ also include words such as joy, happiness, delight and excited [72]. These words also correspond with words most commonly used by young children [73,74] and also overlap with the examples that participants reported the most when asked to name emotion words [75]. This suggests positive emotions of happiness, delight and excitement are more common and familiar to participants. Given that research has found that people want and like to experience familiar emotions, especially for pleasant emotions [76], it is possible that participants chose more familiar words and rated them with greater intensity.

In the case of anger, it may be that cultural differences play a significant role because anger is a negative emotion and is displayed differently between cultures [77]. For example, anger as a norm violation signal has been found to differ in cultures [78] and in the Chinese culture, expressions of anger are not only less desirable [79], but also more covert. Overt displays of anger are seen negatively [80] and are avoided to minimise disruption of harmony. As Singapore, with 74% of its population ethnically Chinese, is still largely influenced by the Chinese culture, individuals in the present study may have been influenced by the cultural trait of having to avoid expressions of anger and chose not to select this word.

## Conclusion

This study demonstrated the enduring effect of music as an emotional prime that extends not just in subjective evaluation of words but also affects our worldview. It also showed that cultural factors play a role in how music affects the categorization of emotion words. These findings may be useful in the improvement of tools to help with emotional regulation. The results of this study could have implications in clinical settings where music priming could be used to facilitate appropriate emotional regulation and language use with people who have difficulties with communication, socializing with their peers or expressing their emotions. Music-based interventions have been in use for several years and have shown benefits with different groups of people [81,82]. The use of music may be beneficial with children with autism, as evidence has shown that music therapy produces a greater number and longer events of joy and positive responses such as initiation of engagement compared to just having toy play sessions [83]. Since initiation of social engagement is rare in children with autism, the clinical implications of such results may be significant in this field. Another group that may benefit are ‘at risk’ adolescents–evidence has shown significant pre- to post- programme improvements in emotional awareness when a music therapy programme was administered [84]. Music intervention has been shown to decrease symptoms of dementia in the elderly even after intervention ends [85] and is able to decrease anxiety levels and increase social behaviours [86]. In general, the more we understand how music and language affects emotion, the more we will be able to use music as a therapy in different settings, the effect of which has been known to be enduring and long-lasting [87].

## Supporting information

S1 File Complete questionnaire of the experiment. (PDF)

S2 File Consent form to participants. (PDF)

S3 File Names of music excerpts used in this study. (PDF)

S4 File Audios of music excerpts used in this study. (ZIP)

S5 File Links to images used in testing. (PDF)

S6 File Survey on the emotional valence of the pictures used in the study. (PDF)

S1 Dataset Data of the experiment. (ZIP)

S2 Dataset Data of individual words chosen. (XLSX)

S3 Dataset Ratings of ‘other’ words. (XLS)

S4 Dataset Results on the emotional valence of the pictures used in the study. (XLS)
