# Correction: Effects of affective priming through music on the use of emotion words

**DOI:** 10.1371/journal.pone.0222965

**Published:** 2019-09-18

**Authors:** Rosabel Yu Ling Tay, Bee Chin Ng

In the Funding section, the AcRF Tier 1 grant number is listed incorrectly. The correct grant number is: M4011741.

## References

[1] Tay RYL, Ng BC (2019) Effects of affective priming through music on the use of emotion words. PLoS ONE 14(4): e0214482 10.1371/journal.pone.0214482 30990819 PMC6467386

