# Supplementary material for: Effects of affective priming through music on the use of emotion words
Source: PLoS One. 2019 Apr 16;14(4):e0214482. doi: 10.1371/journal.pone.0214482 (PMC6467386; doi:10.1371/journal.pone.0214482)
Supplement: S1 File — (PDF) [file pone.0214482.s001.pdf]

Consent form

Title of Project: The affective effects of music on language use
Principal Investigator/Researcher: Tay Yu Ling Rosabel

Purpose of the Study
The purpose of this study is to investigate the effects of music on use of emotion words in listeners. It aims to understand the relationship between music, emotion and language, while assessing the potential advantages of music as a tool to better understand emotions.

Procedure of the Study
As a participant in this study, you will be asked to do this experiment in a quiet and convenient location. During the experiment, you will be required to listen to some music excerpts and complete a word choice task right after each hearing each music excerpt. You will also be asked to complete a demographics survey and a music listening questionnaire about your music listening preferences and music experience. This experiment will take approximately 30 minutes to complete.

Minimisation of Risk
Taking part in this study involves minimal risk such as eye discomfort and fatigue. However, there will be plenty of opportunities for breaks during the experiment. Nevertheless, should you feel uncomfortable in any way or wish to withdraw from the study, please let the researcher know.
The experiment in this study is not designed to be of an especially sensitive or personal nature and all information provided will be kept in strict confidence. All data collected will be stored in a password encrypted electronic file.

Benefits of the Study
While you may not experience any direct benefits of the study, the findings of this study will further our understanding on music, emotion and language and may provide insights to improve therapy services especially for groups who have difficulties in expressing their emotions.

Confidentiality
All information provided by you in the course of completing this experiment will be kept in strict confidence and securely stored to the extent allowed by law. Any data and results reported in academic papers and presentations will be made anonymous.

Participation and Withdrawal
Your participation is voluntary. You can refuse to participate, or withdraw from the study at any time without any negative consequences.

Contact Details
If you have any questions or concerns about this research, please feel free to contact me Tay Yu Ling Rosabel at TAYY0055@e.ntu.edu.sg, Telephone: 93211018.
If you have any questions concerning research ethics and your rights as a research participant, please contact my supervisor, Associate Professor Ng Bee Chin (Address: Division of Linguistics and Multilingual Studies, HSS, 14 Nanyang Drive, Nanyang Technological University, Singapore; Telephone: (65) 6592 3238; Email: MBCNG@NTU.EDU.SG).

By clicking the button below to proceed with the experiment, You,

Confirm that you have read and understood the information sheet for the above study and have had the opportunity to ask questions.

i. Understand that your participation is voluntary and that you are free to withdraw at any time, without giving any reason, without your legal rights being affected.

ii. Understand that sections of any of your input in this study may be looked at by responsible individuals from the Nanyang Technological University or regulatory authorities where it is relevant to your taking part in research. You give permission for these individuals to have access to your input.

iii. Agree to take part in the above study.

Instructions to participants

You will be required to listen to several music excerpts. Please **focus on the emotions that you felt** while listening to the music, and not the emotions expressed by the music piece.

After listening to the music, a picture will be shown to you and you will given 2 minutes to answer a few questions regarding the picture.

You will be given **2** practice trials before the start of the real experimental session.

Practice trials

Please rate your mood at the current moment

- Extremely happy
- Moderately happy
- Slightly happy
- Neutral
- Slightly unhappy
- Moderately unhappy
- Extremely unhappy

Please listen to the following mp3s and answer the questions that follow.

There will be a time limit on the questions so please complete the questions as soon as possible!

Please press the next button to continue to the practice trials.

Practice trials

+

These page timer metrics will not be displayed to the recipient.

First Click: *0 seconds*

Last Click: *0 seconds*

Page Submit: *0 seconds*

Click Count: *0 clicks*

MP3: ▶ -00:00

What do you feel when you look at this picture? [Pick 3]

☐ Pleased	☐ Relaxed	☐ Frustrated	☐ Distressed	☐ Astonished	☐ At ease
☐ Sad	☐ Serene	☐ Satisfied	☐ Depressed	☐ Aroused	☐ Glad
☐ Alarmed	☐ Sleepy	☐ Tired	☐ Delighted	☐ Gloomy	☐ Tense
☐ Happy	☐ Excited	☐ Calm	☐ Content	☐ Bored	☐ Others ____
☐ Miserable	☐ Annoyed	☐ Afraid	☐ Droopy	☐ Angry	

Rate the intensity of the emotions you have chosen

	Not intense at all					Extremely intense
	1	2	3	4	5	6	7
» Pleased							
» Sad							
» Alarmed							
» Happy							

» Miserable

» Relaxed

» Serene

» Sleepy

» Excited

» Annoyed

» Frustrated

» Satisfied

» Tired

» Calm

» Afraid

» Distressed

» Depressed

» Delighted

» Content

» Droopy

» Astonished

» Aroused

» Gloomy

» Bored						
» Angry						
» At ease						
» Glad						
» Tense						
» Others						

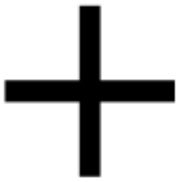

These page timer metrics will not be displayed to the recipient.

First Click: *0 seconds*

Last Click: *0 seconds*

Page Submit: *0 seconds*

Click Count: *0 clicks*

MP3 : -0:13

What do you feel when you look at this picture? [Pick 3]

☐ Pleased	☐ Relaxed	☐ Frustrated	☐ Distressed	☐ Astonished	☐ At ease
☐ Sad	☐ Serene	☐ Satisfied	☐ Depressed	☐ Aroused	☐ Glad
☐ Alarmed	☐ Sleepy	☐ Tired	☐ Delighted	☐ Gloomy	☐ Tense
☐ Happy	☐ Excited	☐ Calm	☐ Content	☐ Bored	☐ Others

☐ Miserable ☐ Annoyed ☐ Afraid ☐ Droopy ☐ Angry

Rate the intensity of the emotions you have chosen

	Not intense at all						Extremely intense
	1	2	3	4	5	6	7
» Pleased							
» Sad							
» Alarmed							
» Happy							
» Miserable							
» Relaxed							
» Serene							
» Sleepy							
» Excited							
» Annoyed							
» Frustrated							
» Satisfied							
» Tired							
» Calm							
» Afraid							
» Distressed							

» Depressed

» Delighted

» Content

» Droopy

» Astonished

» Aroused

» Gloomy

» Bored

» Angry

» At ease

» Glad

» Tense

» Others

END OF PRACTICE TRIAL

PRESS THE NEXT BUTTON TO PROCEED TO THE ACTUAL EXPERIMENTAL SESSION!

Actual experiment - Block 1

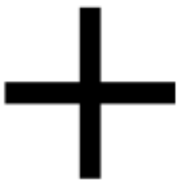

These page timer metrics will not be displayed to the recipient.
First Click: *0 seconds*
Last Click: *0 seconds*
Page Submit: *0 seconds*
Click Count: *0 clicks*

MP3: ▶ -0:18

These page timer metrics will not be displayed to the recipient.
First Click: *0 seconds*
Last Click: *0 seconds*
Page Submit: *0 seconds*
Click Count: *0 clicks*

0159

After listening to the music, what do you feel when you look at this picture? [Pick 3]

☐ Pleased	☐ Relaxed	☐ Frustrated	☐ Distressed	☐ Astonished	☐ At ease
☐ Sad	☐ Serene	☐ Satisfied	☐ Depressed	☐ Aroused	☐ Glad
☐ Alarmed	☐ Sleepy	☐ Tired	☐ Delighted	☐ Gloomy	☐ Tense
☐ Happy	☐ Excited	☐ Calm	☐ Content	☐ Bored	☐ Others ____
☐ Miserable	☐ Annoyed	☐ Afraid	☐ Droopy	☐ Angry	

Rate the intensity of the emotions you have chosen

Not intense at all						Extremely intense
1	2	3	4	5	6	7

» Pleased

» Sad

» Alarmed

» Happy

» Miserable

» Relaxed

» Serene

» Sleepy

» Excited

» Annoyed

» Frustrated

» Satisfied

» Tired

» Calm

» Afraid

» Distressed

» Depressed

» Delighted

» Content

» Droopy	
» Astonished	
» Aroused	
» Gloomy	
» Bored	
» Angry	
» At ease	
» Glad	
» Tense	
» Others	

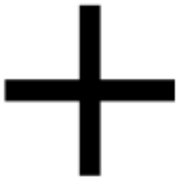

These page timer metrics will not be displayed to the recipient.

First Click: *0 seconds*

Last Click: *0 seconds*

Page Submit: *0 seconds*

Click Count: *0 clicks*

MP3: -0:20

These page timer metrics will not be displayed to the recipient.

First Click: *0 seconds*

Last Click: *0 seconds*

Page Submit: *0 seconds*

Click Count: *0 clicks*

0159

After listening to the music, what do you feel when you look at this picture? [Pick 3]

☐ Pleased	☐ Relaxed	☐ Frustrated	☐ Distressed	☐ Astonished	☐ At ease
☐ Sad	☐ Serene	☐ Satisfied	☐ Depressed	☐ Aroused	☐ Glad
☐ Alarmed	☐ Sleepy	☐ Tired	☐ Delighted	☐ Gloomy	☐ Tense
☐ Happy	☐ Excited	☐ Calm	☐ Content	☐ Bored	☐ Others ______
☐ Miserable	☐ Annoyed	☐ Afraid	☐ Droopy	☐ Angry	

Rate the intensity of the emotions you have chosen

	Not intense at all						Extremely intense
	1	2	3	4	5	6	7
» Pleased							
» Sad							
» Alarmed							
» Happy							
» Miserable							
» Relaxed							
» Serene							
» Sleepy							

» Excited						
» Annoyed						
» Frustrated						
» Satisfied						
» Tired						
» Calm						
» Afraid						
» Distressed						
» Depressed						
» Delighted						
» Content						
» Droopy						
» Astonished						
» Aroused						
» Gloomy						
» Bored						
» Angry						
» At ease						
» Glad						

» Tense	
» Others	

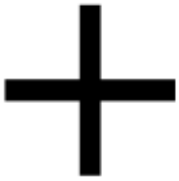

These page timer metrics will not be displayed to the recipient.

First Click: *0 seconds*

Last Click: *0 seconds*

Page Submit: *0 seconds*

Click Count: *0 clicks*

MP3: -0:28

These page timer metrics will not be displayed to the recipient.

First Click: *0 seconds*

Last Click: *0 seconds*

Page Submit: *0 seconds*

Click Count: *0 clicks*

01 59

After listening to the music, what do you feel when you look at this picture? [Pick 3]

☐ Pleased	☐ Relaxed	☐ Frustrated	☐ Distressed	☐ Astonished	☐ At ease
☐ Sad	☐ Serene	☐ Satisfied	☐ Depressed	☐ Aroused	☐ Glad
☐ Alarmed	☐ Sleepy	☐ Tired	☐ Delighted	☐ Gloomy	☐ Tense
☐ Happy	☐ Excited	☐ Calm	☐ Content	☐ Bored	☐ Others ____
☐ Miserable	☐ Annoyed	☐ Afraid	☐ Droopy	☐ Angry	

Rate the intensity of the emotions you have chosen

Not intense at all — Extremely intense

	1	2	3	4	5	6	7
» Pleased							
» Sad							
» Alarmed							
» Happy							
» Miserable							
» Relaxed							
» Serene							
» Sleepy							
» Excited							
» Annoyed							
» Frustrated							
» Satisfied							
» Tired							
» Calm							
» Afraid							
» Distressed							

» Depressed

» Delighted

» Content

» Droopy

» Astonished

» Aroused

» Gloomy

» Bored

» Angry

» At ease

» Glad

» Tense

» Others

These page timer metrics will not be displayed to the recipient.

First Click: *0 seconds*

Last Click: *0 seconds*

Page Submit: *0 seconds*

Click Count: *0 clicks*

MP3: ▶ -0:20

These page timer metrics will not be displayed to the recipient.

First Click: *0 seconds*

Last Click: *0 seconds*

Page Submit: *0 seconds*

Click Count: *0 clicks*

01 59

After listening to the music, what do you feel when you look at this picture? [Pick 3]

☐ Pleased	☐ Relaxed	☐ Frustrated	☐ Distressed	☐ Astonished	☐ At ease
☐ Sad	☐ Serene	☐ Satisfied	☐ Depressed	☐ Aroused	☐ Glad
☐ Alarmed	☐ Sleepy	☐ Tired	☐ Delighted	☐ Gloomy	☐ Tense
☐ Happy	☐ Excited	☐ Calm	☐ Content	☐ Bored	☐ Others
☐ Miserable	☐ Annoyed	☐ Afraid	☐ Droopy	☐ Angry	

Rate the intensity of the emotions you have chosen

	Not intense at all						Extremely intense
	1	2	3	4	5	6	7
» Pleased							
» Sad							
» Alarmed							
» Happy							
» Miserable							

» Relaxed
» Serene
» Sleepy
» Excited
» Annoyed
» Frustrated
» Satisfied
» Tired
» Calm
» Afraid
» Distressed
» Depressed
» Delighted
» Content
» Droopy
» Astonished
» Aroused
» Gloomy
» Bored

» Angry						
» At ease						
» Glad						
» Tense						
» Others						

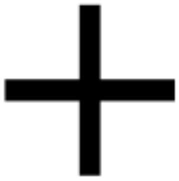

These page timer metrics will not be displayed to the recipient.

First Click: *0 seconds*

Last Click: *0 seconds*

Page Submit: *0 seconds*

Click Count: *0 clicks*

MP3: -0:20

These page timer metrics will not be displayed to the recipient.

First Click: *0 seconds*

Last Click: *0 seconds*

Page Submit: *0 seconds*

Click Count: *0 clicks*

01 59

After listening to the music,what do you feel when you look at this picture? [Pick 3]

☐ Pleased	☐ Relaxed	☐ Frustrated	☐ Distressed	☐ Astonished	☐ At ease
☐ Sad	☐ Serene	☐ Satisfied	☐ Depressed	☐ Aroused	☐ Glad
☐ Alarmed	☐ Sleepy	☐ Tired	☐ Delighted	☐ Gloomy	☐ Tense
☐ Happy	☐ Excited	☐ Calm	☐ Content	☐ Bored	☐ Others ______
☐ Miserable	☐ Annoyed	☐ Afraid	☐ Droopy	☐ Angry	

Rate the intensity of the emotions you have chosen

	Not intense at all					Extremely intense
	1	2	3	4	5	6	7
» Pleased							
» Sad							
» Alarmed							
» Happy							
» Miserable							
» Relaxed							
» Serene							
» Sleepy							
» Excited							
» Annoyed							
» Frustrated							
» Satisfied							
» Tired							

» Calm						
» Afraid						
» Distressed						
» Depressed						
» Delighted						
» Content						
» Droopy						
» Astonished						
» Aroused						
» Gloomy						
» Bored						
» Angry						
» At ease						
» Glad						
» Tense						
» Others						

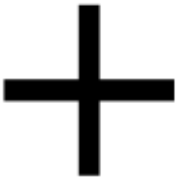

These page timer metrics will not be displayed to the recipient.
First Click: *0 seconds*
Last Click: *0 seconds*
Page Submit: *0 seconds*
Click Count: *0 clicks*

MP3: -0:19

These page timer metrics will not be displayed to the recipient.
First Click: *0 seconds*
Last Click: *0 seconds*
Page Submit: *0 seconds*
Click Count: *0 clicks*

01 59

After listening to the music, what do you feel when you look at this picture? [Pick 3]

☐ Pleased	☐ Relaxed	☐ Frustrated	☐ Distressed	☐ Astonished	☐ At ease
☐ Sad	☐ Serene	☐ Satisfied	☐ Depressed	☐ Aroused	☐ Glad
☐ Alarmed	☐ Sleepy	☐ Tired	☐ Delighted	☐ Gloomy	☐ Tense
☐ Happy	☐ Excited	☐ Calm	☐ Content	☐ Bored	☐ Others ______
☐ Miserable	☐ Annoyed	☐ Afraid	☐ Droopy	☐ Angry	

Rate the intensity of the emotions you have chosen

Not intense at all						Extremely intense
1	2	3	4	5	6	7

» Pleased	
» Sad	
» Alarmed	
» Happy	
» Miserable	
» Relaxed	
» Serene	
» Sleepy	
» Excited	
» Annoyed	
» Frustrated	
» Satisfied	
» Tired	
» Calm	
» Afraid	
» Distressed	
» Depressed	
» Delighted	
» Content	

» Droopy

» Astonished

» Aroused

» Gloomy

» Bored

» Angry

» At ease

» Glad

» Tense

» Others

PROCEED TO THE NEXT BLOCK

Actual experiment - Block 2

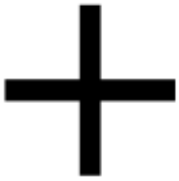

These page timer metrics will not be displayed to the recipient.

First Click: *0 seconds*

Last Click: *0 seconds*
Page Submit: *0 seconds*
Click Count: *0 clicks*

MP3: ▶ -0:15

These page timer metrics will not be displayed to the recipient.**

These page timer metrics will not be displayed to the recipient.
First Click: *0 seconds*
Last Click: *0 seconds*
Page Submit: *0 seconds*
Click Count: *0 clicks*

01 59

After listening to the music, what do you feel when you look at this picture? [Pick 3]

☐ Pleased	☐ Relaxed	☐ Frustrated	☐ Distressed	☐ Astonished	☐ At ease
☐ Sad	☐ Serene	☐ Satisfied	☐ Depressed	☐ Aroused	☐ Glad
☐ Alarmed	☐ Sleepy	☐ Tired	☐ Delighted	☐ Gloomy	☐ Tense
☐ Happy	☐ Excited	☐ Calm	☐ Content	☐ Bored	☐ Others ____
☐ Miserable	☐ Annoyed	☐ Afraid	☐ Droopy	☐ Angry	

Rate the intensity of the emotions you have chosen

	Not intense at all						Extremely intense
	1	2	3	4	5	6	7
» Pleased							
» Sad							
» Alarmed							
» Happy							
» Miserable							

» Relaxed

» Serene

» Sleepy

» Excited

» Annoyed

» Frustrated

» Satisfied

» Tired

» Calm

» Afraid

» Distressed

» Depressed

» Delighted

» Content

» Droopy

» Astonished

» Aroused

» Gloomy

» Bored

» Angry	
» At ease	
» Glad	
» Tense	
» Others	

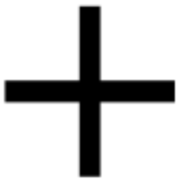

These page timer metrics will not be displayed to the recipient.
First Click: *0 seconds*
Last Click: *0 seconds*
Page Submit: *0 seconds*
Click Count: *0 clicks*

MP3: -0:20

These page timer metrics will not be displayed to the recipient.
First Click: *0 seconds*
Last Click: *0 seconds*
Page Submit: *0 seconds*
Click Count: *0 clicks*

01 59

After listening to the music, what do you feel when you look at this picture? [Pick 3]

☐ Pleased	☐ Relaxed	☐ Frustrated	☐ Distressed	☐ Astonished	☐ At ease
☐ Sad	☐ Serene	☐ Satisfied	☐ Depressed	☐ Aroused	☐ Glad
☐ Alarmed	☐ Sleepy	☐ Tired	☐ Delighted	☐ Gloomy	☐ Tense
☐ Happy	☐ Excited	☐ Calm	☐ Content	☐ Bored	☐ Others ____
☐ Miserable	☐ Annoyed	☐ Afraid	☐ Droopy	☐ Angry	

Rate the intensity of the emotions you have chosen

	Not intense at all					Extremely intense
	1	2	3	4	5	6	7
» Pleased							
» Sad							
» Alarmed							
» Happy							
» Miserable							
» Relaxed							
» Serene							
» Sleepy							
» Excited							
» Annoyed							
» Frustrated							
» Satisfied							
» Tired							

» Calm						
» Afraid						
» Distressed						
» Depressed						
» Delighted						
» Content						
» Droopy						
» Astonished						
» Aroused						
» Gloomy						
» Bored						
» Angry						
» At ease						
» Glad						
» Tense						
» Others						

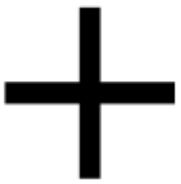

These page timer metrics will not be displayed to the recipient.

First Click: *0 seconds*

Last Click: *0 seconds*

Page Submit: *0 seconds*

Click Count: *0 clicks*

MP3: -0:34

These page timer metrics will not be displayed to the recipient.

First Click: *0 seconds*

Last Click: *0 seconds*

Page Submit: *0 seconds*

Click Count: *0 clicks*

01 59

After listening to the music, what do you feel when you look at this picture? [Pick 3]

☐ Pleased	☐ Relaxed	☐ Frustrated	☐ Distressed	☐ Astonished	☐ At ease
☐ Sad	☐ Serene	☐ Satisfied	☐ Depressed	☐ Aroused	☐ Glad
☐ Alarmed	☐ Sleepy	☐ Tired	☐ Delighted	☐ Gloomy	☐ Tense
☐ Happy	☐ Excited	☐ Calm	☐ Content	☐ Bored	☐ Others ______
☐ Miserable	☐ Annoyed	☐ Afraid	☐ Droopy	☐ Angry	

Rate the intensity of the emotions you have chosen

Not intense at all						Extremely intense
1	2	3	4	5	6	7

» Pleased						
» Sad						
» Alarmed						
» Happy						
» Miserable						
» Relaxed						
» Serene						
» Sleepy						
» Excited						
» Annoyed						
» Frustrated						
» Satisfied						
» Tired						
» Calm						
» Afraid						
» Distressed						
» Depressed						
» Delighted						
» Content						

» Droopy

» Astonished

» Aroused

» Gloomy

» Bored

» Angry

» At ease

» Glad

» Tense

» Others

+

These page timer metrics will not be displayed to the recipient.

First Click: *0 seconds*

Last Click: *0 seconds*

Page Submit: *0 seconds*

Click Count: *0 clicks*

MP3: -0:28

These page timer metrics will not be displayed to the recipient.

First Click: *0 seconds*

Last Click: *0 seconds*

Page Submit: *0 seconds*

Click Count: *0 clicks*

0159

After listening to the music, what do you feel when you look at this picture? [Pick 3]

☐ Pleased	☐ Relaxed	☐ Frustrated	☐ Distressed	☐ Astonished	☐ At ease
☐ Sad	☐ Serene	☐ Satisfied	☐ Depressed	☐ Aroused	☐ Glad
☐ Alarmed	☐ Sleepy	☐ Tired	☐ Delighted	☐ Gloomy	☐ Tense
☐ Happy	☐ Excited	☐ Calm	☐ Content	☐ Bored	☐ Others
☐ Miserable	☐ Annoyed	☐ Afraid	☐ Droopy	☐ Angry	

Rate the intensity of the emotions you have chosen

	Not intense at all					Extremely intense
	1	2	3	4	5	6	7
» Pleased							
» Sad							
» Alarmed							
» Happy							
» Miserable							
» Relaxed							
» Serene							
» Sleepy							

» Excited

» Annoyed

» Frustrated

» Satisfied

» Tired

» Calm

» Afraid

» Distressed

» Depressed

» Delighted

» Content

» Droopy

» Astonished

» Aroused

» Gloomy

» Bored

» Angry

» At ease

» Glad

» Tense						
» Others						

+

These page timer metrics will not be displayed to the recipient.

First Click: *0 seconds*

Last Click: *0 seconds*

Page Submit: *0 seconds*

Click Count: *0 clicks*

MP3: -0:20

These page timer metrics will not be displayed to the recipient.

First Click: *0 seconds*

Last Click: *0 seconds*

Page Submit: *0 seconds*

Click Count: *0 clicks*

01 59

After listening to the music, what do you feel when you look at this picture? [Pick 3]

☐ Pleased	☐ Relaxed	☐ Frustrated	☐ Distressed	☐ Astonished	☐ At ease
☐ Sad	☐ Serene	☐ Satisfied	☐ Depressed	☐ Aroused	☐ Glad
☐ Alarmed	☐ Sleepy	☐ Tired	☐ Delighted	☐ Gloomy	☐ Tense
☐ Happy	☐ Excited	☐ Calm	☐ Content	☐ Bored	☐ Others ____
☐ Miserable	☐ Annoyed	☐ Afraid	☐ Droopy	☐ Angry	

Rate the intensity of the emotions you have chosen

Not intense at all ... Extremely intense

	1	2	3	4	5	6	7
» Pleased							
» Sad							
» Alarmed							
» Happy							
» Miserable							
» Relaxed							
» Serene							
» Sleepy							
» Excited							
» Annoyed							
» Frustrated							
» Satisfied							
» Tired							
» Calm							
» Afraid							
» Distressed							

» Depressed						
» Delighted						
» Content						
» Droopy						
» Astonished						
» Aroused						
» Gloomy						
» Bored						
» Angry						
» At ease						
» Glad						
» Tense						
» Others						

+

These page timer metrics will not be displayed to the recipient.

First Click: *0 seconds*

Last Click: *0 seconds*

Page Submit: *0 seconds*

Click Count: *0 clicks*

MP3: ▶ -0:15

These page timer metrics will not be displayed to the recipient.

First Click: *0 seconds*

Last Click: *0 seconds*

Page Submit: *0 seconds*

Click Count: *0 clicks*

01 59

After listening to the music, what do you feel when you look at this picture? [Pick 3]

☐ Pleased	☐ Relaxed	☐ Frustrated	☐ Distressed	☐ Astonished	☐ At ease
☐ Sad	☐ Serene	☐ Satisfied	☐ Depressed	☐ Aroused	☐ Glad
☐ Alarmed	☐ Sleepy	☐ Tired	☐ Delighted	☐ Gloomy	☐ Tense
☐ Happy	☐ Excited	☐ Calm	☐ Content	☐ Bored	☐ Others ______
☐ Miserable	☐ Annoyed	☐ Afraid	☐ Droopy	☐ Angry	

Rate the intensity of the emotions you have chosen

	Not intense at all						Extremely intense
	1	2	3	4	5	6	7
» Pleased							
» Sad							
» Alarmed							
» Happy							
» Miserable							

» Relaxed

» Serene

» Sleepy

» Excited

» Annoyed

» Frustrated

» Satisfied

» Tired

» Calm

» Afraid

» Distressed

» Depressed

» Delighted

» Content

» Droopy

» Astonished

» Aroused

» Gloomy

» Bored

» Angry	
» At ease	
» Glad	
» Tense	
» Others	

PROCEED TO THE NEXT BLOCK

Actual experiment - Block 3

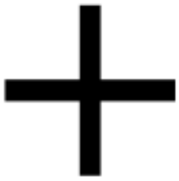

These page timer metrics will not be displayed to the recipient.
First Click: *0 seconds*
Last Click: *0 seconds*
Page Submit: *0 seconds*
Click Count: *0 clicks*

MP3: -0:20

These page timer metrics will not be displayed to the recipient.
First Click: *0 seconds*
Last Click: *0 seconds*
Page Submit: *0 seconds*
Click Count: *0 clicks*

02 00

After listening to the music, what do you feel when you look at this picture? [Pick 3]

☐ Pleased	☐ Relaxed	☐ Frustrated	☐ Distressed	☐ Astonished	☐ At ease
☐ Sad	☐ Serene	☐ Satisfied	☐ Depressed	☐ Aroused	☐ Glad
☐ Alarmed	☐ Sleepy	☐ Tired	☐ Delighted	☐ Gloomy	☐ Tense
☐ Happy	☐ Excited	☐ Calm	☐ Content	☐ Bored	☐ Others ____
☐ Miserable	☐ Annoyed	☐ Afraid	☐ Droopy	☐ Angry	

Rate the intensity of the emotions you have chosen

	Not intense at all						Extremely intense
	1	2	3	4	5	6	7
» Pleased							
» Sad							
» Alarmed							
» Happy							
» Miserable							
» Relaxed							
» Serene							
» Sleepy							
» Excited							
» Annoyed							
» Frustrated							

» Satisfied						
» Tired						
» Calm						
» Afraid						
» Distressed						
» Depressed						
» Delighted						
» Content						
» Droopy						
» Astonished						
» Aroused						
» Gloomy						
» Bored						
» Angry						
» At ease						
» Glad						
» Tense						
» Others						

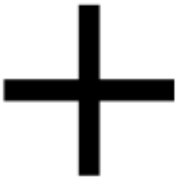

These page timer metrics will not be displayed to the recipient.
First Click: *0 seconds*
Last Click: *0 seconds*
Page Submit: *0 seconds*
Click Count: *0 clicks*

MP3: --:--

These page timer metrics will not be displayed to the recipient.
First Click: *0 seconds*
Last Click: *0 seconds*
Page Submit: *0 seconds*
Click Count: *0 clicks*

02 00

After listening to the music, what do you feel when you look at this picture? [Pick 3]

- ☐ Pleased
- ☐ Relaxed
- ☐ Frustrated
- ☐ Distressed
- ☐ Astonished
- ☐ At ease
- ☐ Sad
- ☐ Serene
- ☐ Satisfied
- ☐ Depressed
- ☐ Aroused
- ☐ Glad
- ☐ Alarmed
- ☐ Sleepy
- ☐ Tired
- ☐ Delighted
- ☐ Gloomy
- ☐ Tense
- ☐ Happy
- ☐ Excited
- ☐ Calm
- ☐ Content
- ☐ Bored
- ☐ Others ______
- ☐ Miserable
- ☐ Annoyed
- ☐ Afraid
- ☐ Droopy
- ☐ Angry

Rate the intensity of the emotions you have chosen

Not intense at all — Extremely intense

1 2 3 4 5 6 7

» Pleased						
» Sad						
» Alarmed						
» Happy						
» Miserable						
» Relaxed						
» Serene						
» Sleepy						
» Excited						
» Annoyed						
» Frustrated						
» Satisfied						
» Tired						
» Calm						
» Afraid						
» Distressed						
» Depressed						
» Delighted						
» Content						

» Droopy

» Astonished

» Aroused

» Gloomy

» Bored

» Angry

» At ease

» Glad

» Tense

» Others

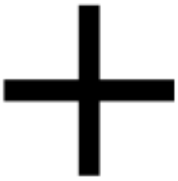

These page timer metrics will not be displayed to the recipient.

First Click: *0 seconds*

Last Click: *0 seconds*

Page Submit: *0 seconds*

Click Count: *0 clicks*

MP3: --:--

These page timer metrics will not be displayed to the recipient.

First Click: *0 seconds*

Last Click: *0 seconds*

Page Submit: *0 seconds*

Click Count: *0 clicks*

02 00

After listening to the music, what do you feel when you look at this picture? [Pick 3]

☐ Pleased	☐ Relaxed	☐ Frustrated	☐ Distressed	☐ Astonished	☐ At ease
☐ Sad	☐ Serene	☐ Satisfied	☐ Depressed	☐ Aroused	☐ Glad
☐ Alarmed	☐ Sleepy	☐ Tired	☐ Delighted	☐ Gloomy	☐ Tense
☐ Happy	☐ Excited	☐ Calm	☐ Content	☐ Bored	☐ Others
☐ Miserable	☐ Annoyed	☐ Afraid	☐ Droopy	☐ Angry	

Rate the intensity of the emotions you have chosen

	Not intense at all					Extremely intense
	1	2	3	4	5	6	7
» Pleased							
» Sad							
» Alarmed							
» Happy							
» Miserable							
» Relaxed							
» Serene							
» Sleepy							

» Excited						
» Annoyed						
» Frustrated						
» Satisfied						
» Tired						
» Calm						
» Afraid						
» Distressed						
» Depressed						
» Delighted						
» Content						
» Droopy						
» Astonished						
» Aroused						
» Gloomy						
» Bored						
» Angry						
» At ease						
» Glad						

» Tense	
» Others	

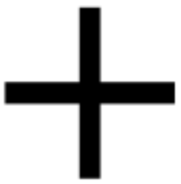

These page timer metrics will not be displayed to the recipient.

First Click: *0 seconds*

Last Click: *0 seconds*

Page Submit: *0 seconds*

Click Count: *0 clicks*

MP3: -0:15

These page timer metrics will not be displayed to the recipient.

First Click: *0 seconds*

Last Click: *0 seconds*

Page Submit: *0 seconds*

Click Count: *0 clicks*

02 00

After listening to the music, what do you feel when you look at this picture? [Pick 3]

☐ Pleased	☐ Relaxed	☐ Frustrated	☐ Distressed	☐ Astonished	☐ At ease
☐ Sad	☐ Serene	☐ Satisfied	☐ Depressed	☐ Aroused	☐ Glad
☐ Alarmed	☐ Sleepy	☐ Tired	☐ Delighted	☐ Gloomy	☐ Tense
☐ Happy	☐ Excited	☐ Calm	☐ Content	☐ Bored	☐ Others ______
☐ Miserable	☐ Annoyed	☐ Afraid	☐ Droopy	☐ Angry	

Rate the intensity of the emotions you have chosen

	Not intense at all					Extremely intense
	1	2	3	4	5	6	7
» Pleased							
» Sad							
» Alarmed							
» Happy							
» Miserable							
» Relaxed							
» Serene							
» Sleepy							
» Excited							
» Annoyed							
» Frustrated							
» Satisfied							
» Tired							
» Calm							
» Afraid							
» Distressed							

» Depressed	
» Delighted	
» Content	
» Droopy	
» Astonished	
» Aroused	
» Gloomy	
» Bored	
» Angry	
» At ease	
» Glad	
» Tense	
» Others	

+

These page timer metrics will not be displayed to the recipient.

First Click: *0 seconds*

Last Click: *0 seconds*

Page Submit: *0 seconds*

Click Count: *0 clicks*

MP3: ▶ --:--

These page timer metrics will not be displayed to the recipient.

First Click: *0 seconds*

Last Click: *0 seconds*

Page Submit: *0 seconds*

Click Count: *0 clicks*

02:00

After listening to the music, what do you feel when you look at this picture? [Pick 3]

☐ Pleased	☐ Relaxed	☐ Frustrated	☐ Distressed	☐ Astonished	☐ At ease
☐ Sad	☐ Serene	☐ Satisfied	☐ Depressed	☐ Aroused	☐ Glad
☐ Alarmed	☐ Sleepy	☐ Tired	☐ Delighted	☐ Gloomy	☐ Tense
☐ Happy	☐ Excited	☐ Calm	☐ Content	☐ Bored	☐ Others ______
☐ Miserable	☐ Annoyed	☐ Afraid	☐ Droopy	☐ Angry	

Rate the intensity of the emotions you have chosen

Not intense at all — Extremely intense

	1	2	3	4	5	6	7
» Pleased							
» Sad							
» Alarmed							
» Happy							
» Miserable							

» Relaxed						
» Serene						
» Sleepy						
» Excited						
» Annoyed						
» Frustrated						
» Satisfied						
» Tired						
» Calm						
» Afraid						
» Distressed						
» Depressed						
» Delighted						
» Content						
» Droopy						
» Astonished						
» Aroused						
» Gloomy						
» Bored						

» Angry						
» At ease						
» Glad						
» Tense						
» Others						

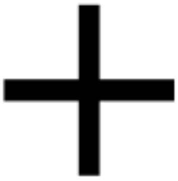

These page timer metrics will not be displayed to the recipient.
First Click: *0 seconds*
Last Click: *0 seconds*
Page Submit: *0 seconds*
Click Count: *0 clicks*

MP3: -0:15

These page timer metrics will not be displayed to the recipient.
First Click: *0 seconds*
Last Click: *0 seconds*
Page Submit: *0 seconds*
Click Count: *0 clicks*

02 00

After listening to the music, what do you feel when you look at this picture? [Pick 3]

☐ Pleased	☐ Relaxed	☐ Frustrated	☐ Distressed	☐ Astonished	☐ At ease
☐ Sad	☐ Serene	☐ Satisfied	☐ Depressed	☐ Aroused	☐ Glad
☐ Alarmed	☐ Sleepy	☐ Tired	☐ Delighted	☐ Gloomy	☐ Tense
☐ Happy	☐ Excited	☐ Calm	☐ Content	☐ Bored	☐ Others ______
☐ Miserable	☐ Annoyed	☐ Afraid	☐ Droopy	☐ Angry	

Rate the intensity of the emotions you have chosen

	Not intense at all					Extremely intense	
	1	2	3	4	5	6	7
» Pleased							
» Sad							
» Alarmed							
» Happy							
» Miserable							
» Relaxed							
» Serene							
» Sleepy							
» Excited							
» Annoyed							
» Frustrated							
» Satisfied							
» Tired							

» Calm

» Afraid

» Distressed

» Depressed

» Delighted

» Content

» Droopy

» Astonished

» Aroused

» Gloomy

» Bored

» Angry

» At ease

» Glad

» Tense

» Others

PROCEED TO THE NEXT BLOCK

Actual experiment - Block 4

These page timer metrics will not be displayed to the recipient.

First Click: *0 seconds*

Last Click: *0 seconds*

Page Submit: *0 seconds*

Click Count: *0 clicks*

MP3: --:--

These page timer metrics will not be displayed to the recipient.

First Click: *0 seconds*

Last Click: *0 seconds*

Page Submit: *0 seconds*

Click Count: *0 clicks*

02 00

After listening to the music, what do you feel when you look at this picture? [Pick 3]

☐ Pleased	☐ Relaxed	☐ Frustrated	☐ Distressed	☐ Astonished	☐ At ease
☐ Sad	☐ Serene	☐ Satisfied	☐ Depressed	☐ Aroused	☐ Glad
☐ Alarmed	☐ Sleepy	☐ Tired	☐ Delighted	☐ Gloomy	☐ Tense
☐ Happy	☐ Excited	☐ Calm	☐ Content	☐ Bored	☐ Others ______
☐ Miserable	☐ Annoyed	☐ Afraid	☐ Droopy	☐ Angry	

Rate the intensity of the emotions you have chosen

	Not intense at all					Extremely intense
1	2	3	4	5	6	7

» Pleased						
» Sad						
» Alarmed						
» Happy						
» Miserable						
» Relaxed						
» Serene						
» Sleepy						
» Excited						
» Annoyed						
» Frustrated						
» Satisfied						
» Tired						
» Calm						
» Afraid						
» Distressed						
» Depressed						
» Delighted						
» Content						

» Droopy

» Astonished

» Aroused

» Gloomy

» Bored

» Angry

» At ease

» Glad

» Tense

» Others

+

These page timer metrics will not be displayed to the recipient.

First Click: *0 seconds*

Last Click: *0 seconds*

Page Submit: *0 seconds*

Click Count: *0 clicks*

MP3: --:--

These page timer metrics will not be displayed to the recipient.

First Click: *0 seconds*

Last Click: *0 seconds*

Page Submit: *0 seconds*

Click Count: *0 clicks*

0200

After listening to the music, what do you feel when you look at this picture? [Pick 3]

☐ Pleased	☐ Relaxed	☐ Frustrated	☐ Distressed	☐ Astonished	☐ At ease
☐ Sad	☐ Serene	☐ Satisfied	☐ Depressed	☐ Aroused	☐ Glad
☐ Alarmed	☐ Sleepy	☐ Tired	☐ Delighted	☐ Gloomy	☐ Tense
☐ Happy	☐ Excited	☐ Calm	☐ Content	☐ Bored	☐ Others ______
☐ Miserable	☐ Annoyed	☐ Afraid	☐ Droopy	☐ Angry	

Rate the intensity of the emotions you have chosen

Not intense at all — Extremely intense

	1	2	3	4	5	6	7
» Pleased							
» Sad							
» Alarmed							
» Happy							
» Miserable							
» Relaxed							
» Serene							
» Sleepy							

» Excited

» Annoyed

» Frustrated

» Satisfied

» Tired

» Calm

» Afraid

» Distressed

» Depressed

» Delighted

» Content

» Droopy

» Astonished

» Aroused

» Gloomy

» Bored

» Angry

» At ease

» Glad

» Tense

» Others

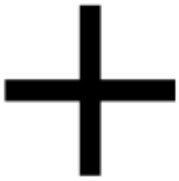

These page timer metrics will not be displayed to the recipient.**

First Click: *0 seconds*

Last Click: *0 seconds*

Page Submit: *0 seconds*

Click Count: *0 clicks*

MP3: -00:00

These page timer metrics will not be displayed to the recipient.

First Click: *0 seconds*

Last Click: *0 seconds*

Page Submit: *0 seconds*

Click Count: *0 clicks*

02 00

What do you feel when you look at this picture? [Pick 3]

☐ Pleased	☐ Relaxed	☐ Frustrated	☐ Distressed	☐ Astonished	☐ At ease
☐ Sad	☐ Serene	☐ Satisfied	☐ Depressed	☐ Aroused	☐ Glad
☐ Alarmed	☐ Sleepy	☐ Tired	☐ Delighted	☐ Gloomy	☐ Tense
☐ Happy	☐ Excited	☐ Calm	☐ Content	☐ Bored	☐ Others
☐ Miserable	☐ Annoyed	☐ Afraid	☐ Droopy	☐ Angry	

Rate the intensity of the emotions you have chosen

	1	2	3	4	5	6	7
	Not intense at all						Extremely intense
» Pleased							
» Sad							
» Alarmed							
» Happy							
» Miserable							
» Relaxed							
» Serene							
» Sleepy							
» Excited							
» Annoyed							
» Frustrated							
» Satisfied							
» Tired							
» Calm							
» Afraid							
» Distressed							

» Depressed						
» Delighted						
» Content						
» Droopy						
» Astonished						
» Aroused						
» Gloomy						
» Bored						
» Angry						
» At ease						
» Glad						
» Tense						
» Others						

+

These page timer metrics will not be displayed to the recipient.

First Click: *0 seconds*

Last Click: *0 seconds*

Page Submit: *0 seconds*

Click Count: *0 clicks*

MP3: ▶ -00:00

These page timer metrics will not be displayed to the recipient.

First Click: *0 seconds*

Last Click: *0 seconds*

Page Submit: *0 seconds*

Click Count: *0 clicks*

02 00

What do you feel when you look at this picture? [Pick 3]

☐ Pleased	☐ Relaxed	☐ Frustrated	☐ Distressed	☐ Astonished	☐ At ease
☐ Sad	☐ Serene	☐ Satisfied	☐ Depressed	☐ Aroused	☐ Glad
☐ Alarmed	☐ Sleepy	☐ Tired	☐ Delighted	☐ Gloomy	☐ Tense
☐ Happy	☐ Excited	☐ Calm	☐ Content	☐ Bored	☐ Others
☐ Miserable	☐ Annoyed	☐ Afraid	☐ Droopy	☐ Angry	

Rate the intensity of the emotions you have chosen

Not intense at all — Extremely intense

	1	2	3	4	5	6	7
» Pleased							
» Sad							
» Alarmed							
» Happy							
» Miserable							

» Relaxed						
» Serene						
» Sleepy						
» Excited						
» Annoyed						
» Frustrated						
» Satisfied						
» Tired						
» Calm						
» Afraid						
» Distressed						
» Depressed						
» Delighted						
» Content						
» Droopy						
» Astonished						
» Aroused						
» Gloomy						
» Bored						

» Angry						
» At ease						
» Glad						
» Tense						
» Others						

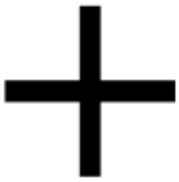

These page timer metrics will not be displayed to the recipient.
First Click: *0 seconds*
Last Click: *0 seconds*
Page Submit: *0 seconds*
Click Count: *0 clicks*

MP3: -00:00

These page timer metrics will not be displayed to the recipient.
First Click: *0 seconds*
Last Click: *0 seconds*
Page Submit: *0 seconds*
Click Count: *0 clicks*

02 00

After listening to the music, what do you feel when you look at this picture? [Pick 3]

☐ Pleased	☐ Relaxed	☐ Frustrated	☐ Distressed	☐ Astonished	☐ At ease
☐ Sad	☐ Serene	☐ Satisfied	☐ Depressed	☐ Aroused	☐ Glad
☐ Alarmed	☐ Sleepy	☐ Tired	☐ Delighted	☐ Gloomy	☐ Tense
☐ Happy	☐ Excited	☐ Calm	☐ Content	☐ Bored	☐ Others ______
☐ Miserable	☐ Annoyed	☐ Afraid	☐ Droopy	☐ Angry	

Rate the intensity of the emotions you have chosen

Not intense at all — Extremely intense

	1	2	3	4	5	6	7
» Pleased							
» Sad							
» Alarmed							
» Happy							
» Miserable							
» Relaxed							
» Serene							
» Sleepy							
» Excited							
» Annoyed							
» Frustrated							
» Satisfied							
» Tired							

» Calm						
» Afraid						
» Distressed						
» Depressed						
» Delighted						
» Content						
» Droopy						
» Astonished						
» Aroused						
» Gloomy						
» Bored						
» Angry						
» At ease						
» Glad						
» Tense						
» Others						

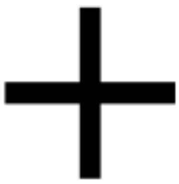

These page timer metrics will not be displayed to the recipient.
First Click: *0 seconds*
Last Click: *0 seconds*
Page Submit: *0 seconds*
Click Count: *0 clicks*

MP3: -00:00

These page timer metrics will not be displayed to the recipient.
First Click: *0 seconds*
Last Click: *0 seconds*
Page Submit: *0 seconds*
Click Count: *0 clicks*

02 00

After listening to the music, what do you feel when you look at this picture? [Pick 3]

☐ Pleased	☐ Relaxed	☐ Frustrated	☐ Distressed	☐ Astonished	☐ At ease
☐ Sad	☐ Serene	☐ Satisfied	☐ Depressed	☐ Aroused	☐ Glad
☐ Alarmed	☐ Sleepy	☐ Tired	☐ Delighted	☐ Gloomy	☐ Tense
☐ Happy	☐ Excited	☐ Calm	☐ Content	☐ Bored	☐ Others ______
☐ Miserable	☐ Annoyed	☐ Afraid	☐ Droopy	☐ Angry	

Rate the intensity of the emotions you have chosen

Not intense at all						Extremely intense
1	2	3	4	5	6	7

» Pleased						
» Sad						
» Alarmed						
» Happy						
» Miserable						
» Relaxed						
» Serene						
» Sleepy						
» Excited						
» Annoyed						
» Frustrated						
» Satisfied						
» Tired						
» Calm						
» Afraid						
» Distressed						
» Depressed						
» Delighted						
» Content						

» Droopy						
» Astonished						
» Aroused						
» Gloomy						
» Bored						
» Angry						
» At ease						
» Glad						
» Tense						
» Others						

PROCEED TO THE NEXT BLOCK

Actual experiment - Block 5

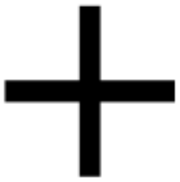

These page timer metrics will not be displayed to the recipient.

First Click: *0 seconds*

Last Click: *0 seconds*
Page Submit: *0 seconds*
Click Count: *0 clicks*

MP3: -00:00

These page timer metrics will not be displayed to the recipient.
First Click: *0 seconds*
Last Click: *0 seconds*
Page Submit: *0 seconds*
Click Count: *0 clicks*

02:00

After listening to the music, what do you feel when you look at this picture? [Pick 3]

☐ Pleased	☐ Relaxed	☐ Frustrated	☐ Distressed	☐ Astonished	☐ At ease
☐ Sad	☐ Serene	☐ Satisfied	☐ Depressed	☐ Aroused	☐ Glad
☐ Alarmed	☐ Sleepy	☐ Tired	☐ Delighted	☐ Gloomy	☐ Tense
☐ Happy	☐ Excited	☐ Calm	☐ Content	☐ Bored	☐ Others ____
☐ Miserable	☐ Annoyed	☐ Afraid	☐ Droopy	☐ Angry	

Rate the intensity of the emotions you have chosen

	Not intense at all						Extremely intense
	1	2	3	4	5	6	7
» Pleased							
» Sad							
» Alarmed							
» Happy							
» Miserable							

» Relaxed						
» Serene						
» Sleepy						
» Excited						
» Annoyed						
» Frustrated						
» Satisfied						
» Tired						
» Calm						
» Afraid						
» Distressed						
» Depressed						
» Delighted						
» Content						
» Droopy						
» Astonished						
» Aroused						
» Gloomy						
» Bored						

» Angry						
» At ease						
» Glad						
» Tense						
» Others						
