# Supplementary material for: Effects of affective priming through music on the use of emotion words
Source: PLoS One. 2019 Apr 16;14(4):e0214482. doi: 10.1371/journal.pone.0214482 (PMC6467386; doi:10.1371/journal.pone.0214482)
Supplement: S3 File — (PDF) [file pone.0214482.s003.pdf]

Mode	Composer	Composition
Major	Haydn	Sonata in G major, Hob XVI.8 , 4th movement, Allegro.<br>Original score link:<br>https://imslp.org/wiki/Special:ReverseLookup/471961
Major	Haydn	Sonata in G major, Hob XVI, 11, 1st movement, Presto.<br>Original score link:<br>https://imslp.org/wiki/Special:ReverseLookup/471965
Major	Haydn	Sonata in G major, Hob XVI, 27, 1st movement, Allegro.<br>Original score link:<br>https://imslp.org/wiki/Special:ReverseLookup/120748
Minor	Schumann	Sonata in G minor, Allegro.<br>Original score link:<br>https://imslp.org/wiki/Special:ReverseLookup/377000
Minor	Beethoven	Piano Sonata No.19 In G Minor, Op.49 No.1: 1. Andante.<br>Original score link:<br>https://imslp.org/wiki/Special:ReverseLookup/51743
