# Supplementary material for: Effects of affective priming through music on the use of emotion words
Source: PLoS One. 2019 Apr 16;14(4):e0214482. doi: 10.1371/journal.pone.0214482 (PMC6467386; doi:10.1371/journal.pone.0214482)
Supplement: S5 File — (PDF) [file pone.0214482.s005.pdf]

**S7_Appendix. Links to Images Used in Testing**

Due to licensing restrictions, the authors have listed links to images purchased under a Standard License from Adobe Stock Library for the use of testing during this study. One image is no longer available on the Adobe Stock Library and an alternative source has been provided.

| Name of Image | Adobe File ID | Author | Web Link |
|---|---|---|---|
| continuous line drawing of boy making photos with camera | 128826289 | One Line Man | https://stock.adobe.com/images/continuous-line-drawing-of-boy-making-photos-with-camera/128826289 |
| continuous line drawing of businessman thinking on white background | 120599521 | One Line Man | https://stock.adobe.com/images/continuous-line-drawing-of-businessman-thinking-on-white-background/120599521 |
| businessman writing a note - single line drawing | 157116687 | One Line Man | https://stock.adobe.com/images/businessman-writing-a-note-single-line-drawing/157116687 |
| continuous line drawing of business concept - man before open door of opportunity | 173139144 | One Line Man | https://stock.adobe.com/images/continuous-line-drawing-of-business-concept-man-before-open-door-of-opportunity/173139144 |
| one line drawing of man grilling barbecue | 178589010 | One Line Man | https://stock.adobe.com/images/one-line-drawing-of-man-grilling-barbecue/178589010 |
| continuous line drawing of young man talking on mobile phone | 132179712 | One Line Man | https://stock.adobe.com/images/continuous-line-drawing-of-young-man-talking-on-mobile-phone/132179712 |
| businessman making phone call - continuous line drawing | 152529686 | One Line Man | https://stock.adobe.com/images/businessman-making-phone-call-continuous-line-drawing/152529686 |
| one line drawing of man working with laptop computer behind desk | 178588921 | One Line Man | https://stock.adobe.com/images/one-line-drawing-of-man-working-with-laptop-computer-behind-desk/178588921 |
| Continuous line drawing. Woman relaxing with cup of tea. Vector illustration. | 171728496 | Valenty | https://stock.adobe.com/images/continuous-line-drawing-woman-relaxing-with-cup-of-tea-vector-illustration/171728496 |
| Continuous line drawing. Young Boy Playing His Toy Car. Vector illustration | 163681640 | Valenty | https://stock.adobe.com/images/continuous-line-drawing-young-boy-playing-his-toy-car-vector-illustration/163681640 |

| | | | |
|---|---|---|---|
| Man in fez reading Koran. Continuous different width line drawing vector illustration | 179745135 | Valenty | https://stock.adobe.com/images/man-in-fez-reading-koran-continuous-different-width-line-drawing-vector-illustration/179745135 |
| one line drawing of two sitting men talking | 178592581 | One Line Man | https://stock.adobe.com/images/one-line-drawing-of-two-sitting-men-talking/178592581 |
| Continuous line drawing. Man in sleeping pose on pillow. Vector illustration | 178712883 | Valenty | https://stock.adobe.com/images/continuous-line-drawing-man-in-sleeping-pose-on-pillow-vector-illustration/178712883 |
| continuous line drawing of a guy sitting with laptop computer | 130164167 | One Line Man | https://stock.adobe.com/images/continuous-line-drawing-of-a-guy-sitting-with-laptop-computer/130164167 |
| Kids with books. Back to school concept. | [no longer available on Adobe] | Valenty | https://creativemarket.com/Valenty/1696709-Kids-with-books.-Back-to-school-concept. |
| one line drawing of commuters passing by | 170087272 | One Line Man | https://stock.adobe.com/images/one-line-drawing-of-commuters-passing-by/170087272 |
| one line drawing of man sitting and reading | 170087294 | One Line Man | https://stock.adobe.com/images/one-line-drawing-of-man-sitting-and-reading/170087294 |
| one line drawing of travelers walking | 178592568 | One Line Man | https://stock.adobe.com/images/one-line-drawing-of-travelers-walking/178592568 |
| Continuous line drawing. Father and son sitting together. Vector illustration Total editable, choose thickness and place of line | 176884710 | Valenty | https://stock.adobe.com/images/continuous-line-drawing-father-and-son-sitting-together-vector-illustration-total-editable-choose-thickness-and-place-of-line/176884710 |
| continuous line drawing of youth soccer players | 120537731 | One Line Man | https://stock.adobe.com/images/continuous-line-drawing-of-youth-soccer-players/120537731 |
| Street musician man playing the violin. Continuous line black and white drawing. Lineart vector illustration | 168651794 | Valenty | https://stock.adobe.com/images/street-musician-man-playing-the-violin-continuous-line-black-and-white-drawing-lineart-vector-illustration/168651794 |
| Boy and girl drawing on paper. Back to school concept. Continuous line | 164739887 | Valenty | https://stock.adobe.com/images/boy-and-girl-drawing-on-paper-back-to-school-concept-continuous-line-drawing-vector-illustration-on-white-background/164739887 |

| | | | |
|---|---|---|---|
| drawing. Vector illustration on white background | | | |
| _continuous-line-drawing-template | 137418165 | One Line Man | https://stock.adobe.com/images/continuous-line-drawing-template/137418165 |
| Continuous line drawing. Abstract portrait of a woman with cup of tea. Vector illustration. | 159501133 | Valenty | https://stock.adobe.com/images/continuous-line-drawing-abstract-portrait-of-a-woman-with-cup-of-tea-vector-illustration/159501133 |
| Teacher with boy writting on Chalk school board . Continuous line drawing vector illustration back to school | 162611086 | Valenty | https://stock.adobe.com/images/teacher-with-boy-writting-on-chalk-school-board-continuous-line-drawing-vector-illustration-back-to-school/162611086 |
| Man in fez reading Koran. Continuous line drawing vector illustration | 158333266 | Valenty | https://stock.adobe.com/images/man-in-fez-reading-koran-continuous-line-drawing-vector-illustration/158333266 |
| one line drawing of group of young people talking | 178588907 | One Line Man | https://stock.adobe.com/images/one-line-drawing-of-group-of-young-people-talking/178588907 |
| one line drawing of man walking with a phone | 178588874 | One Line Man | https://stock.adobe.com/images/one-line-drawing-of-man-walking-with-a-phone/178588874 |
| continuous line drawing of team meeting | 170078880 | One Line Man | https://stock.adobe.com/images/continuous-line-drawing-of-team-meeting/170078880 |
| continuous line drawing of people working on laptop computers | 170076371 | One Line Man | https://stock.adobe.com/images/continuous-line-drawing-of-people-working-on-laptop-computers/170076371 |
| businessman working on laptop computer - single line drawing | 157116752 | One Line Man | https://stock.adobe.com/images/businessman-working-on-laptop-computer-single-line-drawing/157116752 |
| Continuous line drawing. Little boy sitting with teddy bear on the white background. Vector illustration | 178714978 | Valenty | https://stock.adobe.com/images/continuous-line-drawing-little-boy-sitting-with-teddy-bear-on-the-white-background-vector-illustration/178714978 |
| continuous line drawing of travelling businessman walking with r | 130315399 | One Line Man | https://stock.adobe.com/images/continuous-line-drawing-of-travelling-businessman-walking-with-r/130315399 |
