# Supplementary material for: Effects of affective priming through music on the use of emotion words
Source: PLoS One. 2019 Apr 16;14(4):e0214482. doi: 10.1371/journal.pone.0214482 (PMC6467386; doi:10.1371/journal.pone.0214482)
Supplement: S6 File — (PDF) [file pone.0214482.s006.pdf]

qualtrics. FREE ACCOUNT

Rating

Without relating to your personal/past experiences, please take a look at the following pictures and rate the pictures on a scale of 1-7, 1 - Emotionally positive, 7 - Emotionally negative.

Picture

Emotionally positive			Emotionally ambiguous			Emotionally negative
1	2	3	4	5	6	7

Picture

Emotionally positive			Emotionally ambiguous			Emotionally negative
1	2	3	4	5	6	7

Picture

Emotionally positive			Emotionally ambiguous			Emotionally negative
1	2	3	4	5	6	7

Picture

Emotionally

Emotionally positive ambiguous Emotionally negative

1 2 3 4 5 6 7

Picture

Emotionally positive | Emotionally ambiguous | Emotionally negative

1 2 3 4 5 6 7

Picture

Emotionally positive | Emotionally ambiguous | Emotionally negative

1 2 3 4 5 6 7

Picture

Emotionally positive | Emotionally ambiguous | Emotionally negative

1 2 3 4 5 6 7

Picture

Emotionally positive | Emotionally ambiguous | Emotionally negative

1 2 3 4 5 6 7

Picture

Emotionally positive | Emotionally ambiguous | Emotionally negative

1 2 3 4 5 6 7

Picture

Emotionally positive | Emotionally ambiguous | Emotionally negative

1 2 3 4 5 6 7

Picture

Emotionally positive | Emotionally ambiguous | Emotionally negative

1 2 3 4 5 6 7

Picture

Emotionally positive | Emotionally ambiguous | Emotionally negative

1 2 3 4 5 6 7

Picture

Emotionally positive | Emotionally ambiguous | Emotionally negative

1 2 3 4 5 6 7

Picture

Emotionally positive | Emotionally ambiguous | Emotionally negative

1 2 3 4 5 6 7

Picture

Emotionally positive | Emotionally ambiguous | Emotionally negative

1 2 3 4 5 6 7

Picture

Emotionally positive | Emotionally ambiguous | Emotionally negative

1 2 3 4 5 6 7

Picture

Emotionally positive | Emotionally ambiguous | Emotionally negative

1 2 3 4 5 6 7

Picture

Emotionally positive | Emotionally ambiguous | Emotionally negative

1 2 3 4 5 6 7

Picture

Emotionally positive | Emotionally ambiguous | Emotionally negative

1 2 3 4 5 6 7

Picture

Emotionally positive			Emotionally ambiguous			Emotionally negative
1	2	3	4	5	6	7

Picture

Emotionally positive			Emotionally ambiguous			Emotionally negative
1	2	3	4	5	6	7

Picture

Emotionally positive			Emotionally ambiguous			Emotionally negative
1	2	3	4	5	6	7

Picture

Emotionally positive			Emotionally ambiguous			Emotionally negative
1	2	3	4	5	6	7

Picture

Emotionally positive			Emotionally ambiguous			Emotionally negative
1	2	3	4	5	6	7

Picture

Emotionally positive			Emotionally ambiguous			Emotionally negative
1	2	3	4	5	6	7

Picture

Emotionally positive			Emotionally ambiguous			Emotionally negative
1	2	3	4	5	6	7

Picture

Emotionally positive			Emotionally ambiguous			Emotionally negative
1	2	3	4	5	6	7

Picture

Emotionally positive			Emotionally ambiguous			Emotionally negative
1	2	3	4	5	6	7

Picture

Emotionally positive			Emotionally ambiguous			Emotionally negative
1	2	3	4	5	6	7

Picture

Emotionally positive			Emotionally ambiguous			Emotionally negative
1	2	3	4	5	6	7

Demographic survey

Your age as of 1st Jan 2018

What is your gender?

- Male
- Female

Report Abuse

Powered by Qualtrics
