# Supplementary material for: Effects of affective priming through music on the use of emotion words
Source: PLoS One. 2019 Apr 16;14(4):e0214482. doi: 10.1371/journal.pone.0214482 (PMC6467386; doi:10.1371/journal.pone.0214482)
Supplement: S1 Dataset — (ZIP) [file pone.0214482.s007.zip › S1 Dataset. Data of the experiment. /Data analysis output.pdf]

```
Your temporary usage period for IBM SPSS Statistics will expire in 14 days.


GET DATA
  /TYPE=XLSX
  /FILE='/Users/tayyulingrosabel/Desktop/FYP /Data to submit /Data files an
d analysis output/Collated data .xlsm'
  /SHEET=name 'Collated responses'
  /CELLRANGE=FULL
  /READNAMES=ON
  /DATATYPEMIN PERCENTAGE=95.0
  /HIDDEN IGNORE=YES.
EXECUTE.
DATASET NAME DataSet1 WINDOW=FRONT.
NONPAR CORR
  /VARIABLES=MajF_positive MajO_positive MajS_positive MinF_positive MinO_p
ositive MinS_positive
    Mood
  /PRINT=SPEARMAN ONETAIL NOSIG
  /MISSING=PAIRWISE.
```

Nonparametric Correlations

Notes

Output Created		24-MAR-2018 17:55...
Comments		
Input	Active Dataset	DataSet1
	Filter	<none>
	Weight	<none>
	Split File	<none>
	N of Rows in Working Data File	52
Missing Value Handling	Definition of Missing	User-defined missing values are treated as missing.
	Cases Used	Statistics for each pair of variables are based on all the cases with valid data for that pair.

Notes

Syntax		NONPAR CORR<br><br>/VARIABLES=MajF_positive MajO_positive MajS_positive MinF_positive MinO_positive MinS_positive Mood<br>/PRINT=SPEARMAN ONETAIL NOSIG<br>/MISSING=PAIRWISE.
Resources	Processor Time	00:00:00.02
	Elapsed Time	00:00:00.00
	Number of Cases Allowed	314572 cases [a]

a. Based on availability of workspace memory

```
[DataSet1]
```

Correlations

			MajF_positive	MajO_positive
Spearman's rho	MajF_positive	Correlation Coefficient	1.000	.260*
		Sig. (1-tailed)	.	.036
		N	49	49
	MajO_positive	Correlation Coefficient	.260*	1.000
		Sig. (1-tailed)	.036	.
		N	49	49
	MajS_positive	Correlation Coefficient	-.077	.207
		Sig. (1-tailed)	.299	.077
		N	49	49
	MinF_positive	Correlation Coefficient	-.026	-.318*
		Sig. (1-tailed)	.431	.013
		N	49	49
	MinO_positive	Correlation Coefficient	-.054	-.177
		Sig. (1-tailed)	.356	.112
		N	49	49
	MinS_positive	Correlation Coefficient	-.117	-.271*
		Sig. (1-tailed)	.211	.030
		N	49	49
	Mood	Correlation Coefficient	-.038	.176
		Sig. (1-tailed)	.398	.113
		N	49	49

Correlations

			MajS_positive	MinF_positive
Spearman's rho	MajF_positive	Correlation Coefficient	-.077	-.026
		Sig. (1-tailed)	.299	.431
		N	49	49
	MajO_positive	Correlation Coefficient	.207	-.318*
		Sig. (1-tailed)	.077	.013
		N	49	49
	MajS_positive	Correlation Coefficient	1.000	-.057
		Sig. (1-tailed)	.	.350
		N	49	49
	MinF_positive	Correlation Coefficient	-.057	1.000
		Sig. (1-tailed)	.350	.
		N	49	49
	MinO_positive	Correlation Coefficient	.133	.399**
		Sig. (1-tailed)	.182	.002
		N	49	49
	MinS_positive	Correlation Coefficient	.144	.501**
		Sig. (1-tailed)	.162	.000
		N	49	49
	Mood	Correlation Coefficient	-.122	-.149
		Sig. (1-tailed)	.201	.154
		N	49	49

Correlations

			MinO_positive	MinS_positive
Spearman's rho	MajF_positive	Correlation Coefficient	-.054	-.117
		Sig. (1-tailed)	.356	.211
		N	49	49
	MajO_positive	Correlation Coefficient	-.177	-.271*
		Sig. (1-tailed)	.112	.030
		N	49	49
	MajS_positive	Correlation Coefficient	.133	.144
		Sig. (1-tailed)	.182	.162
		N	49	49
	MinF_positive	Correlation Coefficient	.399**	.501**
		Sig. (1-tailed)	.002	.000
		N	49	49
	MinO_positive	Correlation Coefficient	1.000	.469**
		Sig. (1-tailed)	.	.000
		N	49	49
	MinS_positive	Correlation Coefficient	.469**	1.000
		Sig. (1-tailed)	.000	.
		N	49	49
	Mood	Correlation Coefficient	.129	-.017
		Sig. (1-tailed)	.188	.455
		N	49	49

Correlations

			Mood
Spearman's rho	MajF_positive	Correlation Coefficient	-.038
		Sig. (1-tailed)	.398
		N	49
	MajO_positive	Correlation Coefficient	.176
		Sig. (1-tailed)	.113
		N	49
	MajS_positive	Correlation Coefficient	-.122
		Sig. (1-tailed)	.201
		N	49
	MinF_positive	Correlation Coefficient	-.149
		Sig. (1-tailed)	.154
		N	49
	MinO_positive	Correlation Coefficient	.129
		Sig. (1-tailed)	.188
		N	49
	MinS_positive	Correlation Coefficient	-.017
		Sig. (1-tailed)	.455
		N	49
	Mood	Correlation Coefficient	1.000
		Sig. (1-tailed)	.
		N	49

*. Correlation is significant at the 0.05 level (1-tailed).

**. Correlation is significant at the 0.01 level (1-tailed).

```
SAVE OUTFILE='/Users/tayyulingrosabel/Desktop/FYP /Data to submit /Data fil
es and analysis '+
    'output/SPSS data.sav'
  /COMPRESSED.
NONPAR CORR
  /VARIABLES=Mood MajF_HA MajO_HA MajS_HA MinF_HA MinO_HA MinS_HA
  /PRINT=SPEARMAN ONETAIL NOSIG
  /MISSING=PAIRWISE.
```

Nonparametric Correlations

Notes

Output Created		24-MAR-2018 17:58...
Comments		
Input	Data	/Users/tayyulingrosabel/Desktop/FYP /Data to submit /Data files and analysis output/SPSS data.sav
	Active Dataset	DataSet1
	Filter	<none>
	Weight	<none>
	Split File	<none>
	N of Rows in Working Data File	52
Missing Value Handling	Definition of Missing	User-defined missing values are treated as missing.
	Cases Used	Statistics for each pair of variables are based on all the cases with valid data for that pair.
Syntax		NONPAR CORR /VARIABLES=Mood MajF_HA MajO_HA MajS_HA MinF_HA MinO_HA MinS_HA /PRINT=SPEARMAN ONETAIL NOSIG /MISSING=PAIRWISE.
Resources	Processor Time	00:00:00.01
	Elapsed Time	00:00:00.00
	Number of Cases Allowed	314572 cases [a]

a. Based on availability of workspace memory

```
[DataSet1] /Users/tayyulingrosabel/Desktop/FYP /Data to submit /Data files
and analysis output/SPSS data.sav
```

Correlations

			Mood	MajF_HA	MajO_HA
Spearman's rho	Mood	Correlation Coefficient	1.000	.004	-.187
		Sig. (1-tailed)	.	.489	.100
		N	49	49	49
	MajF_HA	Correlation Coefficient	.004	1.000	.202
		Sig. (1-tailed)	.489	.	.082
		N	49	49	49
	MajO_HA	Correlation Coefficient	-.187	.202	1.000
		Sig. (1-tailed)	.100	.082	.
		N	49	49	49
	MajS_HA	Correlation Coefficient	.016	-.014	.185
		Sig. (1-tailed)	.458	.462	.101
		N	49	49	49
	MinF_HA	Correlation Coefficient	.143	.435**	.015
		Sig. (1-tailed)	.164	.001	.460
		N	49	49	49
	MinO_HA	Correlation Coefficient	-.147	.366**	.200
		Sig. (1-tailed)	.156	.005	.084
		N	49	49	49
	MinS_HA	Correlation Coefficient	.022	.053	-.072
		Sig. (1-tailed)	.441	.358	.311
		N	49	49	49

Correlations

			MajS_HA	MinF_HA	MinO_HA
Spearman's rho	Mood	Correlation Coefficient	.016	.143	-.147
		Sig. (1-tailed)	.458	.164	.156
		N	49	49	49
	MajF_HA	Correlation Coefficient	-.014	.435**	.366**
		Sig. (1-tailed)	.462	.001	.005
		N	49	49	49
	MajO_HA	Correlation Coefficient	.185	.015	.200
		Sig. (1-tailed)	.101	.460	.084
		N	49	49	49
	MajS_HA	Correlation Coefficient	1.000	-.184	.176
		Sig. (1-tailed)	.	.103	.113
		N	49	49	49
	MinF_HA	Correlation Coefficient	-.184	1.000	.382**
		Sig. (1-tailed)	.103	.	.003
		N	49	49	49
	MinO_HA	Correlation Coefficient	.176	.382**	1.000
		Sig. (1-tailed)	.113	.003	.
		N	49	49	49
	MinS_HA	Correlation Coefficient	.082	.146	.396**
		Sig. (1-tailed)	.289	.159	.002
		N	49	49	49

Correlations

			MinS_HA
Spearman's rho	Mood	Correlation Coefficient	.022
		Sig. (1-tailed)	.441
		N	49
	MajF_HA	Correlation Coefficient	.053
		Sig. (1-tailed)	.358
		N	49
	MajO_HA	Correlation Coefficient	-.072
		Sig. (1-tailed)	.311
		N	49
	MajS_HA	Correlation Coefficient	.082
		Sig. (1-tailed)	.289
		N	49
	MinF_HA	Correlation Coefficient	.146
		Sig. (1-tailed)	.159
		N	49
	MinO_HA	Correlation Coefficient	.396**
		Sig. (1-tailed)	.002
		N	49
	MinS_HA	Correlation Coefficient	1.000
		Sig. (1-tailed)	.
		N	49

**. Correlation is significant at the 0.01 level (1-tailed).

```
GLM MajF_positive MajO_positive MajS_positive MinF_positive MinO_positive M
inS_positive
  /WSFACTOR=Mode 2 Polynomial Tempo 3 Polynomial
  /METHOD=SSTYPE(3)
  /PLOT=PROFILE(Mode*Tempo Tempo*Mode) TYPE=LINE ERRORBAR=NO MEANREFERENCE=
NO YAXIS=AUTO
  /EMMEANS=TABLES(Mode) COMPARE ADJ(BONFERRONI)
  /EMMEANS=TABLES(Tempo) COMPARE ADJ(BONFERRONI)
  /EMMEANS=TABLES(Mode*Tempo)
  /PRINT=DESCRIPTIVE ETASQ HOMOGENEITY
  /CRITERIA=ALPHA(.05)
  /WSDESIGN=Mode Tempo Mode*Tempo.
```

General Linear Model

Notes

Output Created		24-MAR-2018 18:00...
Comments		
Input	Data	/Users/tayyulingrosabel/Desktop/FYP /Data to submit /Data files and analysis output/SPSS data.sav
	Active Dataset	DataSet1
	Filter	<none>
	Weight	<none>
	Split File	<none>
	N of Rows in Working Data File	52
Missing Value Handling	Definition of Missing	User-defined missing values are treated as missing.
	Cases Used	Statistics are based on all cases with valid data for all variables in the model.
Syntax		GLM MajF_positive MajO_positive MajS_positive MinF_positive MinO_positive MinS_positive<br>/WSFACTOR=Mode 2 Polynomial Tempo 3 Polynomial<br>/METHOD=SSTYPE(3)<br>/PLOT=PROFILE (Mode*Tempo Tempo*Mode) TYPE=LINE ERRORBAR=NO MEANREFERENCE=NO YAXIS=AUTO<br>/EMMEANS=TABLES (Mode) COMPARE ADJ (BONFERRONI)<br>/EMMEANS=TABLES (Tempo) COMPARE ADJ (BONFERRONI)<br>/EMMEANS=TABLES (Mode*Tempo)<br>/PRINT=DESCRIPTIVE ETASQ HOMOGENEITY<br>/CRITERIA=ALPHA(.05)<br>/WSDESIGN=Mode Tempo Mode*Tempo.
Resources	Processor Time	00:00:01.32
	Elapsed Time	00:00:01.00

Warnings

The HOMOGENEITY specification in the PRINT subcommand will be ignored because there are no between-subjects factors.

Within-Subjects Factors

Measure: MEASURE_1

Mode	Tempo	Dependent Variable
1	1	MajF_positive
	2	MajO_positive
	3	MajS_positive
2	1	MinF_positive
	2	MinO_positive
	3	MinS_positive

Descriptive Statistics

	Mean	Std. Deviation	N
MajF_positive	11.78	2.460	49
MajO_positive	12.45	2.623	49
MajS_positive	11.88	2.530	49
MinF_positive	6.22	3.280	49
MinO_positive	7.27	3.258	49
MinS_positive	6.37	3.365	49

Multivariate Tests[a]

Effect		Value	F	Hypothesis df	Error df
Mode	Pillai's Trace	.736	133.969 [b]	1.000	48.000
	Wilks' Lambda	.264	133.969 [b]	1.000	48.000
	Hotelling's Trace	2.791	133.969 [b]	1.000	48.000
	Roy's Largest Root	2.791	133.969 [b]	1.000	48.000
Tempo	Pillai's Trace	.133	3.590 [b]	2.000	47.000
	Wilks' Lambda	.867	3.590 [b]	2.000	47.000
	Hotelling's Trace	.153	3.590 [b]	2.000	47.000
	Roy's Largest Root	.153	3.590 [b]	2.000	47.000
Mode * Tempo	Pillai's Trace	.009	.221 [b]	2.000	47.000
	Wilks' Lambda	.991	.221 [b]	2.000	47.000
	Hotelling's Trace	.009	.221 [b]	2.000	47.000
	Roy's Largest Root	.009	.221 [b]	2.000	47.000

Multivariate Tests[a]

Effect		Sig.	Partial Eta Squared
Mode	Pillai's Trace	.000	.736
	Wilks' Lambda	.000	.736
	Hotelling's Trace	.000	.736
	Roy's Largest Root	.000	.736
Tempo	Pillai's Trace	.035	.133
	Wilks' Lambda	.035	.133
	Hotelling's Trace	.035	.133
	Roy's Largest Root	.035	.133
Mode * Tempo	Pillai's Trace	.803	.009
	Wilks' Lambda	.803	.009
	Hotelling's Trace	.803	.009
	Roy's Largest Root	.803	.009

a. Design: Intercept
Within Subjects Design: Mode + Tempo + Mode * Tempo

b. Exact statistic

Mauchly's Test of Sphericity[a]

Measure: MEASURE_1

Within Subjects Effect	Mauchly's W	Approx. Chi-Square	df	Sig.	Epsilon[b] Greenhouse-Geisser
Mode	1.000	.000	0	.	1.000
Tempo	.981	.881	2	.644	.982
Mode * Tempo	.998	.078	2	.962	.998

Mauchly's Test of Sphericity[a]

Measure: MEASURE_1

	Epsilon[b]	
Within Subjects Effect	Huynh-Feldt	Lower-bound
Mode	1.000	1.000
Tempo	1.000	.500
Mode * Tempo	1.000	.500

Tests the null hypothesis that the error covariance matrix of the orthonormalized transformed dependent variables is proportional to an identity matrix.

a. Design: Intercept
Within Subjects Design: Mode + Tempo + Mode * Tempo

b. May be used to adjust the degrees of freedom for the averaged tests of significance. Corrected tests are displayed in the Tests of Within-Subjects Effects table.

Tests of Within-Subjects Effects

Measure: MEASURE_1

Source		Type III Sum of Squares	df	Mean Square
Mode	Sphericity Assumed	2155.156	1	2155.156
	Greenhouse-Geisser	2155.156	1.000	2155.156
	Huynh-Feldt	2155.156	1.000	2155.156
	Lower-bound	2155.156	1.000	2155.156
Error(Mode)	Sphericity Assumed	772.177	48	16.087
	Greenhouse-Geisser	772.177	48.000	16.087
	Huynh-Feldt	772.177	48.000	16.087
	Lower-bound	772.177	48.000	16.087
Tempo	Sphericity Assumed	42.122	2	21.061
	Greenhouse-Geisser	42.122	1.964	21.452
	Huynh-Feldt	42.122	2.000	21.061
	Lower-bound	42.122	1.000	42.122
Error(Tempo)	Sphericity Assumed	626.211	96	6.523
	Greenhouse-Geisser	626.211	94.249	6.644
	Huynh-Feldt	626.211	96.000	6.523
	Lower-bound	626.211	48.000	13.046
Mode * Tempo	Sphericity Assumed	1.986	2	.993
	Greenhouse-Geisser	1.986	1.997	.995
	Huynh-Feldt	1.986	2.000	.993
	Lower-bound	1.986	1.000	1.986
Error(Mode*Tempo)	Sphericity Assumed	423.680	96	4.413
	Greenhouse-Geisser	423.680	95.841	4.421
	Huynh-Feldt	423.680	96.000	4.413
	Lower-bound	423.680	48.000	8.827

Tests of Within-Subjects Effects

Measure: MEASURE_1

Source		F	Sig.	Partial Eta Squared
Mode	Sphericity Assumed	133.969	.000	.736
	Greenhouse-Geisser	133.969	.000	.736
	Huynh-Feldt	133.969	.000	.736
	Lower-bound	133.969	.000	.736
Error(Mode)	Sphericity Assumed			
	Greenhouse-Geisser			
	Huynh-Feldt			
	Lower-bound			
Tempo	Sphericity Assumed	3.229	.044	.063
	Greenhouse-Geisser	3.229	.045	.063
	Huynh-Feldt	3.229	.044	.063
	Lower-bound	3.229	.079	.063
Error(Tempo)	Sphericity Assumed			
	Greenhouse-Geisser			
	Huynh-Feldt			
	Lower-bound			
Mode * Tempo	Sphericity Assumed	.225	.799	.005
	Greenhouse-Geisser	.225	.799	.005
	Huynh-Feldt	.225	.799	.005
	Lower-bound	.225	.637	.005
Error(Mode*Tempo)	Sphericity Assumed			
	Greenhouse-Geisser			
	Huynh-Feldt			
	Lower-bound			

Tests of Within-Subjects Contrasts

Measure: MEASURE_1

Source	Mode	Tempo	Type III Sum of Squares	df	Mean Square	F
Mode	Linear		2155.156	1	2155.156	133.969
Error(Mode)	Linear		772.177	48	16.087	
Tempo		Linear	.735	1	.735	.100
		Quadratic	41.388	1	41.388	7.292
Error(Tempo)		Linear	353.765	48	7.370	
		Quadratic	272.446	48	5.676	
Mode * Tempo	Linear	Linear	.020	1	.020	.005
		Quadratic	1.966	1	1.966	.449
Error(Mode*Tempo)	Linear	Linear	213.480	48	4.447	
		Quadratic	210.201	48	4.379	

Tests of Within-Subjects Contrasts

Measure: MEASURE_1

Source	Mode	Tempo	Sig.	Partial Eta Squared
Mode	Linear		.000	.736
Error(Mode)	Linear			
Tempo		Linear	.754	.002
		Quadratic	.010	.132
Error(Tempo)		Linear		
		Quadratic		
Mode * Tempo	Linear	Linear	.946	.000
		Quadratic	.506	.009
Error(Mode*Tempo)	Linear	Linear		
		Quadratic		

Tests of Between-Subjects Effects

Measure: MEASURE_1

Transformed Variable: Average

Source	Type III Sum of Squares	df	Mean Square	F	Sig.	Partial Eta Squared
Intercept	25573.347	1	25573.347	1817.688	.000	.974
Error	675.320	48	14.069			

Estimated Marginal Means

1. Mode

Estimates

Measure: MEASURE_1

Mode	Mean	Std. Error	95% Confidence Interval Lower Bound	95% Confidence Interval Upper Bound
1	12.034	.244	11.543	12.525
2	6.619	.382	5.852	7.386

Pairwise Comparisons

Measure: MEASURE_1

(I) Mode	(J) Mode	Mean Difference (I-J)	Std. Error	Sig.[b]	95% Confidence Interval for Difference[b]	
					Lower Bound	Upper Bound
1	2	5.415*	.468	.000	4.474	6.356
2	1	-5.415*	.468	.000	-6.356	-4.474

Based on estimated marginal means

*. The mean difference is significant at the .05 level.

b. Adjustment for multiple comparisons: Bonferroni.

Multivariate Tests

	Value	F	Hypothesis df	Error df	Sig.	Partial Eta Squared
Pillai's trace	.736	133.969[a]	1.000	48.000	.000	.736
Wilks' lambda	.264	133.969[a]	1.000	48.000	.000	.736
Hotelling's trace	2.791	133.969[a]	1.000	48.000	.000	.736
Roy's largest root	2.791	133.969[a]	1.000	48.000	.000	.736

Each F tests the multivariate effect of Mode. These tests are based on the linearly independent pairwise comparisons among the estimated marginal means.

a. Exact statistic

2. Tempo

Estimates

Measure: MEASURE_1

Tempo	Mean	Std. Error	95% Confidence Interval	
			Lower Bound	Upper Bound
1	9.000	.293	8.410	9.590
2	9.857	.288	9.278	10.436
3	9.122	.328	8.463	9.782

Pairwise Comparisons

Measure: MEASURE_1

(I) Tempo	(J) Tempo	Mean Difference (I-J)	Std. Error	Sig.[a]	95% Confidence Interval for Difference[a]	
					Lower Bound	Upper Bound
1	2	-.857	.360	.063	-1.749	.035
	3	-.122	.388	1.000	-1.085	.840
2	1	.857	.360	.063	-.035	1.749
	3	.735	.346	.117	-.124	1.593
3	1	.122	.388	1.000	-.840	1.085
	2	-.735	.346	.117	-1.593	.124

Based on estimated marginal means

a. Adjustment for multiple comparisons: Bonferroni.

Multivariate Tests

	Value	F	Hypothesis df	Error df	Sig.	Partial Eta Squared
Pillai's trace	.133	3.590[a]	2.000	47.000	.035	.133
Wilks' lambda	.867	3.590[a]	2.000	47.000	.035	.133
Hotelling's trace	.153	3.590[a]	2.000	47.000	.035	.133
Roy's largest root	.153	3.590[a]	2.000	47.000	.035	.133

Each F tests the multivariate effect of Tempo. These tests are based on the linearly independent pairwise comparisons among the estimated marginal means.

a. Exact statistic

3. Mode * Tempo

Measure: MEASURE_1

Mode	Tempo	Mean	Std. Error	95% Confidence Interval	
				Lower Bound	Upper Bound
1	1	11.776	.351	11.069	12.482
	2	12.449	.375	11.696	13.202
	3	11.878	.361	11.151	12.604
2	1	6.224	.469	5.282	7.167
	2	7.265	.465	6.329	8.201
	3	6.367	.481	5.401	7.334

Profile Plots

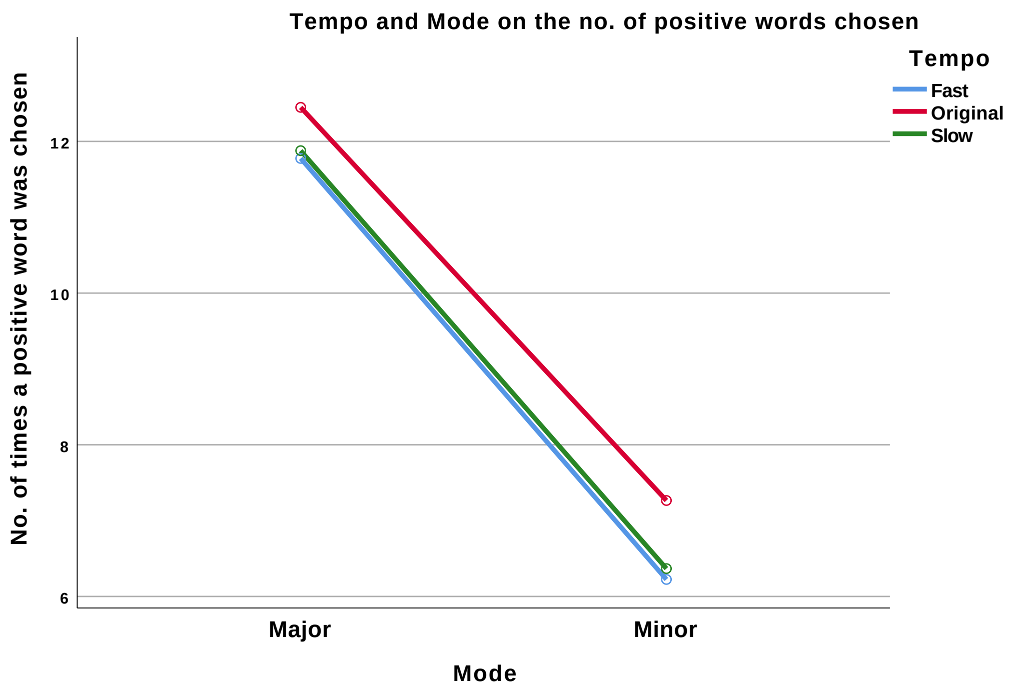

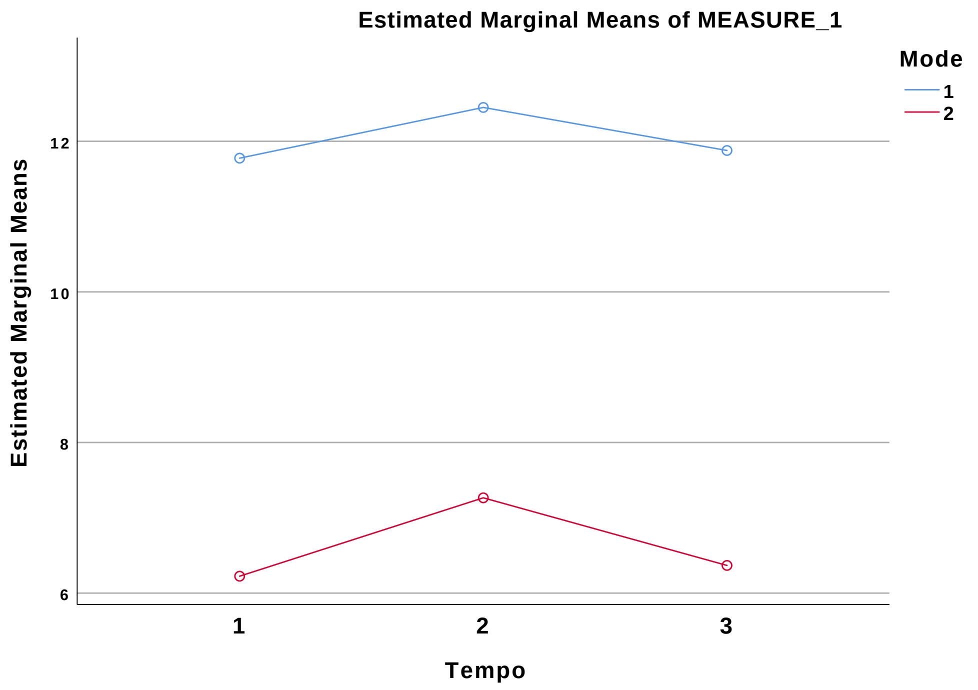


```
GLM MajF_HA MajO_HA MajS_HA MinF_HA MinO_HA MinS_HA
  /WSFACTOR=Mode 2 Polynomial Tempo 3 Polynomial
  /METHOD=SSTYPE(3)
  /PLOT=PROFILE(Mode*Tempo Tempo*Mode) TYPE=LINE ERRORBAR=NO MEANREFERENCE=
NO YAXIS=AUTO
  /EMMEANS=TABLES(Mode) COMPARE ADJ(BONFERRONI)
  /EMMEANS=TABLES(Tempo) COMPARE ADJ(BONFERRONI)
  /EMMEANS=TABLES(Mode*Tempo)
```

```
/PRINT=DESCRIPTIVE ETASQ HOMOGENEITY
/CRITERIA=ALPHA(.05)
/WSDESIGN=Mode Tempo Mode*Tempo.
```

General Linear Model

Notes

Output Created		24-MAR-2018 18:01...
Comments		
Input	Data	/Users/tayyulingrosabel/Desktop/FYP /Data to submit /Data files and analysis output/SPSS data.sav
	Active Dataset	DataSet1
	Filter	<none>
	Weight	<none>
	Split File	<none>
	N of Rows in Working Data File	52
Missing Value Handling	Definition of Missing	User-defined missing values are treated as missing.
	Cases Used	Statistics are based on all cases with valid data for all variables in the model.
Syntax		GLM MajF_HA MajO_HA MajS_HA MinF_HA MinO_HA MinS_HA /WSFACTOR=Mode 2 Polynomial Tempo 3 Polynomial /METHOD=SSTYPE(3) /PLOT=PROFILE (Mode*Tempo Tempo*Mode) TYPE=LINE ERRORBAR=NO MEANREFERENCE=NO YAXIS=AUTO /EMMEANS=TABLES (Mode) COMPARE ADJ (BONFERRONI) /EMMEANS=TABLES (Tempo) COMPARE ADJ (BONFERRONI) /EMMEANS=TABLES (Mode*Tempo) /PRINT=DESCRIPTIVE ETASQ HOMOGENEITY /CRITERIA=ALPHA(.05) /WSDESIGN=Mode Tempo Mode*Tempo.
Resources	Processor Time	00:00:00.55
	Elapsed Time	00:00:01.00

Warnings

The HOMOGENEITY specification in the PRINT subcommand will be ignored because there are no between-subjects factors.

Within-Subjects Factors

Measure: MEASURE_1

Mode	Tempo	Dependent Variable
1	1	MajF_HA
	2	MajO_HA
	3	MajS_HA
2	1	MinF_HA
	2	MinO_HA
	3	MinS_HA

Descriptive Statistics

	Mean	Std. Deviation	N
MajF_HA	6.29	2.965	49
MajO_HA	6.22	1.982	49
MajS_HA	3.57	2.264	49
MinF_HA	9.20	2.901	49
MinO_HA	6.43	2.638	49
MinS_HA	4.35	2.840	49

Multivariate Tests[a]

Effect		Value	F	Hypothesis df	Error df	Sig.
Mode	Pillai's Trace	.279	18.608[b]	1.000	48.000	.000
	Wilks' Lambda	.721	18.608[b]	1.000	48.000	.000
	Hotelling's Trace	.388	18.608[b]	1.000	48.000	.000
	Roy's Largest Root	.388	18.608[b]	1.000	48.000	.000
Tempo	Pillai's Trace	.647	43.050[b]	2.000	47.000	.000
	Wilks' Lambda	.353	43.050[b]	2.000	47.000	.000
	Hotelling's Trace	1.832	43.050[b]	2.000	47.000	.000
	Roy's Largest Root	1.832	43.050[b]	2.000	47.000	.000
Mode * Tempo	Pillai's Trace	.350	12.667[b]	2.000	47.000	.000
	Wilks' Lambda	.650	12.667[b]	2.000	47.000	.000
	Hotelling's Trace	.539	12.667[b]	2.000	47.000	.000
	Roy's Largest Root	.539	12.667[b]	2.000	47.000	.000

Multivariate Tests[a]

Effect		Partial Eta Squared
Mode	Pillai's Trace	.279
	Wilks' Lambda	.279
	Hotelling's Trace	.279
	Roy's Largest Root	.279
Tempo	Pillai's Trace	.647
	Wilks' Lambda	.647
	Hotelling's Trace	.647
	Roy's Largest Root	.647
Mode * Tempo	Pillai's Trace	.350
	Wilks' Lambda	.350
	Hotelling's Trace	.350
	Roy's Largest Root	.350

a. Design: Intercept
Within Subjects Design: Mode + Tempo + Mode * Tempo

b. Exact statistic

Mauchly's Test of Sphericity[a]

Measure: MEASURE_1

Within Subjects Effect	Mauchly's W	Approx. Chi-Square	df	Sig.	Epsilon[b] Greenhouse-Geisser
Mode	1.000	.000	0	.	1.000
Tempo	.786	11.302	2	.004	.824
Mode * Tempo	.988	.568	2	.753	.988

Mauchly's Test of Sphericity[a]

Measure: MEASURE_1

	Epsilon[b]	
Within Subjects Effect	Huynh-Feldt	Lower-bound
Mode	1.000	1.000
Tempo	.849	.500
Mode * Tempo	1.000	.500

Tests the null hypothesis that the error covariance matrix of the orthonormalized transformed dependent variables is proportional to an identity matrix.

a. Design: Intercept
Within Subjects Design: Mode + Tempo + Mode * Tempo

b. May be used to adjust the degrees of freedom for the averaged tests of significance. Corrected tests are displayed in the Tests of Within-Subjects Effects table.

Tests of Within-Subjects Effects

Measure: MEASURE_1

Source		Type III Sum of Squares	df	Mean Square	F
Mode	Sphericity Assumed	124.085	1	124.085	18.608
	Greenhouse-Geisser	124.085	1.000	124.085	18.608
	Huynh-Feldt	124.085	1.000	124.085	18.608
	Lower-bound	124.085	1.000	124.085	18.608
Error(Mode)	Sphericity Assumed	320.082	48	6.668	
	Greenhouse-Geisser	320.082	48.000	6.668	
	Huynh-Feldt	320.082	48.000	6.668	
	Lower-bound	320.082	48.000	6.668	
Tempo	Sphericity Assumed	716.959	2	358.480	54.135
	Greenhouse-Geisser	716.959	1.648	435.100	54.135
	Huynh-Feldt	716.959	1.699	422.043	54.135
	Lower-bound	716.959	1.000	716.959	54.135
Error(Tempo)	Sphericity Assumed	635.707	96	6.622	
	Greenhouse-Geisser	635.707	79.095	8.037	
	Huynh-Feldt	635.707	81.542	7.796	
	Lower-bound	635.707	48.000	13.244	
Mode * Tempo	Sphericity Assumed	100.333	2	50.167	12.775
	Greenhouse-Geisser	100.333	1.976	50.770	12.775
	Huynh-Feldt	100.333	2.000	50.167	12.775
	Lower-bound	100.333	1.000	100.333	12.775
Error(Mode*Tempo)	Sphericity Assumed	377.000	96	3.927	
	Greenhouse-Geisser	377.000	94.860	3.974	
	Huynh-Feldt	377.000	96.000	3.927	
	Lower-bound	377.000	48.000	7.854	

Tests of Within-Subjects Effects

Measure: MEASURE_1

Source		Sig.	Partial Eta Squared
Mode	Sphericity Assumed	.000	.279
	Greenhouse-Geisser	.000	.279
	Huynh-Feldt	.000	.279
	Lower-bound	.000	.279
Error(Mode)	Sphericity Assumed		
	Greenhouse-Geisser		
	Huynh-Feldt		
	Lower-bound		
Tempo	Sphericity Assumed	.000	.530
	Greenhouse-Geisser	.000	.530
	Huynh-Feldt	.000	.530
	Lower-bound	.000	.530
Error(Tempo)	Sphericity Assumed		
	Greenhouse-Geisser		
	Huynh-Feldt		
	Lower-bound		
Mode * Tempo	Sphericity Assumed	.000	.210
	Greenhouse-Geisser	.000	.210
	Huynh-Feldt	.000	.210
	Lower-bound	.001	.210
Error(Mode*Tempo)	Sphericity Assumed		
	Greenhouse-Geisser		
	Huynh-Feldt		
	Lower-bound		

Tests of Within-Subjects Contrasts

Measure: MEASURE_1

Source	Mode	Tempo	Type III Sum of Squares	df	Mean Square	F
Mode	Linear		124.085	1	124.085	18.608
Error(Mode)	Linear		320.082	48	6.668	
Tempo		Linear	702.250	1	702.250	74.247
		Quadratic	14.709	1	14.709	3.886
Error(Tempo)		Linear	454.000	48	9.458	
		Quadratic	181.707	48	3.786	
Mode * Tempo	Linear	Linear	56.250	1	56.250	12.919
		Quadratic	44.083	1	44.083	12.595
Error(Mode*Tempo)	Linear	Linear	209.000	48	4.354	
		Quadratic	168.000	48	3.500	

Tests of Within-Subjects Contrasts

Measure: MEASURE_1

Source	Mode	Tempo	Sig.	Partial Eta Squared
Mode	Linear		.000	.279
Error(Mode)	Linear			
Tempo		Linear	.000	.607
		Quadratic	.054	.075
Error(Tempo)		Linear		
		Quadratic		
Mode * Tempo	Linear	Linear	.001	.212
		Quadratic	.001	.208
Error(Mode*Tempo)	Linear	Linear		
		Quadratic		

Tests of Between-Subjects Effects

Measure: MEASURE_1

Transformed Variable: Average

Source	Type III Sum of Squares	df	Mean Square	F	Sig.	Partial Eta Squared
Intercept	10620.031	1	10620.031	785.696	.000	.942
Error	648.803	48	13.517			

Estimated Marginal Means

1. Mode

Estimates

Measure: MEASURE_1

Mode	Mean	Std. Error	95% Confidence Interval Lower Bound	95% Confidence Interval Upper Bound
1	5.361	.229	4.900	5.821
2	6.660	.291	6.074	7.246

Pairwise Comparisons

Measure: MEASURE_1

(I) Mode	(J) Mode	Mean Difference (I-J)	Std. Error	Sig.[b]	95% Confidence Interval for Difference[b] Lower Bound	Upper Bound
1	2	-1.299*	.301	.000	-1.905	-.694
2	1	1.299*	.301	.000	.694	1.905

Based on estimated marginal means

*. The mean difference is significant at the .05 level.

b. Adjustment for multiple comparisons: Bonferroni.

Multivariate Tests

	Value	F	Hypothesis df	Error df	Sig.	Partial Eta Squared
Pillai's trace	.279	18.608[a]	1.000	48.000	.000	.279
Wilks' lambda	.721	18.608[a]	1.000	48.000	.000	.279
Hotelling's trace	.388	18.608[a]	1.000	48.000	.000	.279
Roy's largest root	.388	18.608[a]	1.000	48.000	.000	.279

Each F tests the multivariate effect of Mode. These tests are based on the linearly independent pairwise comparisons among the estimated marginal means.

a. Exact statistic

2. Tempo

Estimates

Measure: MEASURE_1

Tempo	Mean	Std. Error	95% Confidence Interval Lower Bound	Upper Bound
1	7.745	.358	7.026	8.464
2	6.327	.262	5.799	6.854
3	3.959	.276	3.404	4.515

Pairwise Comparisons

Measure: MEASURE_1

(I) Tempo	(J) Tempo	Mean Difference (I-J)	Std. Error	Sig.[b]	95% Confidence Interval for Difference[b]	
					Lower Bound	Upper Bound
1	2	1.418*	.356	.001	.536	2.301
	3	3.786*	.439	.000	2.696	4.876
2	1	-1.418*	.356	.001	-2.301	-.536
	3	2.367*	.293	.000	1.641	3.094
3	1	-3.786*	.439	.000	-4.876	-2.696
	2	-2.367*	.293	.000	-3.094	-1.641

Based on estimated marginal means

*. The mean difference is significant at the .05 level.

b. Adjustment for multiple comparisons: Bonferroni.

Multivariate Tests

	Value	F	Hypothesis df	Error df	Sig.	Partial Eta Squared
Pillai's trace	.647	43.050[a]	2.000	47.000	.000	.647
Wilks' lambda	.353	43.050[a]	2.000	47.000	.000	.647
Hotelling's trace	1.832	43.050[a]	2.000	47.000	.000	.647
Roy's largest root	1.832	43.050[a]	2.000	47.000	.000	.647

Each F tests the multivariate effect of Tempo. These tests are based on the linearly independent pairwise comparisons among the estimated marginal means.

a. Exact statistic

3. Mode * Tempo

Measure: MEASURE_1

Mode	Tempo	Mean	Std. Error	95% Confidence Interval	
				Lower Bound	Upper Bound
1	1	6.286	.424	5.434	7.137
	2	6.224	.283	5.655	6.794
	3	3.571	.323	2.921	4.222
2	1	9.204	.414	8.371	10.037
	2	6.429	.377	5.671	7.186
	3	4.347	.406	3.531	5.163

Profile Plots

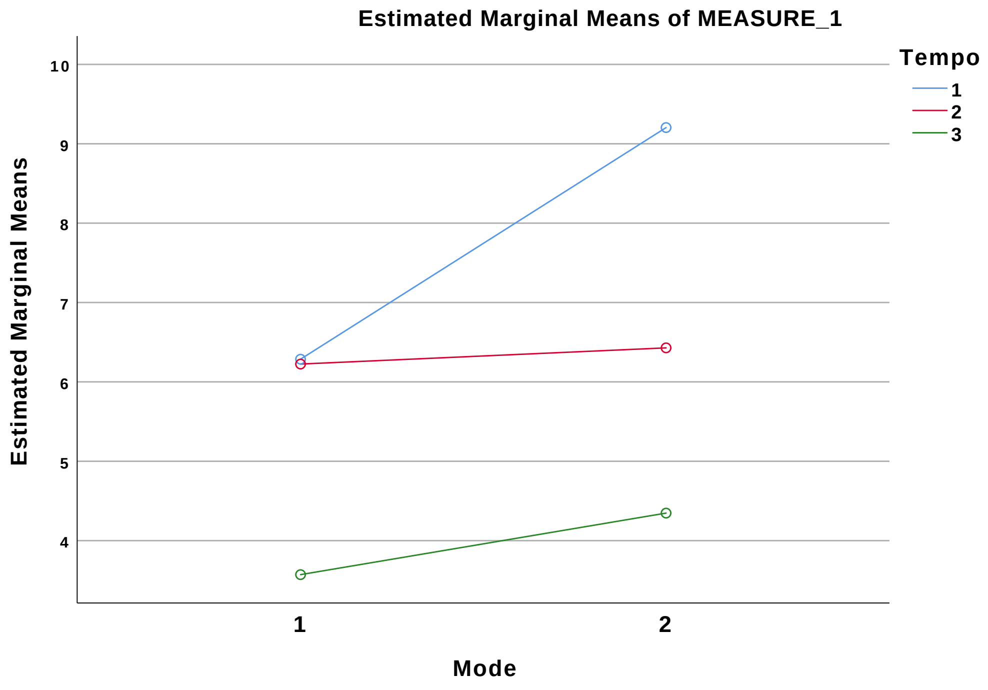

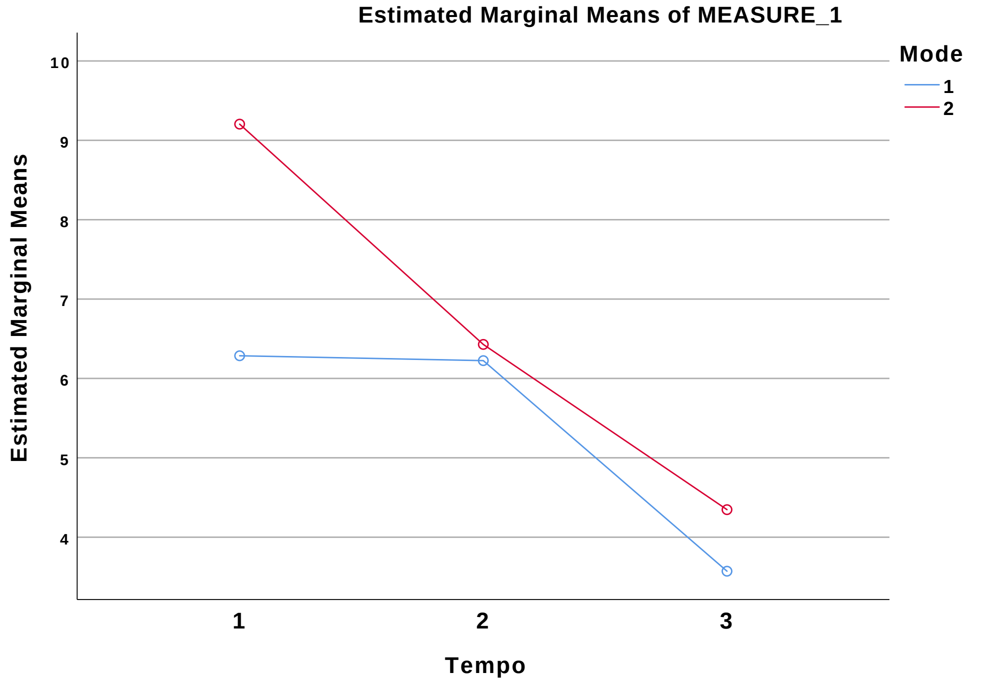


```
GLM Avg_intensitiy_MajF_positive Avg_intensitiy_MajO_positive Avg_intensiti
y_MajS_positive
    Avg_intensitiy_MinF_positive Avg_intensitiy_MinO_positive Avg_intensiti
y_MinS_positive
  /WSFACTOR=Mode 2 Polynomial Tempo 3 Polynomial
  /METHOD=SSTYPE(3)
  /PLOT=PROFILE(Mode*Tempo Tempo*Mode) TYPE=LINE ERRORBAR=NO MEANREFERENCE=
NO YAXIS=AUTO
```

```
/EMMEANS=TABLES(Mode) COMPARE ADJ(BONFERRONI)
/EMMEANS=TABLES(Tempo) COMPARE ADJ(BONFERRONI)
/EMMEANS=TABLES(Mode*Tempo)
/PRINT=DESCRIPTIVE ETASQ HOMOGENEITY
/CRITERIA=ALPHA(.05)
/WSDESIGN=Mode Tempo Mode*Tempo.
```

General Linear Model

Notes

Output Created		24-MAR-2018 18:01...
Comments		
Input	Data	/Users/tayyulingrosabel/Desktop/FYP /Data to submit /Data files and analysis output/SPSS data.sav
	Active Dataset	DataSet1
	Filter	<none>
	Weight	<none>
	Split File	<none>
	N of Rows in Working Data File	52
Missing Value Handling	Definition of Missing	User-defined missing values are treated as missing.
	Cases Used	Statistics are based on all cases with valid data for all variables in the model.

Notes

Syntax		GLM Avg_intensitiy_MajF_positive Avg_intensitiy_MajO_positive Avg_intensitiy_MajS_positive<br><br>Avg_intensitiy_MinF_positive Avg_intensitiy_MinO_positive Avg_intensitiy_MinS_positive<br>/WSFACTOR=Mode 2 Polynomial Tempo 3 Polynomial<br>/METHOD=SSTYPE(3)<br>/PLOT=PROFILE (Mode*Tempo Tempo*Mode) TYPE=LINE ERRORBAR=NO MEANREFERENCE=NO YAXIS=AUTO<br>/EMMEANS=TABLES (Mode) COMPARE ADJ (BONFERRONI)<br>/EMMEANS=TABLES (Tempo) COMPARE ADJ (BONFERRONI)<br>/EMMEANS=TABLES (Mode*Tempo)<br>/PRINT=DESCRIPTIVE ETASQ HOMOGENEITY<br>/CRITERIA=ALPHA(.05)<br>/WSDESIGN=Mode Tempo Mode*Tempo.
Resources	Processor Time	00:00:00.46
	Elapsed Time	00:00:01.00

Warnings

The HOMOGENEITY specification in the PRINT subcommand will be ignored because there are no between-subjects factors.

Within-Subjects Factors

Measure: MEASURE_1

Mode	Tempo	Dependent Variable
1	1	Avg_intensitiy_MajF_positive
	2	Avg_intensitiy_MajO_positive
	3	Avg_intensitiy_MajS_positive
2	1	Avg_intensitiy_MinF_positive
	2	Avg_intensitiy_MinO_positive
	3	Avg_intensitiy_MinS_positive

Descriptive Statistics

	Mean	Std. Deviation	N
Avg_intensitiy_MajF_positive	5.0259	.91723	49
Avg_intensitiy_MajO_positive	4.9591	.88266	49
Avg_intensitiy_MajS_positive	4.9034	.79914	49
Avg_intensitiy_MinF_positive	4.3736	1.38029	49
Avg_intensitiy_MinO_positive	4.7113	.88369	49
Avg_intensitiy_MinS_positive	4.4518	1.31589	49

Multivariate Tests[a]

Effect		Value	F	Hypothesis df	Error df	Sig.
Mode	Pillai's Trace	.327	23.291[b]	1.000	48.000	.000
	Wilks' Lambda	.673	23.291[b]	1.000	48.000	.000
	Hotelling's Trace	.485	23.291[b]	1.000	48.000	.000
	Roy's Largest Root	.485	23.291[b]	1.000	48.000	.000
Tempo	Pillai's Trace	.086	2.210[b]	2.000	47.000	.121
	Wilks' Lambda	.914	2.210[b]	2.000	47.000	.121
	Hotelling's Trace	.094	2.210[b]	2.000	47.000	.121
	Roy's Largest Root	.094	2.210[b]	2.000	47.000	.121
Mode * Tempo	Pillai's Trace	.083	2.121[b]	2.000	47.000	.131
	Wilks' Lambda	.917	2.121[b]	2.000	47.000	.131
	Hotelling's Trace	.090	2.121[b]	2.000	47.000	.131
	Roy's Largest Root	.090	2.121[b]	2.000	47.000	.131

Multivariate Tests[a]

Effect		Partial Eta Squared
Mode	Pillai's Trace	.327
	Wilks' Lambda	.327
	Hotelling's Trace	.327
	Roy's Largest Root	.327
Tempo	Pillai's Trace	.086
	Wilks' Lambda	.086
	Hotelling's Trace	.086
	Roy's Largest Root	.086
Mode * Tempo	Pillai's Trace	.083
	Wilks' Lambda	.083
	Hotelling's Trace	.083
	Roy's Largest Root	.083

a. Design: Intercept
Within Subjects Design: Mode + Tempo + Mode * Tempo

b. Exact statistic

Mauchly's Test of Sphericity[a]

Measure: MEASURE_1

Within Subjects Effect	Mauchly's W	Approx. Chi-Square	df	Sig.	Epsilon[b] Greenhouse-Geisser
Mode	1.000	.000	0	.	1.000
Tempo	.713	15.890	2	.000	.777
Mode * Tempo	.904	4.761	2	.093	.912

Mauchly's Test of Sphericity[a]

Measure: MEASURE_1

	Epsilon[b]	
Within Subjects Effect	Huynh-Feldt	Lower-bound
Mode	1.000	1.000
Tempo	.798	.500
Mode * Tempo	.946	.500

Tests the null hypothesis that the error covariance matrix of the orthonormalized transformed dependent variables is proportional to an identity matrix.

a. Design: Intercept
Within Subjects Design: Mode + Tempo + Mode * Tempo

b. May be used to adjust the degrees of freedom for the averaged tests of significance. Corrected tests are displayed in the Tests of Within-Subjects Effects table.

Tests of Within-Subjects Effects

Measure: MEASURE_1

Source		Type III Sum of Squares	df	Mean Square	F
Mode	Sphericity Assumed	14.921	1	14.921	23.291
	Greenhouse-Geisser	14.921	1.000	14.921	23.291
	Huynh-Feldt	14.921	1.000	14.921	23.291
	Lower-bound	14.921	1.000	14.921	23.291
Error(Mode)	Sphericity Assumed	30.752	48	.641	
	Greenhouse-Geisser	30.752	48.000	.641	
	Huynh-Feldt	30.752	48.000	.641	
	Lower-bound	30.752	48.000	.641	
Tempo	Sphericity Assumed	1.428	2	.714	1.093
	Greenhouse-Geisser	1.428	1.554	.919	1.093
	Huynh-Feldt	1.428	1.597	.894	1.093
	Lower-bound	1.428	1.000	1.428	1.093
Error(Tempo)	Sphericity Assumed	62.694	96	.653	
	Greenhouse-Geisser	62.694	74.600	.840	
	Huynh-Feldt	62.694	76.636	.818	
	Lower-bound	62.694	48.000	1.306	
Mode * Tempo	Sphericity Assumed	2.005	2	1.002	1.605
	Greenhouse-Geisser	2.005	1.824	1.099	1.605
	Huynh-Feldt	2.005	1.893	1.059	1.605
	Lower-bound	2.005	1.000	2.005	1.605
Error(Mode*Tempo)	Sphericity Assumed	59.941	96	.624	
	Greenhouse-Geisser	59.941	87.565	.685	
	Huynh-Feldt	59.941	90.841	.660	
	Lower-bound	59.941	48.000	1.249	

Tests of Within-Subjects Effects

Measure: MEASURE_1

Source		Sig.	Partial Eta Squared
Mode	Sphericity Assumed	.000	.327
	Greenhouse-Geisser	.000	.327
	Huynh-Feldt	.000	.327
	Lower-bound	.000	.327
Error(Mode)	Sphericity Assumed		
	Greenhouse-Geisser		
	Huynh-Feldt		
	Lower-bound		
Tempo	Sphericity Assumed	.339	.022
	Greenhouse-Geisser	.327	.022
	Huynh-Feldt	.329	.022
	Lower-bound	.301	.022
Error(Tempo)	Sphericity Assumed		
	Greenhouse-Geisser		
	Huynh-Feldt		
	Lower-bound		
Mode * Tempo	Sphericity Assumed	.206	.032
	Greenhouse-Geisser	.208	.032
	Huynh-Feldt	.208	.032
	Lower-bound	.211	.032
Error(Mode*Tempo)	Sphericity Assumed		
	Greenhouse-Geisser		
	Huynh-Feldt		
	Lower-bound		

Tests of Within-Subjects Contrasts

Measure: MEASURE_1

Source	Mode	Tempo	Type III Sum of Squares	df	Mean Square	F
Mode	Linear		14.921	1	14.921	23.291
Error(Mode)	Linear		30.752	48	.641	
Tempo		Linear	.024	1	.024	.024
		Quadratic	1.404	1	1.404	4.493
Error(Tempo)		Linear	47.701	48	.994	
		Quadratic	14.994	48	.312	
Mode * Tempo	Linear	Linear	.494	1	.494	.608
		Quadratic	1.511	1	1.511	3.460
Error(Mode*Tempo)	Linear	Linear	38.978	48	.812	
		Quadratic	20.963	48	.437	

Tests of Within-Subjects Contrasts

Measure: MEASURE_1

Source	Mode	Tempo	Sig.	Partial Eta Squared
Mode	Linear		.000	.327
Error(Mode)	Linear			
Tempo		Linear	.877	.001
		Quadratic	.039	.086
Error(Tempo)		Linear		
		Quadratic		
Mode * Tempo	Linear	Linear	.439	.013
		Quadratic	.069	.067
Error(Mode*Tempo)	Linear	Linear		
		Quadratic		

Tests of Between-Subjects Effects

Measure: MEASURE_1
Transformed Variable: Average

Source	Type III Sum of Squares	df	Mean Square	F	Sig.	Partial Eta Squared
Intercept	6598.542	1	6598.542	1895.521	.000	.975
Error	167.094	48	3.481			

Estimated Marginal Means

1. Mode

Estimates

Measure: MEASURE_1

Mode	Mean	Std. Error	95% Confidence Interval	
			Lower Bound	Upper Bound
1	4.963	.116	4.730	5.195
2	4.512	.121	4.269	4.756

Pairwise Comparisons

Measure: MEASURE_1

(I) Mode	(J) Mode	Mean Difference (I-J)	Std. Error	Sig.[b]	95% Confidence Interval for Difference[b]	
					Lower Bound	Upper Bound
1	2	.451[*]	.093	.000	.263	.638
2	1	-.451[*]	.093	.000	-.638	-.263

Based on estimated marginal means

*. The mean difference is significant at the .05 level.

b. Adjustment for multiple comparisons: Bonferroni.

Multivariate Tests

	Value	F	Hypothesis df	Error df	Sig.	Partial Eta Squared
Pillai's trace	.327	23.291[a]	1.000	48.000	.000	.327
Wilks' lambda	.673	23.291[a]	1.000	48.000	.000	.327
Hotelling's trace	.485	23.291[a]	1.000	48.000	.000	.327
Roy's largest root	.485	23.291[a]	1.000	48.000	.000	.327

Each F tests the multivariate effect of Mode. These tests are based on the linearly independent pairwise comparisons among the estimated marginal means.

a. Exact statistic

2. Tempo

Estimates

Measure: MEASURE_1

Tempo	Mean	Std. Error	95% Confidence Interval	
			Lower Bound	Upper Bound
1	4.700	.140	4.419	4.981
2	4.835	.115	4.604	5.067
3	4.678	.127	4.423	4.932

Pairwise Comparisons

Measure: MEASURE_1

(I) Tempo	(J) Tempo	Mean Difference (I-J)	Std. Error	Sig.[a]	95% Confidence Interval for Difference[a]	
					Lower Bound	Upper Bound
1	2	-.135	.092	.441	-.364	.093
	3	.022	.142	1.000	-.331	.375
2	1	.135	.092	.441	-.093	.364
	3	.158	.106	.431	-.105	.421
3	1	-.022	.142	1.000	-.375	.331
	2	-.158	.106	.431	-.421	.105

Based on estimated marginal means

a. Adjustment for multiple comparisons: Bonferroni.

Multivariate Tests

	Value	F	Hypothesis df	Error df	Sig.	Partial Eta Squared
Pillai's trace	.086	2.210[a]	2.000	47.000	.121	.086
Wilks' lambda	.914	2.210[a]	2.000	47.000	.121	.086
Hotelling's trace	.094	2.210[a]	2.000	47.000	.121	.086
Roy's largest root	.094	2.210[a]	2.000	47.000	.121	.086

Each F tests the multivariate effect of Tempo. These tests are based on the linearly independent pairwise comparisons among the estimated marginal means.

a. Exact statistic

3. Mode * Tempo

Measure: MEASURE_1

Mode	Tempo	Mean	Std. Error	95% Confidence Interval	
				Lower Bound	Upper Bound
1	1	5.026	.131	4.762	5.289
	2	4.959	.126	4.706	5.213
	3	4.903	.114	4.674	5.133
2	1	4.374	.197	3.977	4.770
	2	4.711	.126	4.458	4.965
	3	4.452	.188	4.074	4.830

Profile Plots

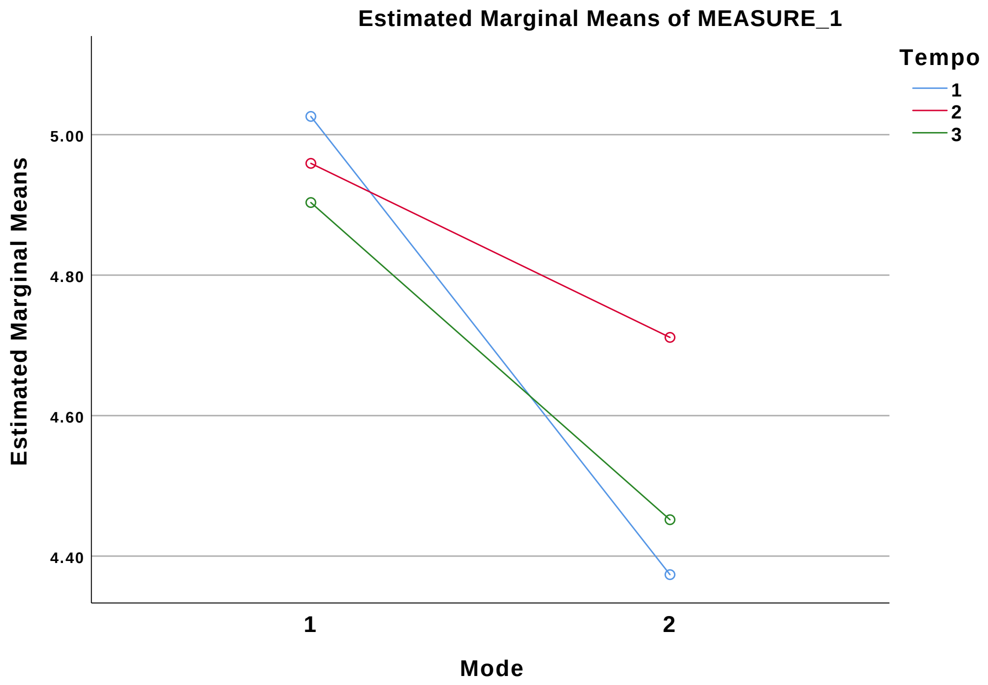

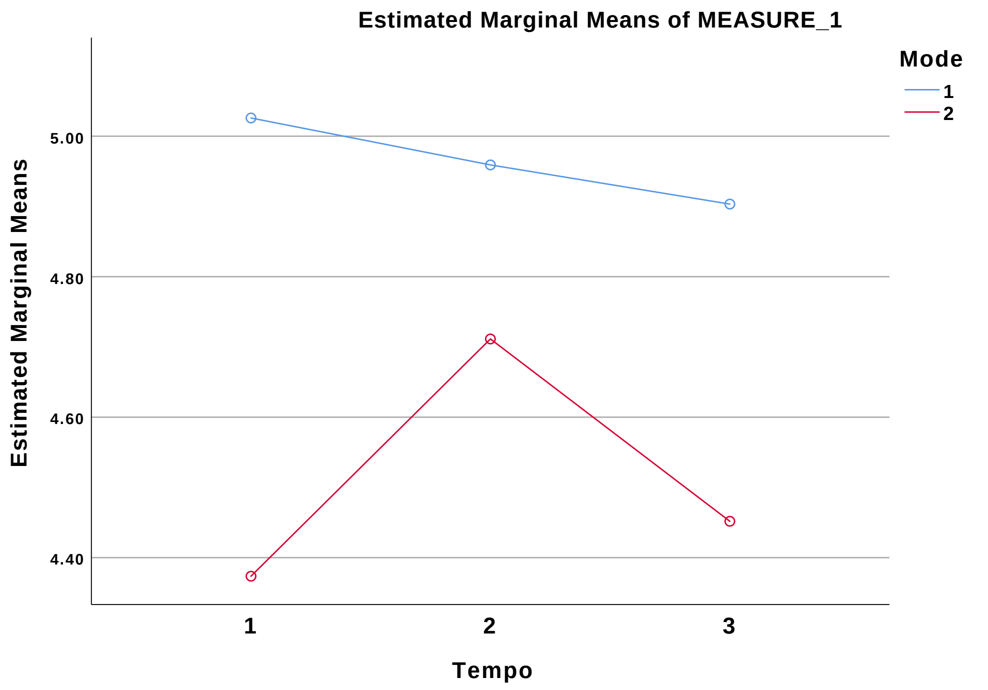


```
GLM Avg_intensitiy_MajF_HA Avg_intensitiy_MajO_HA Avg_intensitiy_MajS_HA Av
g_intensitiy_MinF_HA
    Avg_intensitiy_MinO_HA Avg_intensitiy_MinS_HA
  /WSFACTOR=Mode 2 Polynomial Tempo 3 Polynomial
  /METHOD=SSTYPE(3)
  /PLOT=PROFILE(Mode*Tempo Tempo*Mode) TYPE=LINE ERRORBAR=NO MEANREFERENCE=
NO YAXIS=AUTO
  /EMMEANS=TABLES(Mode) COMPARE ADJ(BONFERRONI)
```

```
/EMMEANS=TABLES(Tempo) COMPARE ADJ(BONFERRONI)
/EMMEANS=TABLES(Mode*Tempo)
/PRINT=DESCRIPTIVE ETASQ HOMOGENEITY
/CRITERIA=ALPHA(.05)
/WSDESIGN=Mode Tempo Mode*Tempo.
```

General Linear Model

Notes

Output Created		24-MAR-2018 18:05...
Comments		
Input	Data	/Users/tayyulingrosabel/Desktop/FYP /Data to submit /Data files and analysis output/SPSS data.sav
	Active Dataset	DataSet1
	Filter	<none>
	Weight	<none>
	Split File	<none>
	N of Rows in Working Data File	52
Missing Value Handling	Definition of Missing	User-defined missing values are treated as missing.
	Cases Used	Statistics are based on all cases with valid data for all variables in the model.

Notes

Syntax		GLM Avg_intensitiy_MajF_HA Avg_intensitiy_MajO_HA Avg_intensitiy_MajS_HA Avg_intensitiy_MinF_HA<br><br>Avg_intensitiy_MinO_HA Avg_intensitiy_MinS_HA<br>/WSFACTOR=Mode 2 Polynomial Tempo 3 Polynomial<br>/METHOD=SSTYPE(3)<br>/PLOT=PROFILE (Mode*Tempo Tempo*Mode) TYPE=LINE ERRORBAR=NO MEANREFERENCE=NO YAXIS=AUTO<br>/EMMEANS=TABLES (Mode) COMPARE ADJ (BONFERRONI)<br>/EMMEANS=TABLES (Tempo) COMPARE ADJ (BONFERRONI)<br>/EMMEANS=TABLES (Mode*Tempo)<br>/PRINT=DESCRIPTIVE ETASQ HOMOGENEITY<br>/CRITERIA=ALPHA(.05)<br>/WSDESIGN=Mode Tempo Mode*Tempo.
Resources	Processor Time	00:00:00.39
	Elapsed Time	00:00:00.00

Warnings

The HOMOGENEITY specification in the PRINT subcommand will be ignored because there are no between-subjects factors.

Within-Subjects Factors

Measure: MEASURE_1

Mode	Tempo	Dependent Variable
1	1	Avg_intensitiy_MajF_HA
	2	Avg_intensitiy_MajO_HA
	3	Avg_intensitiy_MajS_HA
2	1	Avg_intensitiy_MinF_HA
	2	Avg_intensitiy_MinO_HA
	3	Avg_intensitiy_MinS_HA

Descriptive Statistics

	Mean	Std. Deviation	N
Avg_intensitiy_MajF_HA	4.8240	1.43214	49
Avg_intensitiy_MajO_HA	4.8038	.90023	49
Avg_intensitiy_MajS_HA	4.4684	1.67383	49
Avg_intensitiy_MinF_HA	4.7003	.89883	49
Avg_intensitiy_MinO_HA	4.7391	.83757	49
Avg_intensitiy_MinS_HA	4.2261	1.53817	49

Multivariate Tests[a]

Effect		Value	F	Hypothesis df	Error df	Sig.
Mode	Pillai's Trace	.044	2.206[b]	1.000	48.000	.144
	Wilks' Lambda	.956	2.206[b]	1.000	48.000	.144
	Hotelling's Trace	.046	2.206[b]	1.000	48.000	.144
	Roy's Largest Root	.046	2.206[b]	1.000	48.000	.144
Tempo	Pillai's Trace	.103	2.696[b]	2.000	47.000	.078
	Wilks' Lambda	.897	2.696[b]	2.000	47.000	.078
	Hotelling's Trace	.115	2.696[b]	2.000	47.000	.078
	Roy's Largest Root	.115	2.696[b]	2.000	47.000	.078
Mode * Tempo	Pillai's Trace	.014	.328[b]	2.000	47.000	.722
	Wilks' Lambda	.986	.328[b]	2.000	47.000	.722
	Hotelling's Trace	.014	.328[b]	2.000	47.000	.722
	Roy's Largest Root	.014	.328[b]	2.000	47.000	.722

Multivariate Tests[a]

Effect		Partial Eta Squared
Mode	Pillai's Trace	.044
	Wilks' Lambda	.044
	Hotelling's Trace	.044
	Roy's Largest Root	.044
Tempo	Pillai's Trace	.103
	Wilks' Lambda	.103
	Hotelling's Trace	.103
	Roy's Largest Root	.103
Mode * Tempo	Pillai's Trace	.014
	Wilks' Lambda	.014
	Hotelling's Trace	.014
	Roy's Largest Root	.014

a. Design: Intercept
Within Subjects Design: Mode + Tempo + Mode * Tempo

b. Exact statistic

Mauchly's Test of Sphericity[a]

Measure: MEASURE_1

Within Subjects Effect	Mauchly's W	Approx. Chi-Square	df	Sig.	Epsilon[b] Greenhouse-Geisser
Mode	1.000	.000	0	.	1.000
Tempo	.689	17.486	2	.000	.763
Mode * Tempo	.896	5.158	2	.076	.906

Mauchly's Test of Sphericity[a]

Measure: MEASURE_1

	Epsilon[b]	
Within Subjects Effect	Huynh-Feldt	Lower-bound
Mode	1.000	1.000
Tempo	.783	.500
Mode * Tempo	.939	.500

Tests the null hypothesis that the error covariance matrix of the orthonormalized transformed dependent variables is proportional to an identity matrix.

a. Design: Intercept
Within Subjects Design: Mode + Tempo + Mode * Tempo

b. May be used to adjust the degrees of freedom for the averaged tests of significance. Corrected tests are displayed in the Tests of Within-Subjects Effects table.

Tests of Within-Subjects Effects

Measure: MEASURE_1

Source		Type III Sum of Squares	df	Mean Square	F
Mode	Sphericity Assumed	1.516	1	1.516	2.206
	Greenhouse-Geisser	1.516	1.000	1.516	2.206
	Huynh-Feldt	1.516	1.000	1.516	2.206
	Lower-bound	1.516	1.000	1.516	2.206
Error(Mode)	Sphericity Assumed	32.975	48	.687	
	Greenhouse-Geisser	32.975	48.000	.687	
	Huynh-Feldt	32.975	48.000	.687	
	Lower-bound	32.975	48.000	.687	
Tempo	Sphericity Assumed	11.507	2	5.754	4.260
	Greenhouse-Geisser	11.507	1.526	7.541	4.260
	Huynh-Feldt	11.507	1.566	7.349	4.260
	Lower-bound	11.507	1.000	11.507	4.260
Error(Tempo)	Sphericity Assumed	129.662	96	1.351	
	Greenhouse-Geisser	129.662	73.244	1.770	
	Huynh-Feldt	129.662	75.160	1.725	
	Lower-bound	129.662	48.000	2.701	
Mode * Tempo	Sphericity Assumed	.401	2	.200	.337
	Greenhouse-Geisser	.401	1.812	.221	.337
	Huynh-Feldt	.401	1.879	.213	.337
	Lower-bound	.401	1.000	.401	.337
Error(Mode*Tempo)	Sphericity Assumed	57.008	96	.594	
	Greenhouse-Geisser	57.008	86.962	.656	
	Huynh-Feldt	57.008	90.178	.632	
	Lower-bound	57.008	48.000	1.188	

Tests of Within-Subjects Effects

Measure: MEASURE_1

Source		Sig.	Partial Eta Squared
Mode	Sphericity Assumed	.144	.044
	Greenhouse-Geisser	.144	.044
	Huynh-Feldt	.144	.044
	Lower-bound	.144	.044
Error(Mode)	Sphericity Assumed		
	Greenhouse-Geisser		
	Huynh-Feldt		
	Lower-bound		
Tempo	Sphericity Assumed	.017	.082
	Greenhouse-Geisser	.027	.082
	Huynh-Feldt	.026	.082
	Lower-bound	.044	.082
Error(Tempo)	Sphericity Assumed		
	Greenhouse-Geisser		
	Huynh-Feldt		
	Lower-bound		
Mode * Tempo	Sphericity Assumed	.715	.007
	Greenhouse-Geisser	.693	.007
	Huynh-Feldt	.701	.007
	Lower-bound	.564	.007
Error(Mode*Tempo)	Sphericity Assumed		
	Greenhouse-Geisser		
	Huynh-Feldt		
	Lower-bound		

Tests of Within-Subjects Contrasts

Measure: MEASURE_1

Source	Mode	Tempo	Type III Sum of Squares	df	Mean Square	F
Mode	Linear		1.516	1	1.516	2.206
Error(Mode)	Linear		32.975	48	.687	
Tempo		Linear	8.438	1	8.438	4.771
		Quadratic	3.070	1	3.070	3.291
Error(Tempo)		Linear	84.891	48	1.769	
		Quadratic	44.771	48	.933	
Mode * Tempo	Linear	Linear	.172	1	.172	.226
		Quadratic	.228	1	.228	.538
Error(Mode*Tempo)	Linear	Linear	36.622	48	.763	
		Quadratic	20.387	48	.425	

Tests of Within-Subjects Contrasts

Measure: MEASURE_1

Source	Mode	Tempo	Sig.	Partial Eta Squared
Mode	Linear		.144	.044
Error(Mode)	Linear			
Tempo		Linear	.034	.090
		Quadratic	.076	.064
Error(Tempo)		Linear		
		Quadratic		
Mode * Tempo	Linear	Linear	.637	.005
		Quadratic	.467	.011
Error(Mode*Tempo)	Linear	Linear		
		Quadratic		

Tests of Between-Subjects Effects

Measure: MEASURE_1

Transformed Variable: Average

Source	Type III Sum of Squares	df	Mean Square	F	Sig.	Partial Eta Squared
Intercept	6294.133	1	6294.133	1268.313	.000	.964
Error	238.205	48	4.963			

Estimated Marginal Means

1. Mode

Estimates

Measure: MEASURE_1

Mode	Mean	Std. Error	95% Confidence Interval	
			Lower Bound	Upper Bound
1	4.699	.149	4.399	4.998
2	4.555	.127	4.299	4.811

Pairwise Comparisons

Measure: MEASURE_1

(I) Mode	(J) Mode	Mean Difference (I-J)	Std. Error	Sig.[a]	95% Confidence Interval for Difference[a]	
					Lower Bound	Upper Bound
1	2	.144	.097	.144	-.051	.338
2	1	-.144	.097	.144	-.338	.051

Based on estimated marginal means

a. Adjustment for multiple comparisons: Bonferroni.

Multivariate Tests

	Value	F	Hypothesis df	Error df	Sig.	Partial Eta Squared
Pillai's trace	.044	2.206[a]	1.000	48.000	.144	.044
Wilks' lambda	.956	2.206[a]	1.000	48.000	.144	.044
Hotelling's trace	.046	2.206[a]	1.000	48.000	.144	.044
Roy's largest root	.046	2.206[a]	1.000	48.000	.144	.044

Each F tests the multivariate effect of Mode. These tests are based on the linearly independent pairwise comparisons among the estimated marginal means.

a. Exact statistic

2. Tempo

Estimates

Measure: MEASURE_1

Tempo	Mean	Std. Error	95% Confidence Interval Lower Bound	95% Confidence Interval Upper Bound
1	4.762	.148	4.464	5.060
2	4.771	.113	4.544	4.999
3	4.347	.209	3.928	4.767

Pairwise Comparisons

Measure: MEASURE_1

(I) Tempo	(J) Tempo	Mean Difference (I-J)	Std. Error	Sig.[a]	95% Confidence Interval for Difference[a] Lower Bound	95% Confidence Interval for Difference[a] Upper Bound
1	2	-.009	.111	1.000	-.284	.265
	3	.415	.190	.102	-.056	.886
2	1	.009	.111	1.000	-.265	.284
	3	.424	.185	.080	-.036	.884
3	1	-.415	.190	.102	-.886	.056
	2	-.424	.185	.080	-.884	.036

Based on estimated marginal means

a. Adjustment for multiple comparisons: Bonferroni.

Multivariate Tests

	Value	F	Hypothesis df	Error df	Sig.	Partial Eta Squared
Pillai's trace	.103	2.696[a]	2.000	47.000	.078	.103
Wilks' lambda	.897	2.696[a]	2.000	47.000	.078	.103
Hotelling's trace	.115	2.696[a]	2.000	47.000	.078	.103
Roy's largest root	.115	2.696[a]	2.000	47.000	.078	.103

Each F tests the multivariate effect of Tempo. These tests are based on the linearly independent pairwise comparisons among the estimated marginal means.

a. Exact statistic

3. Mode * Tempo

Measure: MEASURE_1

Mode	Tempo	Mean	Std. Error	95% Confidence Interval Lower Bound	95% Confidence Interval Upper Bound
1	1	4.824	.205	4.413	5.235
	2	4.804	.129	4.545	5.062
	3	4.468	.239	3.988	4.949
2	1	4.700	.128	4.442	4.958
	2	4.739	.120	4.498	4.980
	3	4.226	.220	3.784	4.668

Profile Plots

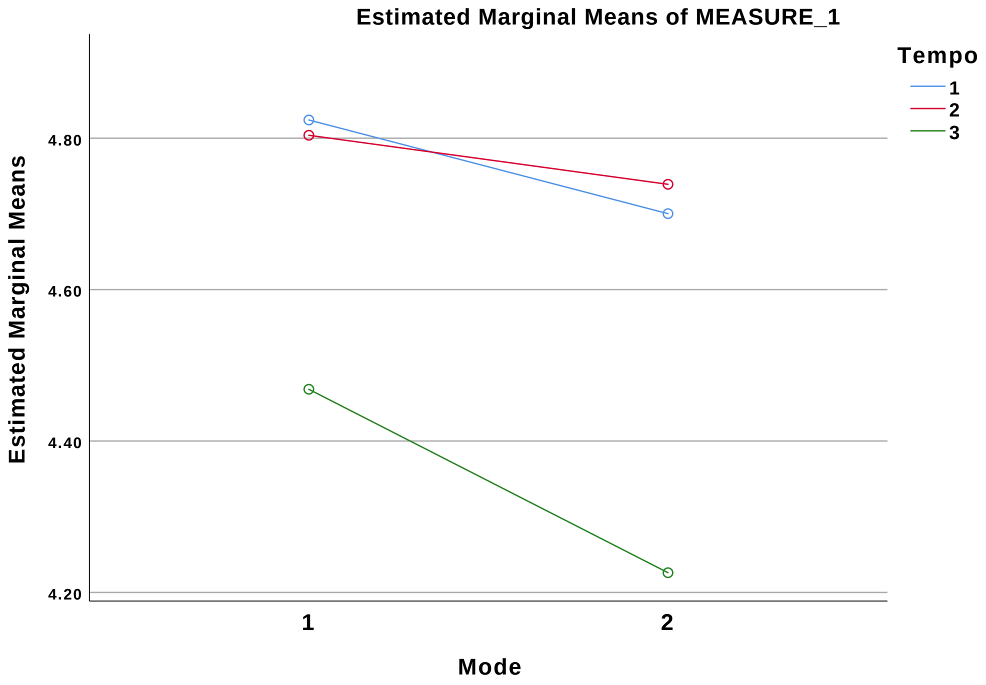

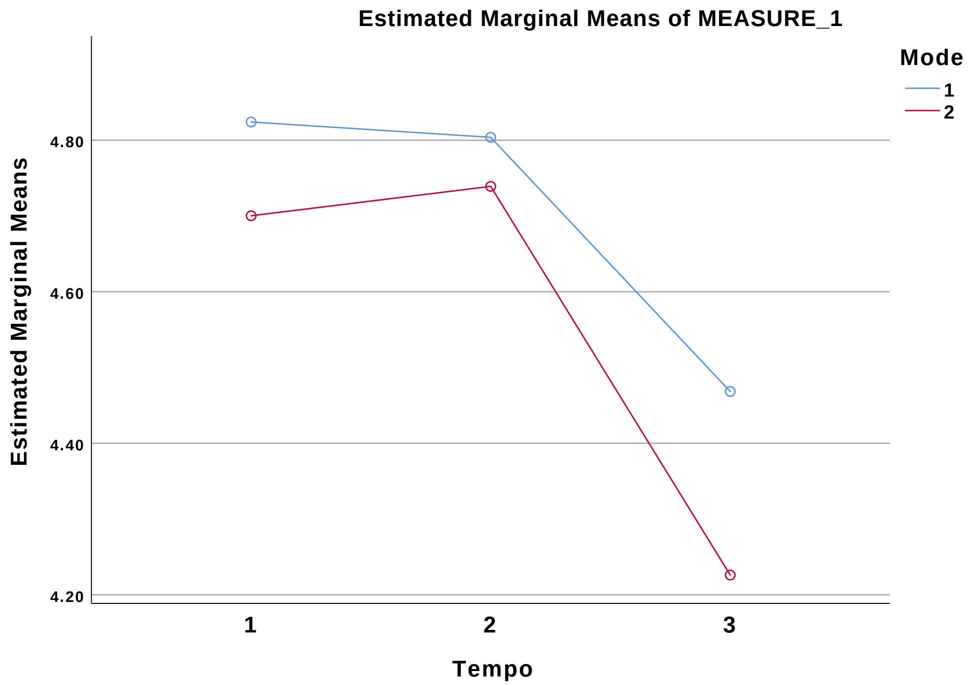


```
CORRELATIONS
 /VARIABLES=MajF_HA Avg_intensitiy_MajF_HA
 /PRINT=ONETAIL NOSIG
 /STATISTICS DESCRIPTIVES
 /MISSING=PAIRWISE.
```

Correlations

Notes

Output Created		24-MAR-2018 18:11...
Comments		
Input	Data	/Users/tayyulingrosabel/Desktop/FYP /Data to submit /Data files and analysis output/SPSS data.sav
	Active Dataset	DataSet1
	Filter	<none>
	Weight	<none>
	Split File	<none>
	N of Rows in Working Data File	52
Missing Value Handling	Definition of Missing	User-defined missing values are treated as missing.
	Cases Used	Statistics for each pair of variables are based on all the cases with valid data for that pair.
Syntax		CORRELATIONS /VARIABLES=MajF_HA Avg_intensitiy_MajF_HA /PRINT=ONETAIL NOSIG /STATISTICS DESCRIPTIVES /MISSING=PAIRWISE.
Resources	Processor Time	00:00:00.01
	Elapsed Time	00:00:00.00

Descriptive Statistics

	Mean	Std. Deviation	N
MajF_HA	6.29	2.965	49
Avg_intensitiy_MajF_HA	4.8240	1.43214	49

Correlations

		MajF_HA	Avg_intensitiy_MajF_HA
MajF_HA	Pearson Correlation	1	.440**
	Sig. (1-tailed)		.001
	N	49	49
Avg_intensitiy_MajF_HA	Pearson Correlation	.440**	1
	Sig. (1-tailed)	.001	
	N	49	49

**. Correlation is significant at the 0.01 level (1-tailed).

```
CORRELATIONS
 /VARIABLES=MajO_HA Avg_intensitiy_MajO_HA
 /PRINT=ONETAIL NOSIG
 /STATISTICS DESCRIPTIVES
 /MISSING=PAIRWISE.
```

Correlations

Notes

Output Created		24-MAR-2018 18:11...
Comments		
Input	Data	/Users/tayyulingrosabel/Desktop/FYP /Data to submit /Data files and analysis output/SPSS data.sav
	Active Dataset	DataSet1
	Filter	<none>
	Weight	<none>
	Split File	<none>
	N of Rows in Working Data File	52
Missing Value Handling	Definition of Missing	User-defined missing values are treated as missing.
	Cases Used	Statistics for each pair of variables are based on all the cases with valid data for that pair.
Syntax		CORRELATIONS /VARIABLES=MajO_HA Avg_intensitiy_MajO_HA /PRINT=ONETAIL NOSIG /STATISTICS DESCRIPTIVES /MISSING=PAIRWISE.
Resources	Processor Time	00:00:00.01
	Elapsed Time	00:00:00.00

Descriptive Statistics

	Mean	Std. Deviation	N
MajO_HA	6.22	1.982	49
Avg_intensitiy_MajO_HA	4.8038	.90023	49

Correlations

		MajO_HA	Avg_intensitiy_MajO_HA
MajO_HA	Pearson Correlation	1	.121
	Sig. (1-tailed)		.204
	N	49	49
Avg_intensitiy_MajO_HA	Pearson Correlation	.121	1
	Sig. (1-tailed)	.204	
	N	49	49

```
CORRELATIONS
  /VARIABLES=MajS_HA Avg_intensitiy_MajS_HA
  /PRINT=ONETAIL NOSIG
  /STATISTICS DESCRIPTIVES
  /MISSING=PAIRWISE.
```

Correlations

Notes

Output Created		24-MAR-2018 18:12...
Comments		
Input	Data	/Users/tayyulingrosabel/Desktop/FYP /Data to submit /Data files and analysis output/SPSS data.sav
	Active Dataset	DataSet1
	Filter	<none>
	Weight	<none>
	Split File	<none>
	N of Rows in Working Data File	52
Missing Value Handling	Definition of Missing	User-defined missing values are treated as missing.
	Cases Used	Statistics for each pair of variables are based on all the cases with valid data for that pair.
Syntax		CORRELATIONS /VARIABLES=MajS_HA Avg_intensitiy_MajS_HA /PRINT=ONETAIL NOSIG /STATISTICS DESCRIPTIVES /MISSING=PAIRWISE.
Resources	Processor Time	00:00:00.01
	Elapsed Time	00:00:00.00

Descriptive Statistics

	Mean	Std. Deviation	N
MajS_HA	3.57	2.264	49
Avg_intensitiy_MajS_HA	4.4684	1.67383	49

Correlations

		MajS_HA	Avg_intensitiy_MajS_HA
MajS_HA	Pearson Correlation	1	.359**
	Sig. (1-tailed)		.006
	N	49	49
Avg_intensitiy_MajS_HA	Pearson Correlation	.359**	1
	Sig. (1-tailed)	.006	
	N	49	49

**. Correlation is significant at the 0.01 level (1-tailed).

```
CORRELATIONS
  /VARIABLES=MinF_HA Avg_intensitiy_MinF_HA
  /PRINT=ONETAIL NOSIG
  /STATISTICS DESCRIPTIVES
  /MISSING=PAIRWISE.
```

Correlations

Notes

Output Created		24-MAR-2018 18:12...
Comments		
Input	Data	/Users/tayyulingrosabel/Desktop/FYP /Data to submit /Data files and analysis output/SPSS data.sav
	Active Dataset	DataSet1
	Filter	<none>
	Weight	<none>
	Split File	<none>
	N of Rows in Working Data File	52
Missing Value Handling	Definition of Missing	User-defined missing values are treated as missing.
	Cases Used	Statistics for each pair of variables are based on all the cases with valid data for that pair.
Syntax		CORRELATIONS<br>/VARIABLES=MinF_HA Avg_intensitiy_MinF_HA<br>/PRINT=ONETAIL NOSIG<br>/STATISTICS DESCRIPTIVES<br>/MISSING=PAIRWISE.
Resources	Processor Time	00:00:00.01
	Elapsed Time	00:00:00.00

Descriptive Statistics

	Mean	Std. Deviation	N
MinF_HA	9.20	2.901	49
Avg_intensitiy_MinF_HA	4.7003	.89883	49

Correlations

		MinF_HA	Avg_intensitiy_MinF_HA
MinF_HA	Pearson Correlation	1	.193
	Sig. (1-tailed)		.092
	N	49	49
Avg_intensitiy_MinF_HA	Pearson Correlation	.193	1
	Sig. (1-tailed)	.092	
	N	49	49

```
CORRELATIONS
  /VARIABLES=MinO_HA Avg_intensitiy_MinO_HA
  /PRINT=ONETAIL NOSIG
  /STATISTICS DESCRIPTIVES
```

Correlations

Notes

Output Created		24-MAR-2018 18:12...
Comments		
Input	Data	/Users/tayyulingrosabel/Desktop/FYP /Data to submit /Data files and analysis output/SPSS data.sav
	Active Dataset	DataSet1
	Filter	<none>
	Weight	<none>
	Split File	<none>
	N of Rows in Working Data File	52
Missing Value Handling	Definition of Missing	User-defined missing values are treated as missing.
	Cases Used	Statistics for each pair of variables are based on all the cases with valid data for that pair.
Syntax		CORRELATIONS /VARIABLES=MinO_HA Avg_intensitiy_MinO_HA /PRINT=ONETAIL NOSIG /STATISTICS DESCRIPTIVES /MISSING=PAIRWISE.
Resources	Processor Time	00:00:00.00
	Elapsed Time	00:00:00.00

Descriptive Statistics

	Mean	Std. Deviation	N
MinO_HA	6.43	2.638	49
Avg_intensitiy_MinO_HA	4.7391	.83757	49

Correlations

		MinO_HA	Avg_intensitiy_MinO_HA
MinO_HA	Pearson Correlation	1	.201
	Sig. (1-tailed)		.083
	N	49	49
Avg_intensitiy_MinO_HA	Pearson Correlation	.201	1
	Sig. (1-tailed)	.083	
	N	49	49

```
CORRELATIONS
 /VARIABLES=MinS_HA Avg_intensitiy_MinS_HA
 /PRINT=ONETAIL NOSIG
 /STATISTICS DESCRIPTIVES
 /MISSING=PAIRWISE.
```

Correlations

Notes

Output Created		24-MAR-2018 18:12...
Comments		
Input	Data	/Users/tayyulingrosabel/Desktop/FYP /Data to submit /Data files and analysis output/SPSS data.sav
	Active Dataset	DataSet1
	Filter	<none>
	Weight	<none>
	Split File	<none>
	N of Rows in Working Data File	52
Missing Value Handling	Definition of Missing	User-defined missing values are treated as missing.
	Cases Used	Statistics for each pair of variables are based on all the cases with valid data for that pair.
Syntax		CORRELATIONS /VARIABLES=MinS_HA Avg_intensitiy_MinS_HA /PRINT=ONETAIL NOSIG /STATISTICS DESCRIPTIVES /MISSING=PAIRWISE.
Resources	Processor Time	00:00:00.01
	Elapsed Time	00:00:00.00

Descriptive Statistics

	Mean	Std. Deviation	N
MinS_HA	4.35	2.840	49
Avg_intensitiy_MinS_HA	4.2261	1.53817	49

Correlations

		MinS_HA	Avg_intensitiy_MinS_HA
MinS_HA	Pearson Correlation	1	.414**
	Sig. (1-tailed)		.002
	N	49	49
Avg_intensitiy_MinS_HA	Pearson Correlation	.414**	1
	Sig. (1-tailed)	.002	
	N	49	49

**. Correlation is significant at the 0.01 level (1-tailed).
